# Context-aware sequence-to-function model of human gene regulation

**DOI:** 10.1038/s41467-026-75527-2

**Published:** 2026-07-14

**Authors:** Ekin Deniz Aksu, Martin Vingron

**Affiliations:** Department of Computational Molecular Biology, Max Planck Institute for Molecular Genetics, Berlin, Germany

**Keywords:** Machine learning, Gene expression, Computational models, Epigenomics

## Abstract

Sequence-to-function models have been very successful in predicting gene expression, chromatin accessibility, and epigenetic marks from DNA sequences alone. However, current state-of-the-art models have a fundamental limitation: they cannot extrapolate beyond the cell types and conditions included in their training dataset. Here, we introduce Corgi, a context-aware sequence-to-function model that overcomes this limitation by integrating DNA sequence and *trans*-regulator expression to predict chromatin accessibility, histone modifications, and gene expression coverage, even in held-out cell types. Trained on a diverse set of bulk and single-cell sequencing datasets, Corgi achieves top performance in joint cross-sequence and cross-cell-type epigenetic track prediction. Additionally, we present an advanced model version, Corgi+, which is state-of-the-art in imputation of epigenetic tracks using only RNA-seq data. We further show that Corgi learns key cell type-specific *trans*-regulators in a zero-shot manner, and it can predict genomic variant effects in held-out cell types.

## Abstract (web summary)

This study introduces Corgi and Corgi+ , biology-inspired deep neural network models of regulatory DNA sequences that can generalize to unseen cell types. They accurately impute epigenomic data from RNA-seq experiments, identify key cell type-specific regulators and predict genomic variant effects.

## Introduction

A central goal of genomics is to understand how the genome generates its functional outputs, such as gene expression and epigenetic state. To this end, sequence-to-function models have been highly successful in making such predictions from DNA sequences alone^1^. Trained on the human and mouse reference genomes and large functional genomics datasets, these models learn gene regulatory grammar and can accurately predict the activity of previously unseen sequences^2–4^. They are useful in predicting effects of non-coding variants and generating synthetic sequences^5–7^. However, current state-of-the-art models cannot extrapolate to unseen cell types and conditions. Since they operate on DNA sequence alone, these models are blind to the biological context in which the sequence is interpreted.

Context-aware sequence-to-function models overcome this fundamental limitation by enabling generalization to previously unseen cellular contexts. These models make use of not only the DNA sequence but also a context vector. The context vector is computed from knowledge about the cell state: usually gene expression or chromatin accessibility. Previous models used transcription factor (TF) expression^8–11^, ATAC-seq^12,13^, and single-cell multiome (joint RNA and ATAC)^14^ data. However, these models are limited either in their predictive performance, low number of predicted assay types, or reliance upon known TF binding motifs.

Here we introduce **Corgi** (**Co**ntext-aware **R**egulatory **G**enomics **I**nference), a context-aware sequence-to-function model that predicts genome-wide coverage of 16 different assays including RNA-seq, chromatin accessibility, DNA methylation and histone modifications in human cells. Importantly, we designed Corgi’s architecture to imitate cellular gene regulation. In the cell, *trans-*regulatory proteins bind DNA and RNA to drive transcription, modulate chromatin state, and control mRNA decay. So for its context vector, Corgi uses the expression of *trans-*regulators, which are defined as transcription factors, transcriptional co-activators, chromatin modifiers, and RNA-binding proteins.

We address a central challenge in context-aware sequence-to-function models: it is not straightforward to integrate DNA sequence with the context vector due to the difference in their dimensions. The context vector is one-dimensional, while the DNA sequence has two dimensions: length and features. Existing methods have used feature concatenation (ChromDragoNN^9^, EpiGePT^11^, EnformerCelltyping^15^) and cell type-specific decoders (scooby^14^) to integrate context vectors with DNA sequence information. Some methods use chromatin accessibility information for their context, which simplifies integration as accessibility can be appended to the sequence information directly as a new channel (EPCOT^16^). In our work, we leverage the feature-wise linear modulation^17^ (FiLM) technique to combine the two sources of information. FiLM was previously employed in various tasks such as image generation^18^ and speech recognition^19^, and has proven technically and conceptually appropriate for this task. Importantly, using FiLM overcomes limitations of EpiGePT, as it allows all *trans-*regulators to influence learned *cis* features, without any dependence on known transcription factor binding motifs.

After training on a diverse set of human bulk and single-cell contexts, we show that Corgi can accurately generalize to previously unseen contexts. Corgi has high accuracy in predicting gene expression in new cell types, while accurately predicting DNase-seq, ATAC-seq, DNA methylation, and various histone marks. Remarkably, Corgi’s performance remains robust even on the toughest benchmarks, which test the model on held-out sequences and held-out cell types, where Corgi significantly outperforms EpiGePT^11^. We also introduce an advanced model version called Corgi+, which is optimized for imputing epigenomic tracks in unseen cell types using RNA-seq data, and benchmark its performance against EpiGePT^11^ as well as the tensor decomposition-based imputation tool Avocado^20^. Corgi+ significantly outperforms both models, reaching state-of-the-art performance in epigenomic data imputation using RNA-seq. Finally, we demonstrate that Corgi identifies key cell type-specific *trans*-regulators in a zero-shot manner, and can predict genomic variant effects in held-out cell types.

## Results

### Model architecture and training data

**Fig. 1:**
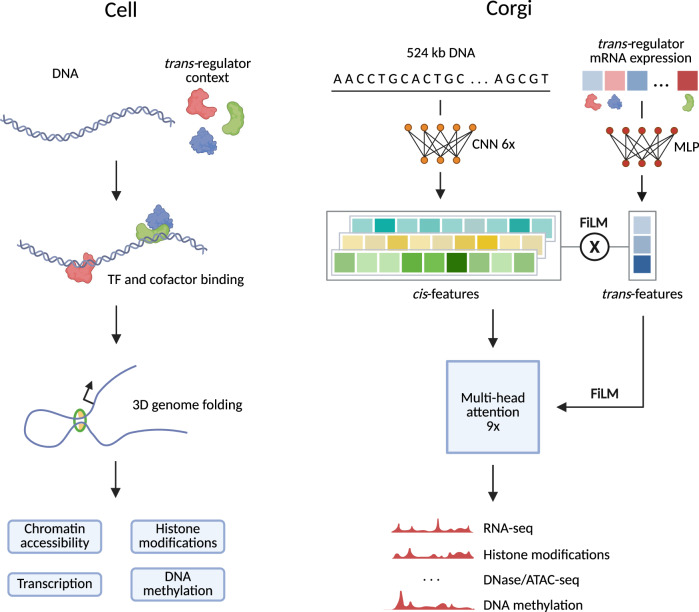
Corgi’s architecture mimics cellular gene regulation. We designed an architecture that strives to imitate how cells read the regulatory genome using *trans* factors. The model first extracts *cis*-regulatory features via convolutional layers and *trans*-regulatory features via an MLP operating on *trans-*regulator expression. These are integrated using FiLM layers, which apply feature-wise affine transformations akin to biophysical “affinity × concentration” models. Instead of single TFs, these features are combinations of *trans-*regulators learned by the model. Afterwards, transformer blocks with FiLM layers capture long-range genomic interactions in a cell type-specific manner. Created in BioRender. Aksu, E. D. (2026) https://BioRender.com/syy5etk.

Corgi builds upon a hybrid convolutional-transformer architecture, similar to recently published DNA sequence models^21^. The input 524 kb one-hot encoded DNA sequence is first processed through several layers of convolution and max pooling operations, aiming to extract meaningful sequence features and to reduce the length of the sequence by decreasing resolution (Fig. 1). This step computes the *cis*-features matrix that summarizes the local *cis*-regulatory landscape. To enable context-awareness, Corgi has a second input: the *trans*-regulatory context vector. This vector represents expression levels of selected *trans-*regulator genes, and is processed using a multilayer perceptron which computes the *trans*-features. Subsequently, *cis*- and *trans*-features are integrated by the FiLM layers, which apply an affine transformation to the *cis*-features matrix along its feature axis. These are followed by transformer layers that aim to capture long-range genomic interactions such as enhancer-promoter loops through self-attention. The final layer outputs coverage predictions for 16 different genomic assays at a 64 bp resolution. For a more detailed description of the architecture, see Methods and Fig. S1.

This design mirrors cellular gene regulation: for a regulatory element to be active, both a *cis*-element and its corresponding *trans*-regulator must be present. Mathematically, it is a multiplication of *cis*- and *trans*- features, and if either one of them is zero, the product is also zero. This is conceptually similar to models like activity-by-contact (ABC)^22^, which multiply activity with contact. In Corgi, these features can be combinations of different factors that are learned during the training process. There are FiLM layers within the convolutional blocks as well as the transformer blocks, ensuring that *trans-*regulatory context can be integrated into the sequence at multiple points.

We trained Corgi on a large, curated dataset of sequencing experiments from ENCODE^23^, FANTOM5^24^, Tabula Sapiens^25^, CATlas^26^ (Methods). The dataset includes DNase-seq, ChIP-seq, whole-genome bisulfite sequencing (WGBS), as well as bulk and single-cell ATAC-seq and RNA-seq experiments. We used a very strict training-test split to minimize potential data leakage between training, validation, and test contexts (Figure S2, Methods).

### Corgi accurately predicts genomic tracks in held-out sequences and contexts

In order to use the diverse training set most efficiently, we first harmonized those input *trans-*regulator expression levels that were obtained by different transcriptomic assays by quantile normalization, log transformation, and batch correction. We assessed the impact of harmonization by focusing on samples with at least two different transcriptomic assays available. Harmonization reduced batch effects between different assay types and improved correlation between assays, with the most striking examples being CAGE-seq vs RNA-seq. These assays both measure the concentration of mRNA in the cell and are shown to have strong correlation^27,28^ (Fig. S3).

Then, we evaluated Corgi’s performance under three different benchmarking regimes: cross-cell type, cross-sequence, and cross-both. In the cross-cell type setting, the model predicts on held-out cell types using sequences seen during training, thus testing generalization to new biological contexts. In the cross-sequence benchmark, predictions are made on unseen genomic regions within training cell types. This is the only benchmark that traditional non-context-aware models can be evaluated in. Finally, the stringent cross-both setting evaluates predictions on both unseen cell types and unseen sequences. This benchmark represents a very hard challenge for sequence-to-function models, as accurate prediction requires generalization capabilities across new cell types and new sequences. For all benchmarks, we applied test-time data augmentation by averaging predictions for slightly shifted and reverse-complemented sequences.

**Fig. 2:**
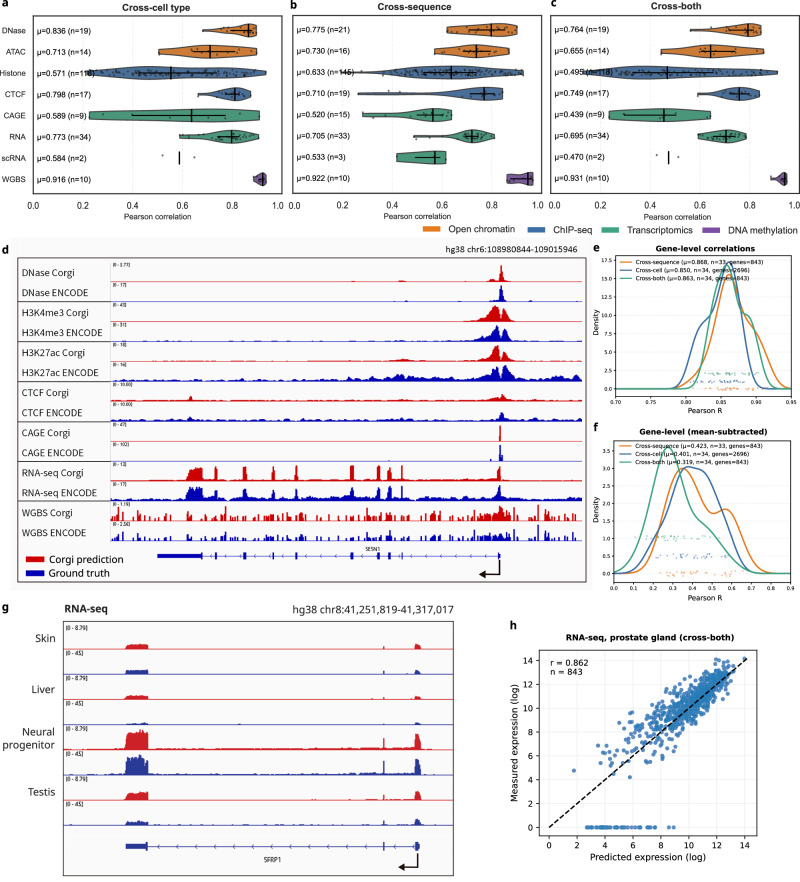
Corgi accurately predicts genomic coverage of multiple tracks in held-out cell types and sequences. **a**–**c** Violin plots showing model performance across different assays in cross-cell type, cross-sequence, and cross-both settings. Pearson correlations between predictions and ground truth data across genomic bins are reported; each data point is a correlation coefficient in one assay. Sample size (N) refers to the number of biological samples. Vertical lines indicate median values, and horizontal lines indicate interquartile ranges. **d** Corgi predictions (red) and ENCODE ground truth data (blue) are shown in an example locus from chr6 containing the *SESN1* gene in sample #323 (testis), from the cross-both benchmark. Remarkably, the model captures histone mark signal shapes, the site of the CAGE-signal, DNA methylation patterns, and exact exon-intron boundaries in RNA-seq. **e** Density plots showing the performance distribution in gene-level RNA-seq predictions. Individual data points are shown as jitter plots; for these points, the x-axis value shows the corresponding Pearson *r* value. In order to compute gene-level predictions, predicted RNA-seq coverage in gene-overlapping bins was summed and log-transformed. **f** Same data as (**e**), with Pearson correlations calculated after subtracting gene means from the data. This shows how much of the cell type-specific variation is captured by Corgi. **g** Genome browser view around the held-out gene *SFRP1*, showing predicted (red) and observed (blue) RNA-seq coverage from different held-out cell types. Corgi correctly identifies neural progenitor cells as the context where *SFRP1* is most highly expressed. **h** Scatterplot showing the relationship between predicted and measured log expression in the prostate tissue in genes from the test set. Each point represents the expression level of one gene, and the dashed line represents perfect prediction. Sample size (N) refers to the number of genes. The two-sided *p*-value tests against non-correlation. Pearson *r* value is shown. Source data are provided as a Source Data file.

Corgi has strong overall performance across a multitude of experiments (Fig. 2). In the cross-cell type setting, Corgi achieves an average Pearson’s *r* of 0.84 for DNase-seq predictions across genomic bins. The performance changes considerably across assays: histone modification ChIP-seq tracks show the highest variability, ranging from 0.39 for H3K9me3 up to 0.83 for H3K4me3 (Fig. S4). The model also predicts gene expression very accurately, achieving an average of 0.59 for CAGE and 0.79 for bulk RNA-seq tracks. Single-cell RNA-seq cannot be predicted as well as the bulk experiments, possibly due to the low abundance of data in our training set and its inherent sparsity. Finally, DNA methylation can be predicted with very high accuracy (mean *r* 0.92), and in some samples approaching near-perfect predictions. Corgi maintains strong predictive performance in the cross-sequence setting, with higher accuracy in predicting histone marks compared to the cross-cell setting (Fig. S4).

Under the most challenging cross-both setting, which involves generalization to both unseen sequences and unseen cell types, Corgi still performs robustly, reaching 0.76 for DNase-seq and 0.69 for RNA-seq. DNA methylation is predicted again with very high accuracy, reaching 0.93.

A representative genome browser view illustrates Corgi’s predictive capacity across a 35 kb region on chromosome 6, encompassing the *SESN1* locus (Fig. 2d). This example is taken from the cross-both setting and shows a held-out sequence in testis. Corgi faithfully reconstructs a wide range of assays. Corgi predictions capture both the sharper peaks characteristic of DNase-seq hypersensitive sites as well as the broader domains marked by active histone modifications such as H3K4me3 and H3K27ac. The predicted expression landscape across *SESN1* closely mirrors experimental RNA-seq and CAGE signals, with the exon-intron boundaries accurately predicted in the RNA-seq track and the exact TSS predicted in the CAGE track. Finally, the model can also recapitulate DNA methylation patterns with high precision.

For gene expression prediction, it is often more informative to use log-transformed TPM values instead of bin-level RNA-seq coverage across the gene. To simulate this, we computed gene-level expression predictions by aggregating coverage predictions over annotated genes and applied a log-transformation. Corgi achieves an average gene-level Pearson’s *r* of 0.85 in the cross-cell type setting, and 0.87 in the cross-sequence and 0.86 in the cross-both settings (Fig. 2e, h). Importantly, Corgi’s performance is robust in different benchmarks and does not drop off when generalizing to held-out sequences or cell types.

In order to assess Corgi’s ability to uncover cell type-specific gene expression beyond the mean expression across contexts, we also calculated mean-subtracted gene-level correlations. In this challenging task, Corgi has a mean Pearson’s *r* of 0.40 in the cross-cell type setting, 0.42 in the cross-sequence setting, and 0.32 in the cross-both setting, showing that it is able to partly understand cell type-specific gene regulation and expression (Fig. 2f). The performance is slightly higher in rank correlation metrics, with correlations increasing to 0.43, 0.48 and 0.36 in cross-cell type, cross-sequence and cross-both respectively (Fig. S5). As a concrete example around a specific locus, we looked at RNA expression in four held-out contexts at the held-out *SFRP1* locus, a regulator of neural development in the mouse midbrain^29^. Corgi correctly predicts gene expression levels of *SFRP1* and its upregulation in neural progenitor cells (Fig. 2g).

### Corgi improves the state-of-the-art in context-aware epigenetic predictions

**Fig. 3:**
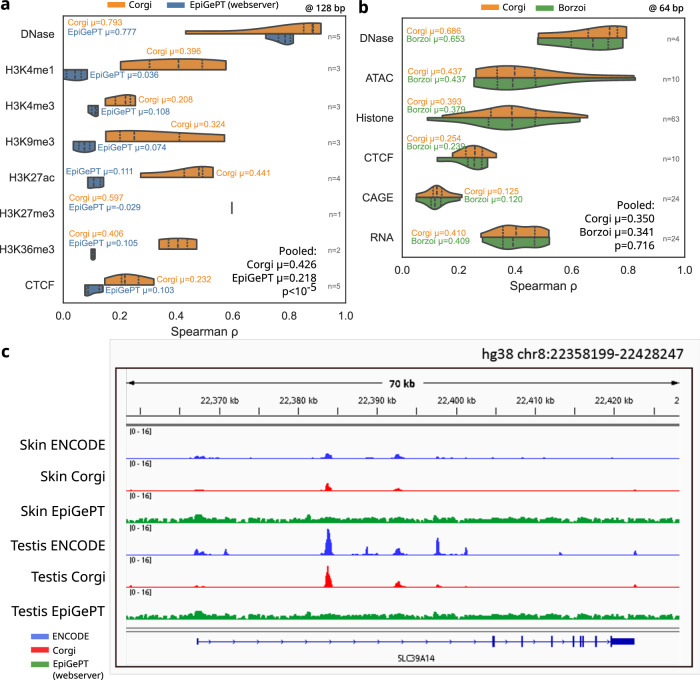
Comparison to EpiGePT and Borzoi. Distribution of Spearman correlation coefficients comparing **a** Corgi and EpiGePT at 128 bp resolution, and **b** Corgi and Borzoi at 64 bp resolution. Pooled comparisons were performed by two-sided, paired Wilcoxon signed-rank tests. For Corgi vs EpiGePT, the exact *p*-value is 7.540e−06. Sample size corresponds to different biological samples. Mean results are reported next to the violin plots. Mean Spearman correlations across all samples and tracks (pooled) and a two-sided Wilcoxon-rank sum test *p*-value are reported in the inset. **c** Example locus showing EpiGePT (webserver) (green) and Corgi (red) predictions versus the ground truth DNase-seq signal from ENCODE (blue). The sample (skin, testis) and sequence combination is cross-validated for Corgi, while EpiGePT (webserver) performs random splits. Source data are provided as a Source Data file.

We benchmarked Corgi against EpiGePT^11^, a recent context-aware model for epigenetic track predictions. EpiGePT is the latest in a line of models that use DNA sequence and TF expression to predict epigenetic tracks. It uses the RNA expression values of transcription factors as its cell state vector, similar to Corgi. However, EpiGePT also relies upon known TF binding motifs to scan the input DNA sequence and calculate binding affinities. To compute predictions, we used the EpiGePT webserver, which uses an EpiGePT checkpoint trained on ENCODE data. Corgi clearly surpassed EpiGePT (webserver), reaching an average of 0.426 versus 0.218 on all tracks and samples (Fig. 3a). Furthermore, visual inspection in the genome browser shows that EpiGePT (webserver) predictions have higher noise, and DNase-seq peaks are often not correctly identified (Fig. 3c).

We next compared Corgi to the sequence-to-function model Borzoi^3^ in a cross-sequence setting. Overall, bin-level performance was identical across almost all measured tracks (Fig. 3b, S6). Given that discrepancies in Pearson’s *r* for some tracks may reflect minor differences in output signal scaling (squashed scale) between Corgi and Borzoi, we consider Spearman’s *ρ* to be a more robust metric than Pearson’s *r*. In gene-level expression correlations, Borzoi has a measurable edge over Corgi (0.87 vs 0.84, Fig. S5c) as well as in mean-subtracted gene-level correlations (0.47 vs 0.42, Fig. S5d).

### Cross-cell type RNA-to-epigenomic data imputation with Corgi+ 

Even though there are thousands of possible cell type-epigenomic assay combinations, only a portion of this space has been experimentally tested. Therefore, it is of broad interest to impute the missing datasets, using available data from the chosen cell type and the chosen assay^30^. Current tools view data as a 3D tensor with cell types, assays, and genomic position as its dimensions, and use tensor decomposition (PREDICTD^31^) and deep tensor factorization (Avocado^20^). These tensor-based methods are fundamentally different from sequence-to-function models, in the sense that they do not explicitly use DNA sequence as an input, but they extract embeddings from genomic positions.

Since Corgi was designed with cross-both generalization in mind, it is not optimized for the data imputation setting. Critically, Corgi generalizes to unseen DNA sequences; therefore, it cannot use the “local” RNA-seq coverage track, i.e., signal from the 524 kb input region, even though RNA-seq data is available. Additionally, in the imputation setting, baseline mean signal from training contexts can be passed as an additional input, which was shown to increase DNase-seq imputation performance^9^.

**Fig. 4:**
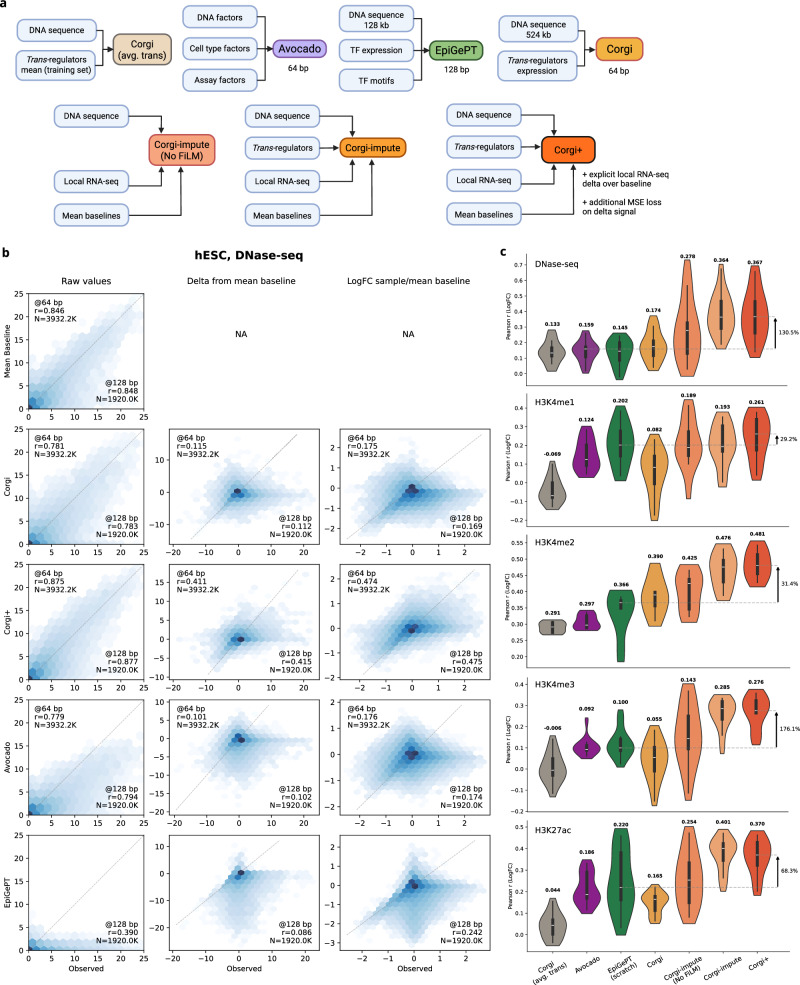
Corgi+ accurately imputes epigenomic data using RNA-seq. **a** Experimental setup that shows input data for the seven models trained for cross-cell type data imputation. Created in BioRender. Aksu, E. D. (2026) https://BioRender.com/syy5etk**b** Hexagonal binned plots showing the distribution of predicted (y-axis) versus observed (x-axis) DNase-seq coverage values in human embryonic stem cells (hESC) at bin-level resolution. 64 bp resolution was used for rows 1-4, while 128 bp was used for row 5 (EpiGepT). Pearson correlation at 64 and 128 bp resolutions is reported in the plots. Dashed lines show perfect prediction. Sample size (N) refers to the number of bins. **c** Comparison of model performance across cell types (*n* = 14) in selected assays, using Pearson correlations between ground truth and predicted logFC over the mean baseline. Median values are reported on top of the violin plots. The boxes show the quartiles, and the whiskers extend until the furthest data point within 1.5 * interquartile range from both quartiles. Dashed lines and the arrow show relative improvement of Corgi+ with respect to the highest performance from Avocado or EpiGePT. Source data are provided as a Source Data file.

In order to test the effects of these optimizations on data imputation performance, and to compare sequence-based models to a tensor-based model, we trained multiple models on a subset of the Corgi training data (Fig. 4a). As a baseline, we included “*Corgi (avg. trans)*”, which uses the average training *trans*-regulator expression regardless of the chosen cell type. Further, we trained “*Corgi-impute*”, which uses local RNA-seq coverage tracks at 64 bp resolution, as well as the mean training baseline signal from all Corgi tracks. The mean training baseline signal is assay-specific, and simply averages signal from a given assay across samples in the training set. For example, for the DNase-seq channel in a given genomic window, it corresponds to the average of observed DNase-seq signal in the same window across all cell types in the training set. In order to test the effect of conditioning on *trans*-regulator expression, we trained “*Corgi-impute (No FiLM)*”. Finally, we designed a model that we call Corgi + , with additional imputation-optimized input and objective functions. We compared the imputation performance of the Corgi family to Avocado and EpiGePT (scratch), which we trained from scratch and validated on the same training and validation data as *Corgi-impute* and Corgi+.

Since genomic data is highly correlated between different cell types, the mean baseline signal from training contexts is a good predictor of raw coverage values in the new cell type, and model predictions often fall short of this baseline. As an example, in DNase-seq predictions in human embryonic stem cells (hESC), mean baseline has the second-best accuracy after Corgi+ (*r* = 0.846 vs 0.875, Fig. 4b, also Figure S11). In raw correlations, the only outlier is EpiGePT, which does not match the correct scale, possibly due to using MSE loss rather than a Poisson-based loss. Contrary to using raw coverage values, subtracting the mean baseline and using the delta signal for correlation computations is a much more informative metric, since we are interested in regions with cell type-specifically increased or decreased signal compared to all other cell types. In this metric, Corgi+ shows strong performance in predicting DNase-seq signals (*r* = 0.411, Fig. 4b 2nd column). Although since absolute delta values are generally higher in high signal regions, we consider Pearson *r* on log fold change (logFC) over mean baseline to be the most appropriate metric in this benchmark (Corgi+ *r* = 0.474*)*. Interestingly, we observe that it is easier for all models to correctly predict decreases from baseline, compared to increases. We show logFC scatter plots in the last column of Fig. 4b and use this measure in the summary statistics depicted in the violin plots of Fig. 4c.

We systematically compared the performance of all models in all 14 test samples (including liver, testis, prostate, iPSC, hESC, neural progenitor, and cardiac myocyte cells differentiated in vitro) by computing the correlation between observed and predicted logFC values across samples for each assay. Corgi+ consistently showed high accuracy across all assay types with median *r* values up to 0.481 (Fig. 4c, S7, note that Fig. 4c shows data for 5 assays and Fig. S7 the additional 7 assays). For each assay, we also calculated the percentage increase between Corgi+ and the best result by Avocado or EpiGePT, where Corgi+ has significant and consistent gains, with increases in 11 out of 12 tracks (Fig. 4c and S7).

As expected, the *Corgi (avg. trans)* baseline does not have good performance, supporting that the chosen metric is correct, since this baseline is not expected to contain any cell type-specific information. Interestingly, when comparing *Corgi-impute* to *Corgi-impute (No FiLM)*, we see that using the global *trans-*regulator features increased performance in 10 out of 12 tracks (Fig. 4c and S7). One of the cases where using FiLM is detrimental was in H3K79me2 (median *r* = 0.397 vs 0.365), which is a histone mark related to active transcription from gene bodies, and therefore the local RNA-seq coverage is expected to be very informative. Nevertheless, Corgi+ still outperforms *Corgi-impute (No FiLM)* in this assay (median *r* = 0.424 vs 0.397). Finally, the performance lead of Corgi+ holds when the metric is switched to Pearson Delta, and the relative gains are increased even further (Fig. S8). Combined, our results show that Corgi+ is state-of-the-art in imputing chromatin accessibility and histone marks in held-out cell types using only RNA-seq data.

### Corgi+ uncovers key cell type-specific *trans*-regulators in a zero-shot manner

**Fig. 5:**
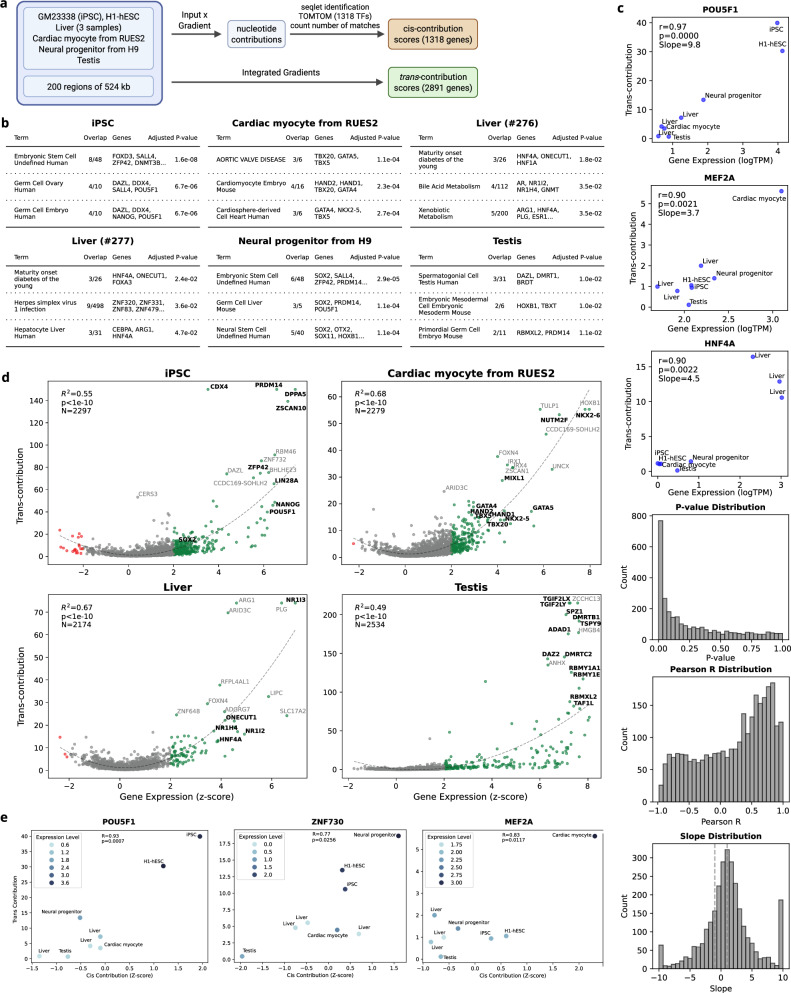
Corgi+ uncovers key cell type-specific trans-regulators in a zero shot manner. **a** Summary of the contribution score calculation setup. Created in BioRender. Aksu, E. D. (2026) https://BioRender.com/syy5etk**b** Gene set enrichment analysis results for each cell type using top 100 *trans*-contributors. Enrichment analysis was performed with Enrichr using over-representation analysis based on Fisher’s exact tests. The overlap column shows how many genes in each set were captured in the top 100 most contributing trans-regulators. Shown *p*-values are adjusted by Enrichr for multiple comparisons using the Benjamini-Hochberg procedure. **c** Relationship between gene expression and *trans*-contribution for selected genes (rows 1–3) and the global distribution of *p*-values, Pearson *r* and slope (rows 4–6). Two-sided p-values were computed using scipy.stats.pearsonr. **d** Relationship between *trans*-contribution and cell type-specific gene expression (z-score). Green and red points represent genes with high and low cell type-specific expression, respectively. Genes in the top 0.5 percentile of *trans*-contributors are annotated, while key cell type genes are marked in bold. Sample size (N) refers to the number of genes, and the dashed line is the quadratic regression fit using ordinary least squares. *P*-values were computed using the F-test, which tests the null hypothesis that both (x and x^2^) non-intercept coefficients are zero. **e** shows the relationship between *cis* and *trans* contribution scores for selected transcription factors. Color represents gene expression levels. *p*-values were computed similarly to (**c**). Source data are provided as a Source Data file.

In order to understand how Corgi+ is able to make accurate cell type-specific predictions, we calculated *cis and trans* contribution scores in eight cell types from the test set (Fig. 5a). *Cis-*contributions are based on *input x gradient* nucleotide contribution scores, which are then mapped to transcription factors using motif comparison. *Trans-*contributions are based on *integrated gradients*, which measure the impact of each *trans-*regulator feature on the absolute delta signal from the mean baseline, averaged over epigenetic tracks.

First, we asked whether predicted top *trans-*regulators are known important factors for cell type-specific activity. For each cell type, we ranked the 2891 *trans-*regulators by their *trans-*contribution score, and performed a gene set enrichment analysis (GSEA) using the top 100 genes. Strikingly, for all tested cell types, most significantly enriched terms were always specifically related to that cell type, with critical cell type-specific genes being picked up (Fig. 5b). For example, in the iPSC sample, top genes include stem cell factors such as *NANOG* and *POU5F1* (OCT4). In liver, multiple hepatocyte nuclear factors (HNF) are found. In neural progenitors, neurodevelopmental markers such as *SOX2*, *SOX11*, *HOXB1*, *OTX2*, *PRDM14* and *SALL4* are among the top contributors. In the testis, genes related to spermatogenesis such as *DAZL (“Deleted in Azospermia-Like”)* and *BRDT (“Bromodomain Testis Associated”)* are found.

Next, for each gene, we tested the correlation between *trans-*contribution scores and gene expression across cell types. Key regulators usually have higher gene expression in their particular cell types, such as *POU5F1* in stem cells, *MEF2A* in cardiac myocytes, and *HNF4A* in the liver, which correlates well with *trans*-contribution scores (Fig. 5c, rows 1–3). This trend is observed across the entire dataset. Indeed, across all 2891 genes, we see that positive correlations are enriched (Fig. 5c, rows 4–5).

A similar correlation analysis can also be performed on each cell type separately. For each cell type, after filtering out very low-expressed genes, we plotted the *trans*-contribution scores against the z-score of gene expression. Here, we used z-scores over raw expression, since cell type-specific change is more important than the baseline expression. Across all cell types, we see that important regulators often have high *trans-*contribution scores as well as high expression z-scores, while we do not observe any genes with low z-scores and high *trans-*contribution (Fig. 5d). Furthermore, the relationship between *trans*-contribution and expression z-scores can be explained by quadratic regression, which explains between 39-68% of the variance of *trans-*contribution scores.

We also tested if *cis* and *trans*-contributions are linked, i.e., whether the likelihood of finding TF binding sites within the nucleotide contribution scores increases with *trans-*contribution scores. We observed that while some TFs show this tendency, the trend does not hold across all TFs (Fig. 5e). *Cis*-contributions also do not show significant GSEA hits; however key markers such as *POU5F1* in iPSC (#1 contributor) and *MEF2C* in cardiac myocyte (#5 contributor) are picked up as top genes.

Finally, we performed some sanity checks to make sure that the relationships we have seen in this section do not come from leakage of gene expression values into the contribution scores. This is important, since we used gradient-based feature attribution methods which have the input (i.e., gene expression) as one of the factors. We performed three sanity checks. First, we calculated the same GSEA test results based on gene expression values, which shows that nonspecific genes and pathways become enriched, such as the spliceosome (Fig. S9a). Second, we calculated the distribution of slopes of the linear regression fits, which show an abundance of slopes higher than 1 (or lower than −1) (Fig. 5c, last row). This is a positive indicator, since a slope around 1 (or −1) would be explained only by the contribution of expression. Third, we repeated the analysis in Fig. 5d by replacing *trans-*contribution scores with *score divided by input*, thus eliminating direct influence of gene expression. We see that the same trend persists, with important regulators still having high scores while expression z-scores still explain up to 49% of the variance (Fig. S9b).

### Analyzing *cis* and *trans* TF contributions with in silico knockouts

Since Corgi models naturally support arbitrary perturbations of both DNA sequence and *trans-*regulator expression, we used this feature for a more precise analysis of *cis* and *trans* contributions by transcription factors. Specifically, in 320 combinations of 5 samples and 64 TFs, we compared wildtype (WT) Corgi+ predictions to *cis*-knockout (Cis KO) by substituting TF binding sites with random DNA sequence, and to *trans-*knockout (Trans KO) by setting the expression level of the TF to a lower value. This analysis offers multiple advantages over gradient-based attribution, such as increased accuracy, bin-level resolution, and directionality. Note that ground truth knockout data is not available, so this analysis only measures self-consistency of the model, rather than proving accurate knockout predictions.

**Fig. 6:**
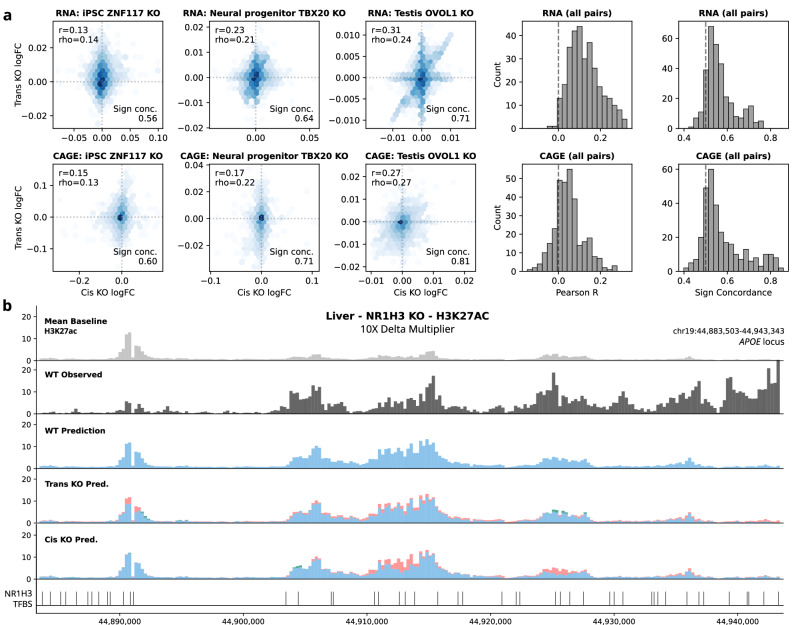
In silico knockouts link *cis* and *trans* gene regulatory effects. **a** shows the relationship between Trans KO and Cis KO logFC effects across genomic bins, in selected assays, cell types and knockouts. Pearson *r*, Spearman *rho*, and sign concordance are labeled in the subplots. The last two columns show distributions of Pearson *r* and sign concordance across all cell type and knockout pairs, when KO effects are read out from RNA and CAGE assays. **b** H3K27ac signal at an example locus containing the *APOE* gene. The grey track shows the baseline H3K27ac signal from training contexts, the black track shows the observed signal in liver. While the top light blue track shows Corgi+ predictions using the wildtype sequence and *trans*-regulator expression. The tracks for Cis KO and Trans KO predictions have the difference to WT predictions highlighted in red (decrease) and green (increase). Predicted deltas from WT are exaggerated with a multiplier of 10 for easier visualization. The bottom row shows putative *NR1H3* binding sites. Source data are provided as a Source Data file.

We assessed the correlation between Cis KO and Trans KO effects across genomic bins for all TF-cell type combinations. We report a range of relationships, for example, *ZNF117* KO in iPSCs shows a very low correlation (0.13), while *TBX20* KO in neural progenitors and *OVOL1* KO in testis shows slightly higher correlations (0.23 and 0.31 respectively) (Fig. 6a). Across all TF-cell type pairs, we see that Cis KO and Trans KO effects show a modest but positive relationship, ranging from 0 to 0.3 with RNA-seq readout. The distributions of Pearson *r* and sign concordance show a skew towards the right (Fig. 6a, columns 4–5).

For selected cell type-TF pairs, we visually investigated top genomic regions with the largest logFC between WT and KO predictions. Strikingly, we often see that genomic bins with the largest change often overlap between Cis KO and Trans KO predictions. Though it should be noted that the magnitude of effects is minuscule and differences are multiplied by a factor of 10 for better visualization. As an example, in the H3K27ac track at the *APOE* locus in liver, we observe that Corgi+ makes accurate WT predictions (light blue) compared to ground truth (dark grey), outperforming the mean baseline (light grey) (Fig. 6b). Remarkably, we see that the Cis KO and Trans KO often affect the same genomic bins, which we observed often in multiple cell type-TF pairs.

### Corgi predicts eQTL variant effects in held-out tissues

**Fig. 7:**
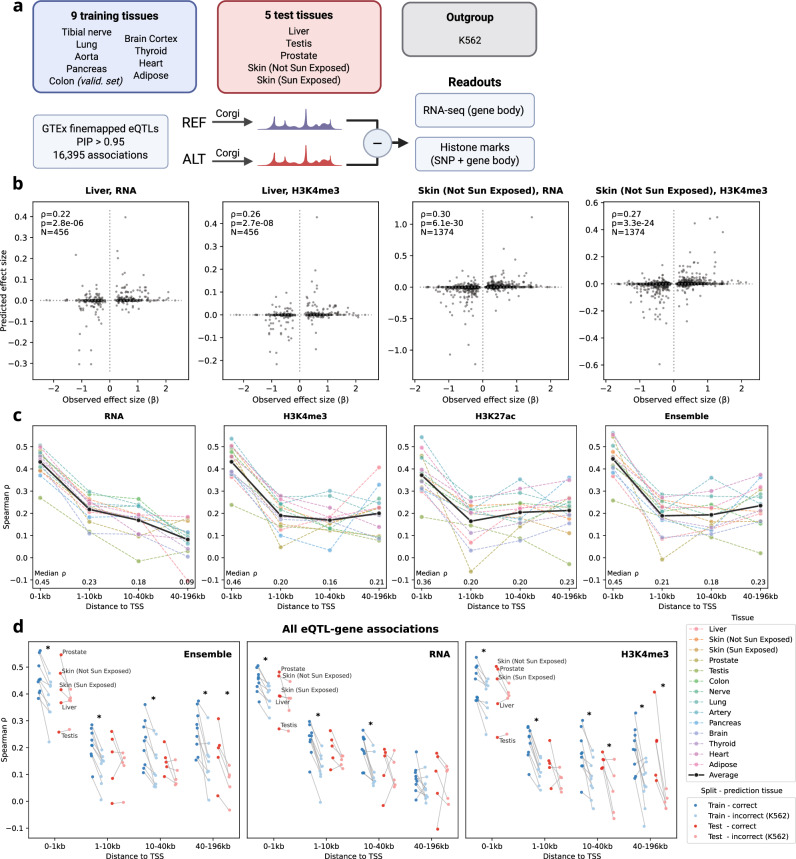
Corgi predicts eQTL variant effects in held-out tissues. **a** In selected tissues and cell types, we use Corgi to predict effects of finemapped GTEx eQTLs, with multiple readouts from the difference between outputs using alternative and reference alleles. Created in BioRender. Aksu, E. D. (2026) https://BioRender.com/syy5etk**b** shows the relationship between predicted and observed effect sizes (beta coefficient) in selected tissues and readouts. Spearman *rho* and *p*-values are reported in the plots. Sample size (N) refers to the number of fine-mapped eQTL-eGene associations in each tissue. **c** shows the relationship between correlations and distance between the eQTL and the transcription start site of the associated gene. Each colored line shows a different cell type, while the black line shows the average trend. Median rho values in distance bins are labeled above the x-axis. **d** shows changes in accuracy when *trans-*regulator expression from an incorrect cell type (light shades) is used. Asterisks show significant (*p* < 0.05) eQTL bins when comparing correct and incorrect cell types (two-sided paired *t*-test, *n* = 9 for training tissues, *n* = 5 for test tissues). Source data are provided as a Source Data file.

Sequence-to-function models can predict effects of genomic variants, which is one of their primary use cases. However, similar to genomic track predictions, this is usually limited to cell types (or tissues) within the training set. As a context-aware sequence model, Corgi has the potential to overcome this limitation. We tested Corgi’s ability to predict genomic variant effects in 9 training and 5 test cell types, on finemapped GTEx eQTLs in those cell types (Fig. 7a).

Corgi predictions are significantly correlated with the observed eQTL effect sizes in both training and held-out tissues. In some cases the RNA-seq track performs better than histone marks, and in some cases the opposite is true (Fig. 7b). We binned the associations according to the distance between the eQTL and the transcription start site (TSS) of the eGene, and plotted the correlations against this distance bin. Similar to prior observations showing that sequence models mostly ignore distal effects^32^, we see that performance degrades with increasing distance (Fig. 7c, RNA). Very interestingly, we noticed that histone mark readouts show more robust performance than RNA at long-distance effects, while having slightly lower close-range performance (Fig. 7c). This reveals a unique capability of Corgi when compared to traditional sequence-to-function models: since Corgi generates predictions for all epigenomic and transcriptomic tracks regardless of data availability, it is possible to design fixed *ensemble readouts*, combining the RNA channel with multiple histone marks. Such ensembles combine the close-range strength of RNA with long-range robustness of histone mark readouts (Fig. 7, ensemble).

Finally, we tested whether Corgi uses *trans-*regulator expression to predict cell type-specific variant effects. We selected an outgroup cell type, K562, and predicted all variant effects using the *trans-*regulator expression of this cell type. If Corgi leverages the *trans-*regulator expression in a biologically relevant manner, using the incorrect cell type to predict a variant effect measured in another cell type should decrease performance. Remarkably, for both training and test contexts, correlations between predicted and observed effect sizes were larger when using the correct cell type, showing that Corgi has some understanding of *trans-*regulators that affect cell type-specific variant effects in a zero-shot setting (Fig. 7d).

## Discussion

In this work, we refine the context-aware approach that has the potential to shift the paradigm in sequence-to-function models from using sequence alone to models that can generalize to new cell types as well as new DNA sequences. Compared to previous context-aware models, Corgi shows markedly increased accuracy, a larger context window, increased resolution, and the most extensive output experiment types.

When compared to multi-task sequence-to-function models such as Borzoi^3^ and AlphaGenome^4^, the context-aware framework offers possible advantages in using the same data in a more organized manner. The multi-task learning framework treats every genomics experiment as an independent observation. However, genomics data is highly correlated across samples: there are constitutively active regions that show a signal in many cell types and different assay types. On the other hand, if the aim is only to predict variant effects, the multi-task framework is computationally more efficient since it calculates all predictions in one forward pass. Note that having separate output channels for each genomics experiment confers a technical advantage as well: batch effects across samples can be absorbed into the weights of the final convolutional layer. In addition, while Corgi’s training framework requires each output assay type to have a reasonable number of samples in the training data, there is no such constraint in the previous framework. This allows non-context-aware models to be trained on a much larger dataset, including mouse sequences and data, and TF ChIP-seq experiments. Due to slight differences in *trans*-regulator genes, Corgi does not natively support predictions in the mouse genome; however, this could be circumvented by using orthologous genes or by fine-tuning Corgi on the mouse genome. Here we have shown that despite these technical challenges, Corgi matched the performance of Borzoi while having the additional context-aware prediction capability.

Corgi uses cellular context in the form of *trans*-regulator expression, which includes transcription factors, transcriptional co-activators, chromatin modifiers, and RNA-binding proteins. This set of genes does not represent all *trans-*regulatory elements in the human genome. We purposefully excluded microRNAs and long non-coding RNAs, as including them would limit us to using samples with available total RNA-seq experiments and discard samples with polyA tail-enriched assays, which includes most single-cell protocols.

We believe that the expression of *trans-*regulators is the most “natural” representation of the cell state for sequence-to-function models, as these regulators directly interpret the regulatory genome, modulate chromatin states, and also regulate the stability of RNA molecules. Other methods have used only transcription factors, ATAC-seq signal, or abstract representations of the entire transcriptome, each having certain drawbacks. Using ATAC-seq is a mathematically convenient approach, because it allows the context vector to be simply concatenated to the DNA sequence, yielding five channels (four for the nucleotides, one for ATAC). This is a very rich source of information. Such a model could use promoter accessibility as a baseline for gene expression predictions. However, chromatin accessibility cannot be easily manipulated experimentally, and public data availability is lower than RNA-seq. Lastly, some models use an abstract representation of the cell state (scooby^14^, DragoNNFruit^33^) which is not directly manipulatable and hard to interpret. In the end, it should be noted that since cellular gene regulation simultaneously depends on both *trans*-regulator expression *and* epigenetic memory, not using either puts an invisible upper bound on prediction accuracy.

For purposes of only cross-cell type generalization, the architecture can be optimized further, in order to use the information from RNA-seq more efficiently. We introduced Corgi+, a version of Corgi specifically designed for cross-cell type data imputation, using RNA-seq data to impute epigenomic experiments such as DNAse-seq and histone ChIP-seq. We showed that using the local RNA-seq track, integrating the global *trans-*regulator expression using FiLM, and leveraging the mean baselines significantly improve imputation performance. With these significant gains, Corgi+ is the state-of-the-art in RNA-to-epigenomic data imputation. Imputation from RNA data can be especially useful in situations where ChIP-seq is hard to perform due to sample limitations, such as in embryonic development, rare cell types, and patient tissues. Note that in cases where more than just RNA-seq data is available, tensor-based methods can use these tracks as well, while Corgi+ in its current implementation is limited to using RNA-seq only. However, this limitation could theoretically be lifted with small tweaks to model architecture and training procedure.

One of the key advantages of using *trans-*regulator expression as the cell state vector is that *trans-*regulators can be experimentally manipulated with relative ease, using plasmid constructs for overexpression, knockdown, or CRISPR knockout of key regulators. Therefore, Corgi theoretically has the ability to simulate these perturbation experiments in silico by running the model with a baseline cell type *trans-*regulator expression and manually changing the expression of selected genes to the desired value. However, we were not able to achieve accurate zero-shot perturbation predictions in practice. Even though we have shown that Corgi can identify key cell type-specific regulators and uses the *trans*-regulatory information for predicting variant effects, we also observe that Corgi predictions are not sensitive to single *trans-*perturbations. Several explanations for this phenomenon are conceivable. First, the *trans*-regulator vector is high-dimensional, and a slight change in one of the dimensions is diluted by the high dimensionality. Second, modeling of the perturbations in this manner does not take into account indirect effects of the perturbation on other *trans-*regulators. In some sense, this is akin to reading out the perturbed state only a very short time after the perturbation is administered, without waiting for the cell to reach a new stable state. However, computationally simulating second- or higher-order effects would be expensive to compute. This lack of generalization to perturbed contexts is not surprising, as *trans-*perturbation effects are out-of-distribution examples which are notoriously hard to predict, with even the most recent deep learning approaches trained on Perturb-seq^34^ data falling behind linear baselines^35^.

Ultimately linked to accurate prediction of *trans-*perturbation effects is learning of causal relationships between *trans*-regulators and DNA sequence. Causal models are increasing in popularity and will be important for in silico screening of genetic and drug perturbations in new contexts^36^. Here, we presented a way to build context-aware sequence-to-function models by integrating DNA sequence and *trans-*regulator expression information. The next step in the evolution of gene regulatory models will likely be the “causal sequence-to-function model”, which should be able to learn the causal links between abundance of *trans-*regulators, their binding to DNA, and ultimately gene expression. Important data sources for this could be TF ChIP-seq and Perturb-seq^34^ data, both of which were not used in training Corgi. Building efficient, biology-inspired deep learning architectures to integrate these data within a causal framework will be essential for deciphering the genome regulatory code.

## Methods

### Data sources

We scanned the ENCODE database for human samples that have any RNA-seq experiment, plus either one of (i) CAGE or RAMPAGE data, ii) DNase-seq and at least 1 histone modification ChIP-seq data, or (iii) any combination of at least 4 experiments. It is important that all the experiments are performed on replicates of the same sample. Data that did not comply with ENCODE data quality standards and were marked with an error tag were excluded. Six CTCF ChIP-seq samples with very poor data quality per fraction of reads in peaks (FRiP) score were excluded. In total, we identified 373 samples with these constraints (Supplementary Data 5).

We enriched our dataset with CAGE-seq data from the FANTOM5 consortium. In order to extract maximum information from the available data, we manually curated a list of samples where 65 FANTOM5 CAGE-seq experiments were matched with previously selected ENCODE samples. In addition to these, we selected 119 new samples from FANTOM5 and added them to our set (Supplementary Data 6).

In order to capture variation in *trans-*regulator expression more extensively, we expanded our set using single-cell sequencing experiments. We included 71 pseudobulked single-cell RNA-seq samples from Tabula Sapiens that are matched with single-nucleus ATAC-seq experiments from CATlas. ATAC-seq data were downloaded from CATlas directly as bigwig files, and corresponding matched gene expression data from Fu et al.^12^ was used. Finally, we used 10x multiome single-nucleus joint RNA-seq and ATAC-seq data from brain and peripheral blood mononuclear cells. Data were downloaded from 10x Genomics, and after processing and generating pseudobulks using the SEACells tool^37^, 17 samples were added to the set.

In total, we have 580 biological samples in our dataset, representing a wide range of human cells and tissues. The full list can be found in Supplementary Data 1.

### Defining *trans*-regulatory factors

To determine the set of *trans*-regulatory factors, we need to consider all genes that may play a role in gene regulation. More precisely, we need to find the genes that directly or indirectly regulate chromatin accessibility, histone modifications, chromosome folding, DNA methylation, or RNA expression. We defined this set to be transcription factors, transcriptional co-activators, chromatin modifiers, and RNA-binding proteins. Transcription factors directly bind to DNA to regulate transcription and thus constitute the first step of interpretation of regulatory regions on the genome. We used the list of TFs as compiled by the Aerts Lab^38^, which includes 1892 TF genes in the human reference genome hg38^39^. Secondly, transcriptional co-activators interact with transcription factors and regulate transcription and chromatin state, without directly binding to the DNA. The list of transcriptional co-activators was curated from the literature and contains 324 genes, including CREB binding protein, genes involved in the mediator complex, and TRIM family proteins^40,41^. Third, chromatin modifiers regulate chromatin state via histone modifications and DNA methylation, thus directly influencing many of our output tracks. We used the dbEM database, which contains 167 genes, such as histone acetyltransferases and DNA methyltransferases^42^. Finally, we decided to include all known RNA-binding proteins into the set of *trans-*regulators. Since these proteins regulate splicing, transport, and decay of RNA molecules, they directly influence RNA-seq coverage. We used a manually curated version from the RBPWorld database^43^, which includes 741 genes. After filtering out duplicate entries and genes not contained in the ENCODE RNA-seq processing pipeline, we are left with a set of 2891 *trans-*regulatory factor genes. The full list of genes can be found in Supplementary Data 2.

We have chosen gene expression as a proxy for the in vivo activity of these proteins, due to the high availability of gene expression data. The unit of gene expression is defined as the logarithm of transcript per million (TPM) values, which is ordinarily calculated from RNA-seq experiments.

### Training-test split

In order to prevent any data leakage across training and test sets, we carefully split our samples into subsets that are as dissimilar as possible. First, we organized the 580 samples into 40 clusters through agglomerative clustering according to their *trans*-regulator expression levels. Expression levels were scaled using sklearn.preprocessing.StandardScaler.fit_transform, and clustering was performed with sklearn.cluster.AgglomerativeClustering using default settings. Using manual curation, we left out entire clusters from the training set so that a suitable number of validation and test samples were reached, also making sure that all assays were available in multiple samples in the test set (Fig. S2). This curation was performed prior to any benchmarking and was not repeated. Second, for all selected validation and test samples, similar cell types or tissues still left in the training set were determined by a keyword match, and matching samples were taken out of the training set. For example, since liver is in the test set, leaving a hepatocyte sample in the training set could constitute data leakage, which we avoided. Lastly, a random sample was selected from all training clusters into an “easy-test” set, for a testing scenario where the exact samples are unseen by the model, but similar cell types and tissues were seen during training. In practice, we did not observe significant performance differences between the cross-sequence and cross-both easy-test settings (cross-sequence mean *r* = 0.408 vs cross-both easy-test 0.378, t-test *p* = 0.068, pooled all tracks; while cross-both test mean *r* = 0.343, t-test *p* = 0.000005 vs cross-sequence), suggesting that this hold-out set is almost as easy as using training cell types, and thus did not perform additional analyses on this set.

In summary, we have 488 training samples, 28 validation samples, and 37 test samples. The test set mainly consists of liver tissue, hepatocytes, testis, prostate, skin, keratinocytes, and embryonic stem cell lines.

The genomic sequences were split according to Borzoi’s folds^3^, with fold 3 reserved for testing and fold 4 reserved for validation, similar to Borzoi. Specifically, we merged overlapping sequences in the Borzoi folds bed file using bedtools merge. We made sure that sequences from different folds never overlap and merge, which is indeed the case. Then, we subtracted trans-regulator gene coordinates from the merged regions from folds 3 and 4 using bedtools subtract, which subtracts 18 Mb of sequence from fold 3 and 20 Mb from fold 4. For each merged region, we tile 524,288 bp sequences with a stride of 174,764 bp. This ensures that each base pair is represented in approximately three sequences, while also shifting the bin boundaries. At the ends of merged regions, there is unused sequence left over. If this sequence is larger than 50 kb, we add a new sequence that ends exactly at the end of the merged region. Excluding gene bodies of *trans-*regulatory factor genes from the validation and test folds is important, as their TPM values are used in the input. This procedure resulted in 11,496 sequences of length 524,288 bp for training, 1462 sequences for validation, and 1437 for testing, in a tiled fashion across most of the human reference genome (Supplementary Data 3). In total, we train on 2.2 Gbp of DNA sequence, validate on 298 Mbp, and test on 292 Mbp.

### Data preprocessing

The model uses expression levels of *trans-*regulators in units of log(TPM). Importantly, the diversity in gene expression experiments in our training data needs to be addressed because of systematic differences in TPM distributions across different assays. We have 4 types of gene expression assays in our training set: total bulk RNA-seq, polyA-capture bulk RNA-seq, pseudobulked single-cell RNA-seq, and CAGE-seq. Since we want to represent biological context, or the cell state, as a universal *trans-*regulator expression vector, ideally this should not depend on the type of gene expression assay that was performed.

In order to control for batch effects across different assays, we harmonized gene expression levels of coding genes by taking the logarithm, then quantile normalization, and finally batch correction using pyComBat^44^. Quantile normalization and batch correction were performed using the total RNA-seq data as reference, as this is the most abundant type of gene expression assay in our data. We make this reference available, which allows users to directly use TPM measurements from common RNA-seq assays without the need for further preprocessing, and the Corgi pipeline quantile normalizes them to the reference distribution.

Output signals training data: ENCODE, FANTOM5, and CATlas data were downloaded as processed in the bigwig format. For the 10x multiome experiments, data was downloaded in the h5ad and BAM formats. This data was preprocessed using scanpy^45^, celltypist^46^, multiVI^47^, sinto^48^, and deepTools^49^ in order to generate separate bigwig coverage files for each pseudobulk.

In order to normalize coverage signals across experiments with different dynamic ranges, coverage values were scaled and soft clipped with assay-specific parameters similar to the processing pipeline of Borzoi. Afterwards, coverage values were aggregated at a 64 bp resolution by taking the sum of CAGE and RAMPAGE tracks, the square root of the sum for RNA-seq tracks, and the mean for the rest of the tracks.

### Model architecture

The transformer block consists of nine layers that all use multi-head attention at a 64 bp resolution, which represents a good balance between memory cost and prediction quality, while avoiding the need for upsampling techniques such as the U-Net used in Borzoi. We use FlashAttention v2, which has speed and memory benefits compared to older implementations of multi-head attention. In total, Corgi has approximately 196 million trainable parameters.

Corgi predicts coverage tracks for the central 393 kb of the input window, in 6144 bins of 64 bp. The predicted assays are DNase-seq, ATAC-seq, ChIP-seq for multiple histone modifications (H3K4me1, H3K4me2, H3K4me3, H3K9ac, H3K9me3, H3K27ac, H3K27me3, H3K36me3, and H3K79me2), CTCF ChIP-seq, DNA methylation (whole genome bisulfite sequencing) and transcriptomic assays (CAGE-seq, RAMPAGE-seq, total RNA-seq, polyA-enriched RNA-seq, 10x scRNA-seq). Transcriptomics tracks are strand-specific (i.e., reads aligning to the positive and negative strands are split), with the exception of scRNA-seq. In total, Corgi has 22 channels in its final layer.

### Model training

We trained Corgi with an adapted version of the Poisson multinomial loss function similar to Borzoi, which decomposes the loss to magnitude and shape terms and allows for weighting the shape loss. In order to standardize the loss values across different output tracks, we balanced the loss by weighting each channel’s loss with a learnable weight parameter, inspired by Kendall et al.^50^ Thus, we minimize a new objective (poisson multinomial * weights + log[weights]) which empirically yields improved performance in some tracks, especially CAGE-seq (Figure S10).

The model was trained for 5 epochs on NVIDIA A100 GPUs with a batch size of 2, for a total of ~200 GPU-days. We used the AdamW optimizer with learning rate 0.0001 and weight decay 0.01 for the multilayer perceptron and 0.001 otherwise. Mixed precision (bfloat16) was used as it is required by FlashAttention. Furthermore, we used grouped query attention with 4 groups and 8 heads with a total of 192 dimensions. Rotary positional encodings were applied to the first 128 dimensions, similar to Flashzoi^51^.

A cosine annealing learning rate scheduler was used, which ramps up over 3000 warmup steps and then decays the learning rate. Due to the lack of computational resources, we did not perform any hyperparameter tuning, which means that the model can possibly be tuned for increased performance.

The number of epochs is considerably lower than non-context-aware models, since we consider all pairs of training sequence and training cell type as one training sample and thus our training set size becomes around 5.6 million. In one epoch, each DNA sequence is seen approximately 500 times, which can quickly lead to overfitting before the model has time to learn the rules of cell type-specific gene regulation. To combat this redundancy, we applied several dynamic augmentation techniques. First, we used the custom hg38 version from Borzoi in which Gnomad SNPs with a higher allele frequency than the hg38 reference allele were edited in. For all such alleles overlapping an input DNA sequence, each one is randomly edited in with a probability of 0.5. The reasoning is that such alleles could have been the reference allele, and they would mostly have small effects on chromatin and gene regulation, if any. Afterwards, as in previous work, input DNA sequences were randomly shifted up to 3 bp to the left or right, and then randomly reverse complemented.^3^

### Benchmarking

We assessed Corgi’s predictive performance by comparing against the ground truth data for each sample in the test set. For binwise correlations in the cross-sequence and cross-both settings, the merged test fold regions were tiled using a stride of 393 kb. This procedure generates 637 genomic regions of 524 kb for testing. For each of these input regions, Corgi predicts genomic tracks with a 64 bp resolution, resulting in prediction values for 6144 genomic bins, corresponding to the central 393 kb of the input region. Predictions from the 637 genomic regions are concatenated, resulting in 3.9 million values over which Pearson and Spearman correlation coefficients were calculated. For the cross-cell type setting, a random subset of 947 genomic regions was selected from the training folds. For gene-level benchmarking, we measured the sum of predicted vs observed coverage overlapping with gene coordinates in RNA-seq tracks, and log-transformed the values using numpy.log1p (base = e, pseudocount = 1). Genes with zero expression across all samples were excluded in order to avoid an inflation in reported accuracy values. This results in 843 genes for the cross-sequence and cross-both benchmarks, and 2696 genes for the cross-cell type benchmark (Fig. 2e, f).

We benchmarked Corgi’s performance against two different published tools: EpiGePT^11^ and Borzoi^3^. EpiGePT is a recent context-aware sequence-to-function model, and like Corgi, it also uses *trans*-regulator expression and DNA sequence. We were unable to run the provided code to train our own version of EpiGePT, and thus we used the online prediction tool at https://health.tsinghua.edu.cn/epigept/. The published model was trained on 104 cell types on hg38, including samples corresponding to tissues from our test set due to random training-test splits. Additionally, the genomic regions to be trained on were selected randomly (as opposed to leave-one-chromosome-out or homology-controlled folds), which should also give an advantage to EpiGePT. Nevertheless, we compared EpiGePT and Corgi on 5 selected cell types (brain #124, heart #192, skin #213, liver #277, testis #323) on Corgi’s test sequences. Corgi has seen some brain and heart samples during training, but not these exact samples. Skin, liver, and testis are from the Corgi test set. All five cell types were included in EpiGePT’s training set, giving EpiGePT a further advantage in this benchmark. Due to limitations of EpiGePT’s web server, only 5 cell types could be tested, and a subset of the test genomic regions were used, corresponding to 163,304 bins of 128 bp from chr8. Since Corgi has a 64 bp resolution, average pooling was applied to neighboring bins to reach a 128 bp resolution.

To ensure a fair comparison between Corgi and Borzoi in the cross-sequence setting, we selected 30 samples from our training set that matched specific tracks in the Borzoi training set (Supplementary Data File 4). Both models were trained on the same sequence folds, and fold 3 was used for testing, as before. Since Borzoi predicts at a 32 bp resolution, we applied average pooling to match the 64 bp resolution used by Corgi. The aggregation of neighboring bins in this fashion should not decrease the performance, according to the results from the original paper (see Fig. 1 from Linder et al. 2025)^3^. We used the Borzoi ensemble (4 models) from https://huggingface.co/johahi/borzoi-replicate-0 (0 through 3) and took the mean of the four model replicates as the final prediction.

### Imputation dataset, model training setup and benchmarking

We used a subset of the Corgi samples with the following inclusion criteria: available total RNA-seq, available DNase-seq, and at least one histone mark track available. 102 samples passed these criteria, with 71 from the original training set, 17 from the validation set, and 14 from the test set. Since the task is cross-cell type, all 14,395 sequences were used for training and validation.

All data from all 14,395 sequences at 64 bp resolution were concatenated and quantile normalized in order to combat experiment-specific biases and batch effects. This process was performed on each assay separately, as suggested by Schreiber et al.^30^ Mean baselines were calculated for each assay by taking the mean quantile-normalized signal from the training samples.

For Corgi and Corgi (avg. trans), the same Corgi checkpoint used in the previous section was used. So these models were trained on a larger, unnormalized dataset, and their performance should be compared to each other. Corgi-impute, Corgi-impute (No FiLM), Corgi+, EpiGePT (scratch), and Avocado were trained on the same quantile-normalized data. Corgi-impute (No FiLM) uses DNA sequence, mean baseline information (size 8196×22) and local RNA-seq information (size 8196×2, for plus and minus strand) both at 64 bp resolution. This information is added with an auxiliary encoder, which has two of the Corgi convolutional blocks, with 128 and 256 channels and kernel size 1. Afterwards, this is concatenated with DNA sequence features and goes through a linear layer to map to 1536 dimensions, followed by layer normalization and GELU. The rest of the model is the same as Corgi. Corgi-impute additionally uses the 2891-dimensional trans-regulator expression vector for the FiLM layers. Corgi+ additionally uses the RNA-seq delta from the mean baseline (8196 × 2) and has an additional MSE loss term computed on the delta output from the mean baseline. For EpiGePT, in order to make it compatible with our own dataset, we slightly modified the dataloader and trainer, without any changes to the model code. For the TF expression input, the same 711 TFs that were used in EpiGePT were used, and motifs were taken from TRANSFAC recommended motifs. Motif scanning was performed using MOODS. Avocado was trained separately on chromosomes 1 and 10, using default options. All models only had access to RNA-seq data as cell type-specific information. Hyperparameter optimization was not performed for any model.

We limited testing to the middle 512 kb so that, since EpiGePT has 128 kb input and output, the region can be divided into exactly 4 chunks, and the outputs can be concatenated afterwards. All sequences from chromosomes 1 and 10 were used in order to save on computation, since Avocado needs to be trained separately for each chromosome. Pearson R (logFC over mean baseline) was used as the main test metric and was computed as log(x + 1) - log(mean baseline + 1). To facilitate comparisons to EpiGePT with 128 bp resolution, other predictions and ground truth were reduced to 128 bp resolution using mean pooling across neighboring bins.

### Contribution score calculation

Contribution scores were calculated on a set of 200 sequences, chosen with the following procedure: First, for all sequences, we computed the absolute delta of the ground truth from the mean baseline, and summed values across the central 6144 bins (Corgi prediction region). Then, for each assay from DNase-seq, histone marks, and CTCF, we normalized these sums by min-max normalization across samples. Finally, we sum across channels, which yields one value per sequence. Since the 14,395 sequences overlap, we use every 3rd sequence to reduce overlaps, and then rank these ~5k regions by the calculated score. We selected 100 sequences randomly from the top 500, and 100 sequences randomly from the bottom ~4.5k.

*Trans*-contribution scores were calculated using Integrated Gradients from the Captum^52^ package, using the sum of absolute delta values from the mean baseline as readout. Gene set enrichment analysis of top-contributing *trans-*regulators was performed using the enrichr^53^ implementation from GSEApy^54^, using MSigDB_Hallmark_2020, KEGG_2021_Human, CellMarker_2024 and ClinVar_2025 gene sets, with adjusted *p*-value < 0.05 as significance threshold.

Nucleotide contribution scores were calculated using input x gradient from the Captum package, using the same readout as *trans*-contribution scores. Stretches of highly contributing nucleotides (seqlets) were identified using recursive_seqlets from the tangermeme^55^ package, using additional_flanks=3, threshold=1e-3, min_seqlet_len=5, max_seqlet_len=25. Identified seqlets were matched to known TRANSFAC TF binding motifs using the TOMTOM^56^ implementation from Tomtom-lite^57^, using a *p*-value threshold of 0.01, and the number of positive matches was counted for each combination of TF, cell type, and region. These counts were z-score normalized in order to extract tissue-relevant information.

### In silico mutagenesis experiments

For this experiment, a broad set of 50 sequences was selected by picking the top 6 sequences with the highest delta signal for each tissue, and then the top 10 regions with the highest mean and highest variance. For each of the selected 5 cell types and 50 sequences, we ran Corgi+ three times: i. Wildtype (WT): reference genome sequence and the basal *trans-*regulator expression of the chosen cell type, ii. *trans*-knockout (Trans KO): expression of the chosen TF set to -5, and iii. *cis-*knockout (Cis KO): putative TF binding sites in the sequence replaced by random DNA. TF binding sites were calculated using the FIMO^58^ implementation from tangermeme^55^.

This method offers several advantages over gradient-based attribution. Since gradient methods require pooling the model output to a scalar value, they do not have bin-level resolution. Furthermore, with gradient methods, using the absolute delta from the mean baseline loses directionality of the effects (not using absolute value would cancel out positive and negative effects when we pool across bins).

### Variant effect prediction

Among tissues available in GTEx with eQTL information, we selected tissues directly overlapping with samples from the Corgi dataset. These are tibial nerve, lung, aorta, pancreas, brain cortex, thyroid, heart left ventricle, and adipose from the training set and transverse colon from the validation set, which was grouped with the training tissues since other regions from the colon were available in the training set. From the test set, liver, testis, prostate, skin (sun-exposed), and skin (not sun-exposed) were available in GTEx. Also, K562 was selected as an outgroup. We downloaded GTEx fine-mapped eQTLs and limited the associations to the chosen tissues and SNPs with high posterior inclusion probability (PIP) (similar to previous work^3^) of greater than 0.95. This is to enrich the set for causal SNPs, since beta coefficients of noncausal SNPs are driven by linkage disequilibrium, which wouldn’t be detected by sequence-to-function models. This process yields 16,395 eQTL-eGene associations, with 6487 unique variants and 5121 unique eGenes.

For each association, we ran Corgi once with the 524,288 bp reference genomic sequence centered at the SNP position, using the *trans-*regulator expression of the correct tissue, and once with the same inputs except for the nucleotide at the SNP position replaced with the alternative allele, and taking the difference between the two outputs. In order to convert this matrix of size (6144,22) to a scalar effect prediction, we used multiple readouts: i. sum of plus and minus strand RNA-seq tracks at the gene body of the eGene (with 256 bp overhangs), ii. for each histone mark, sum of the signal from the region between the SNP position and the further end of the gene body (with 4096 bp overhangs). The ensemble readout score was calculated by summing readouts after normalizing each readout by dividing by abs(max(min_value, max_value)), so that each assay contributes to a similar degree to the ensemble.

### Reporting summary

Further information on research design is available in the Nature Portfolio Reporting Summary linked to this article.

## Supplementary information

Supplementary Information

Description of Additional Supplementary Files

Supplementary Data 1

Supplementary Data 2

Supplementary Data 3

Supplementary Data 4

Supplementary Data 5

Supplementary Data 6

Reporting Summary

Transparent Peer Review file

## Data Availability

Source data and model weights are provided with this paper at [https://zenodo.org/records/20686797]. ENCODE and FANTOM data download links can be found in Supplementary Data 5 and 6, respectively. CATlas data were downloaded in the bigwig format from [https://descartes.brotmanbaty.org/bbi/human-chromatin-during-development/]. Processed Tabula Sapiens RNA-seq data matched with CATlas cell types were downloaded from [s3://2023-get-xf2217/get_demo]^12^. 10x multiome data was downloaded from [https://www.10xgenomics.com/datasets/pbmc-from-a-healthy-donor-no-cell-sorting-10-k-1-standard-2-0-0] and [https://www.10xgenomics.com/datasets/frozen-human-healthy-brain-tissue-3-k-1-standard-2-0-0].

## Code availability

The Corgi source code, including the model, data processing and training scripts, can be found on GitHub at [https://github.com/ekinda/corgi]. Code to reproduce the results and figures from this paper can be found at [https://github.com/ekinda/corgi-reproduction].
