# Supplementary material for: Context-aware sequence-to-function model of human gene regulation
Source: Nat Commun. 2026 Jul 14;17:6200. doi: 10.1038/s41467-026-75527-2 (PMC13370016; doi:10.1038/s41467-026-75527-2)
Supplement: Supplementary file 1 — Supplementary Information [file 41467_2026_75527_MOESM1_ESM.pdf]

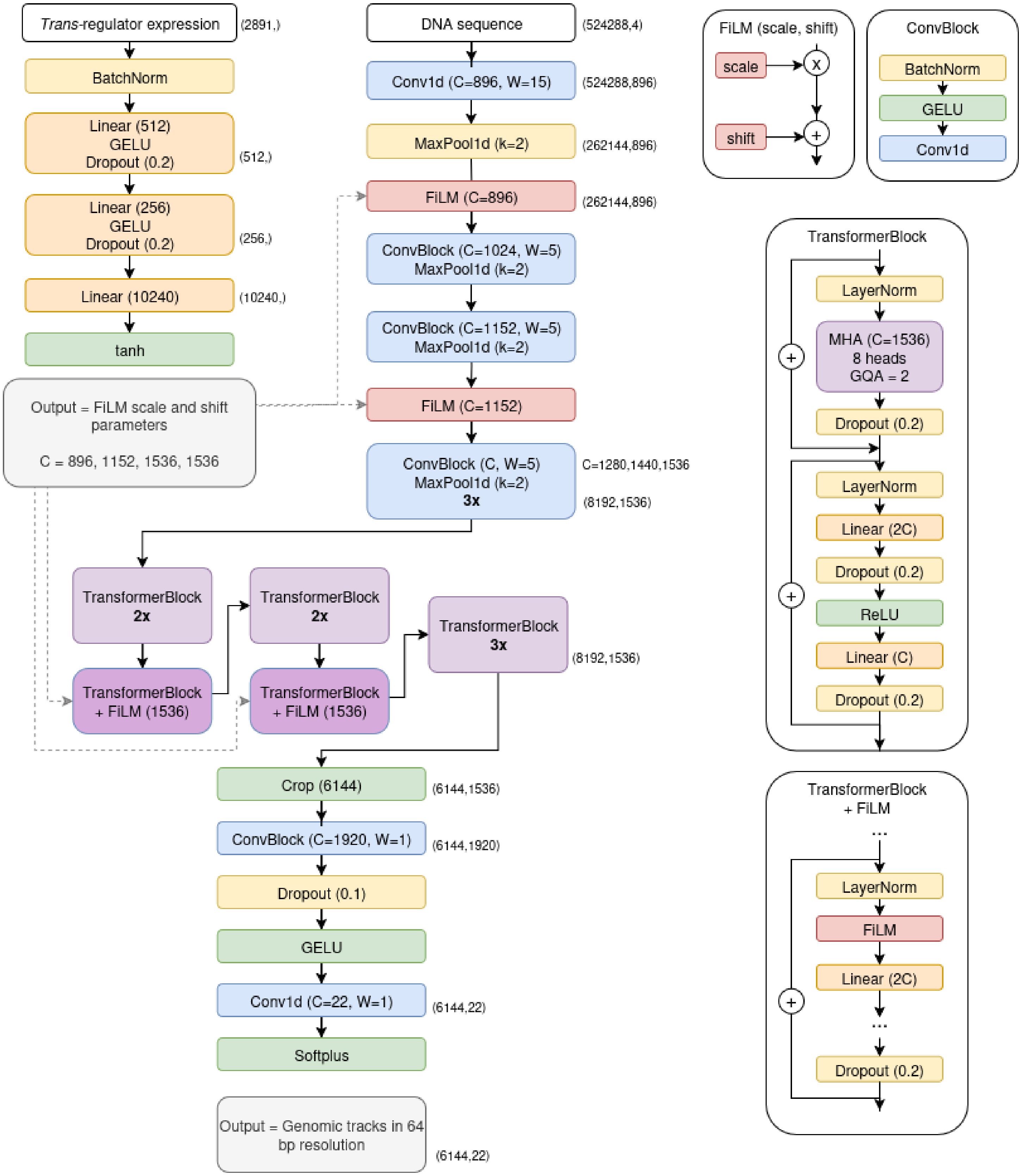

Figure S1. The full Corgi architecture

C = channels, W = convolutional kernel width, k = max pooling kernel width, GQA = grouped query attention. Dashed lines represent information flow to FiLM layers. FiLM layers themselves don't have learnable parameters, they apply an affine transformation to the input based on scale and shift parameters, which are calculated by the multilayer perceptron module. Numbers in parentheses next the boxes represent matrix sizes of inputs and at the outputs of various layers.

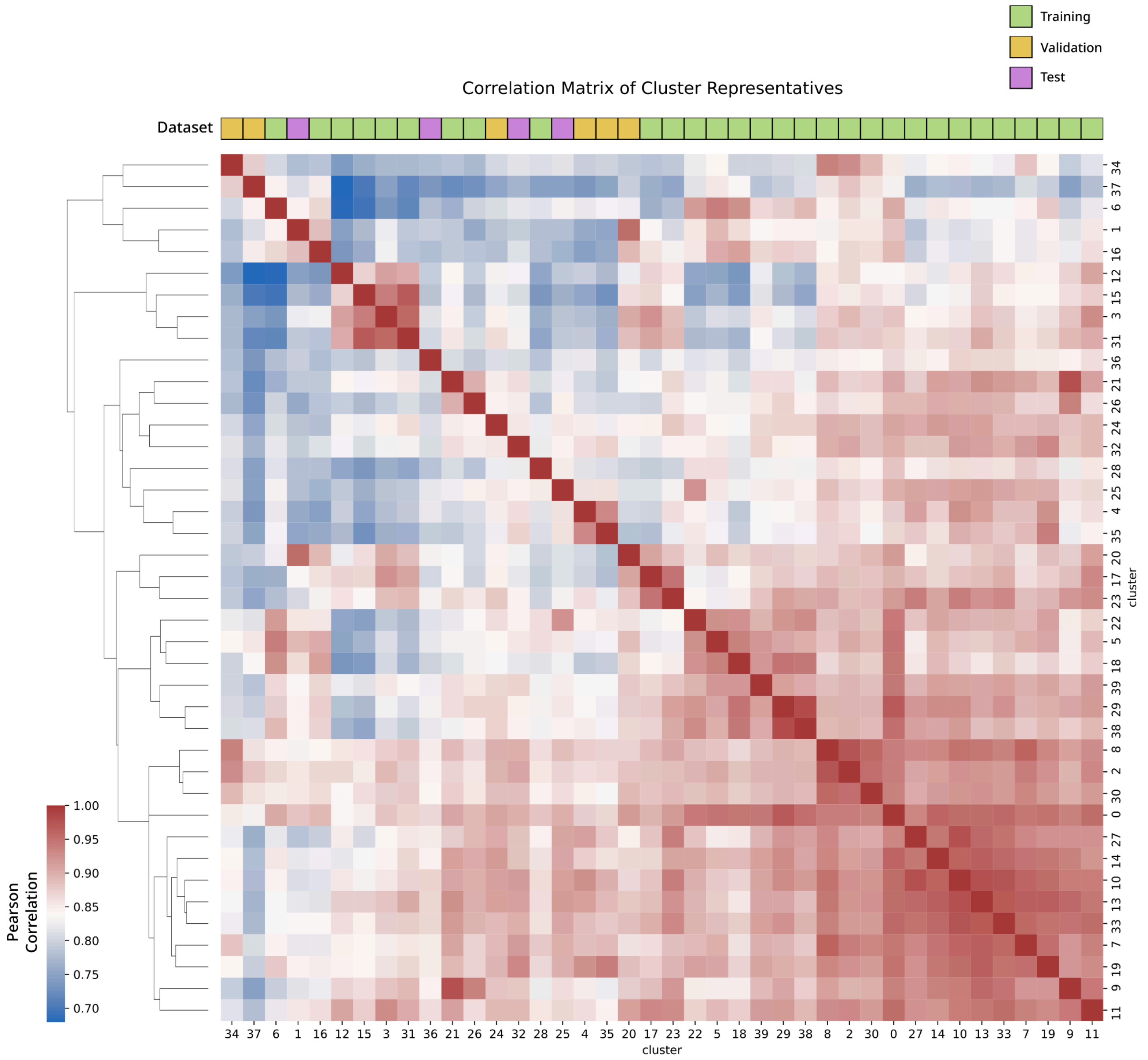

Figure S2. Similarity matrix of cluster representatives for the training/validation/test split

The heatmap shows Pearson's correlation coefficients between cluster representatives, after clustering of 580 samples into 40 clusters using an agglomerative clustering based on their gene expression values. Cluster representatives are calculated by taking the mean gene expression of all samples in a cluster. The dendrogram represents hierarchical clustering of cluster representatives, calculated by Euclidean distance. Training, validation and test samples are labeled above the heatmap. We selected validation and test clusters to minimize data leakage and variety of cell and assay types in all three datasets.

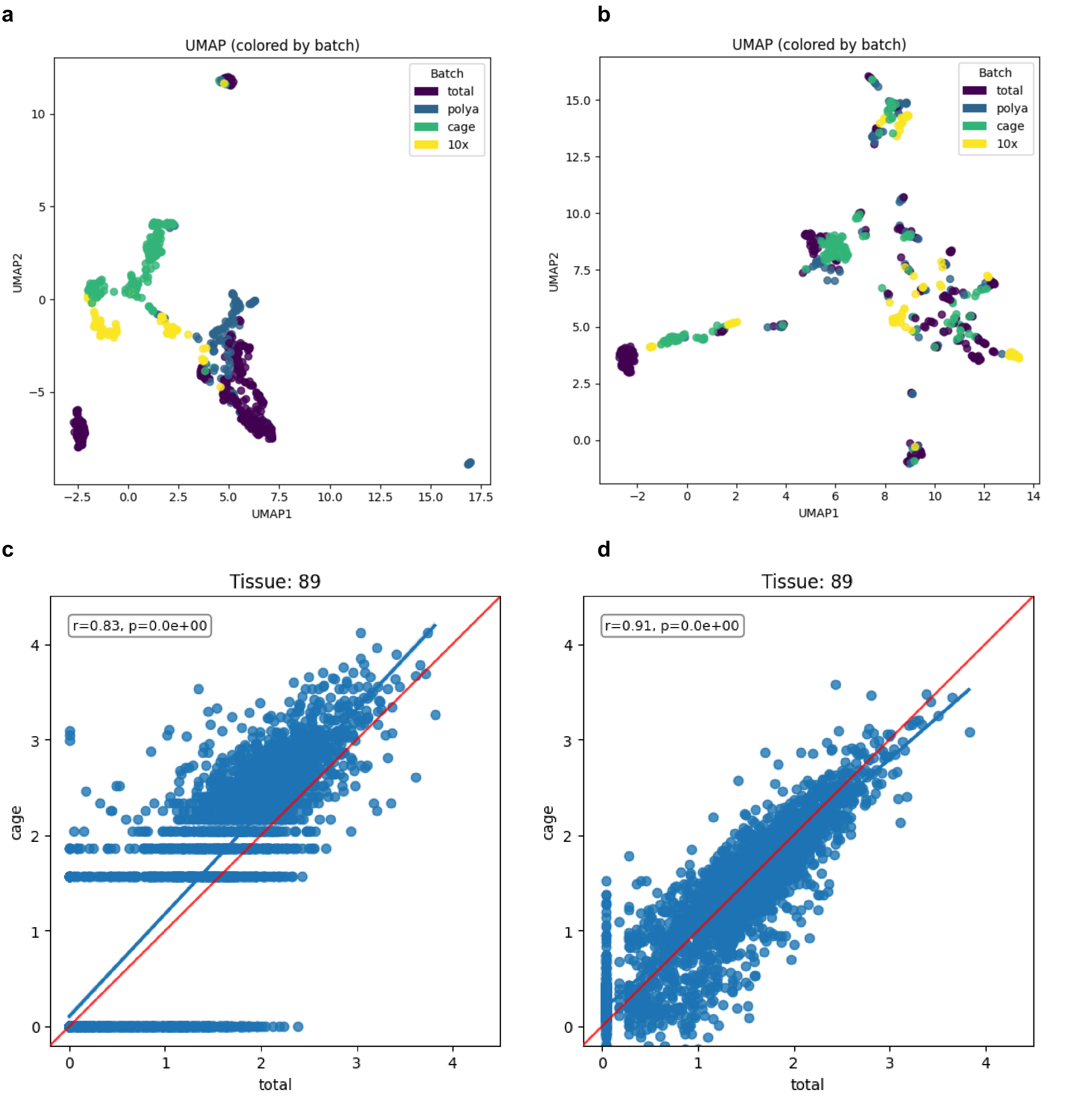

Figure S3. Harmonization reduces batch effects and improves within-sample correlation of gene expression
(a) and (b) show UMAP plots of gene expression experiments, limited to samples with at least two different assay types available (e.g. RNA-seq and CAGE), for the raw data (a) and harmonized data (b). We see that the raw gene expression values (a) cluster strongly according to their assay types, rather than their cell type. On the other hand, harmonized values (b) show markedly reduced batch effects. (c) and (d) show the relationship between RNA-seq (x-axis) and CAGE (y-axis) an example sample (#89, adrenal gland) which has total RNA-seq and CAGE data available. Raw data (c) has a Pearson's *r* value of 0.83, and the data points do not lie on the x=y line (red). After harmonization (d) we see that correlation improves to 0.91 gene expression calculated by the two assay types are closer to being equal. Two-sided p-value is testing against non-correlation.

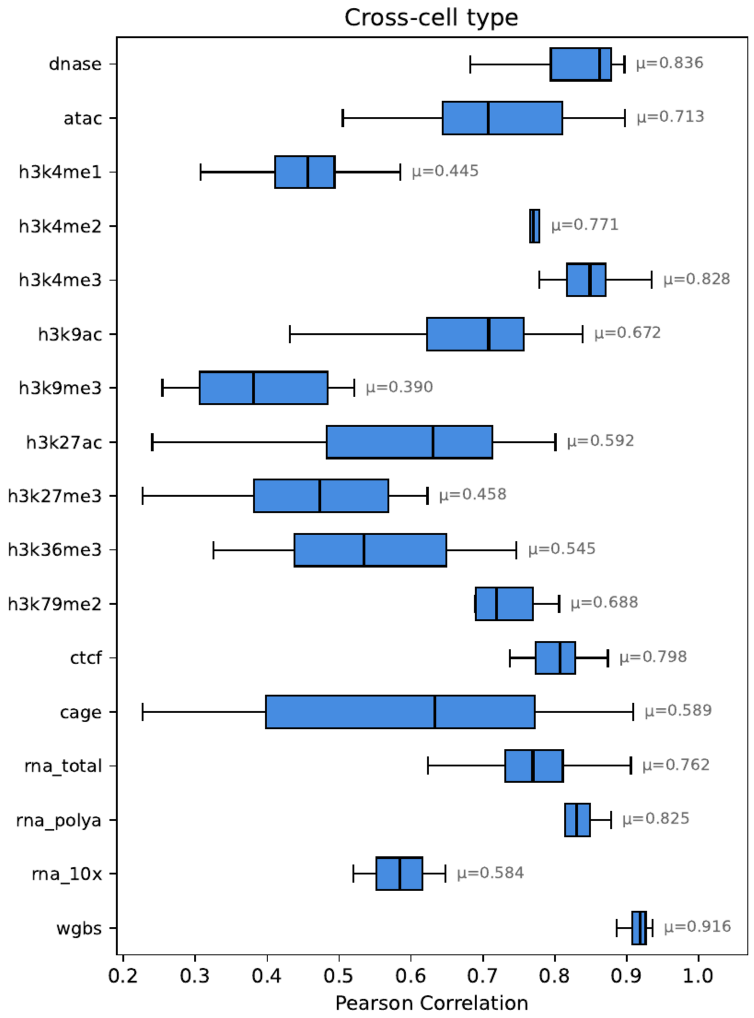

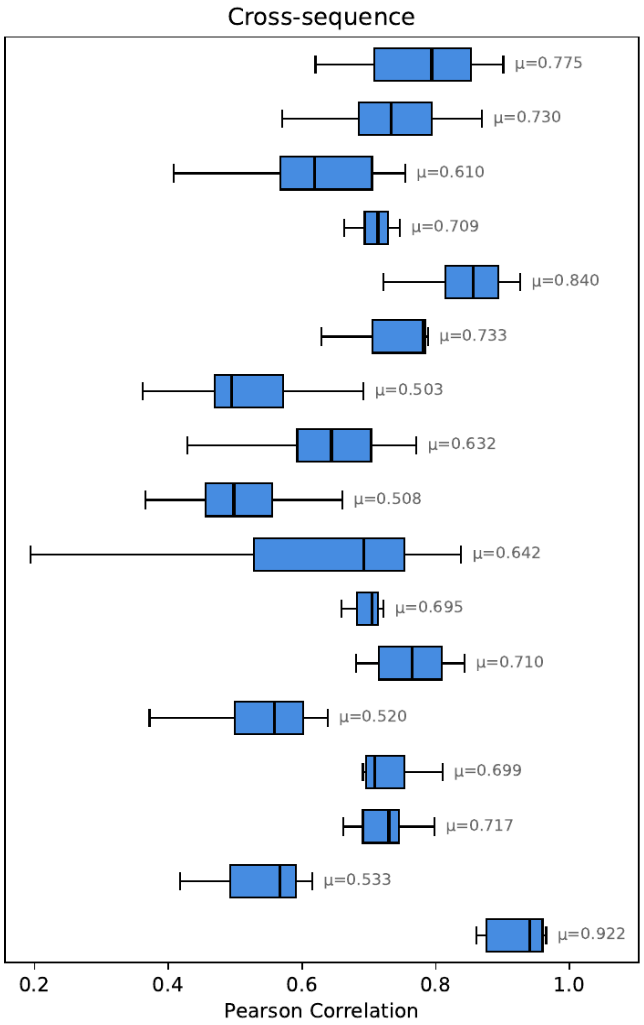

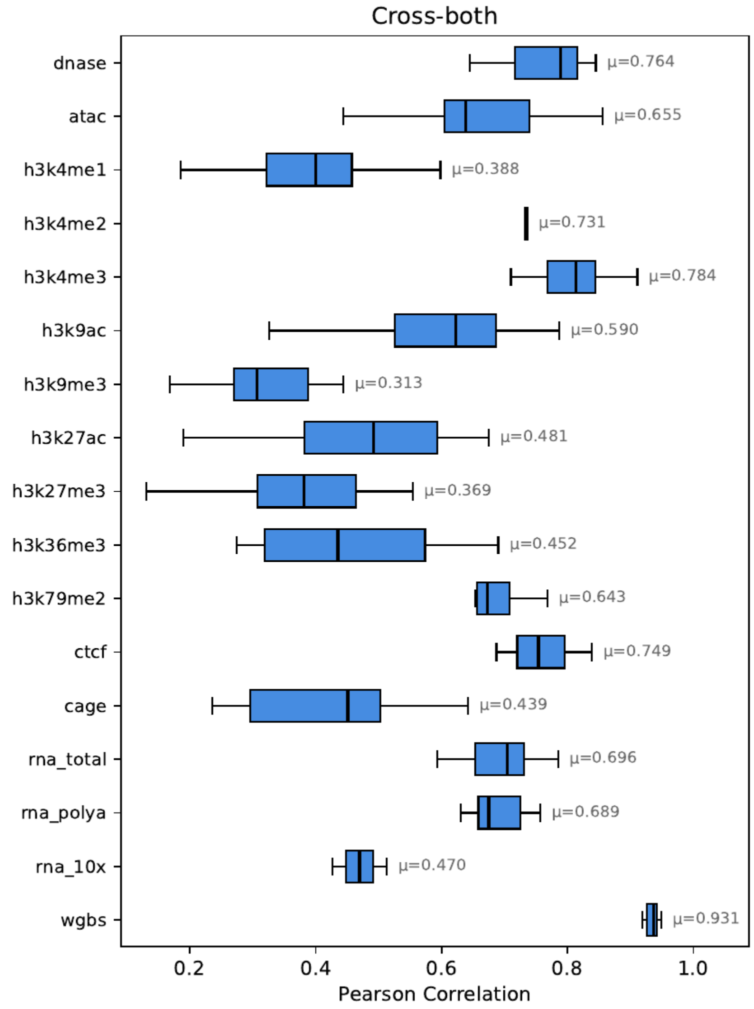

Figure S4. Detailed genomic coverage prediction performance across different tracks.
Boxplots showing model performance across different assays in cross-cell type, cross-sequence and cross-both settings. Correlations between predictions and ground truth data across genomic bins are reported, with the variance in the boxplots coming from different biological samples. Pearson's *r* values are reported with the mean values shown next to the boxplots. The boxes show the quartiles, and the whiskers extend until the furthest data point within 1.5 * interquartile range from both quartiles.

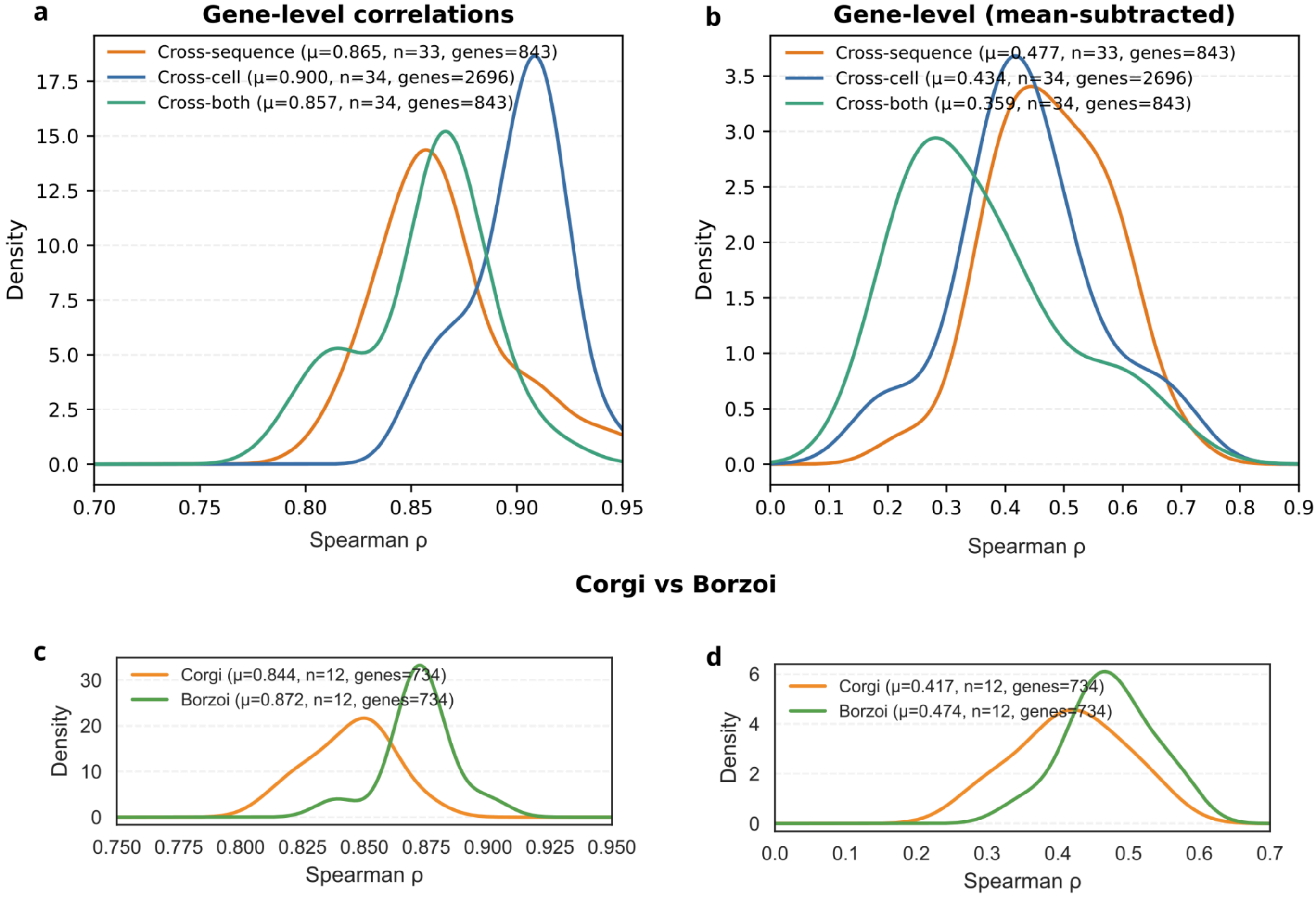

Figure S5. Gene-level spearman correlations

(a) shows distribution of gene-level Spearman's ρ coefficients between Corgi predictions and ground truth in the three benchmarks, and (b) shows Spearman's ρ coefficients between mean-subtracted Corgi predictions and mean-subtracted ground truth. (c) shows the distributions of gene-level Spearman's ρcoefficients for Corgi and Borzoi. This is a cross-sequence benchmark. (d) same data as (c) with mean-subtraction. Borzoi has a measurable edge over Corgi in this setting.

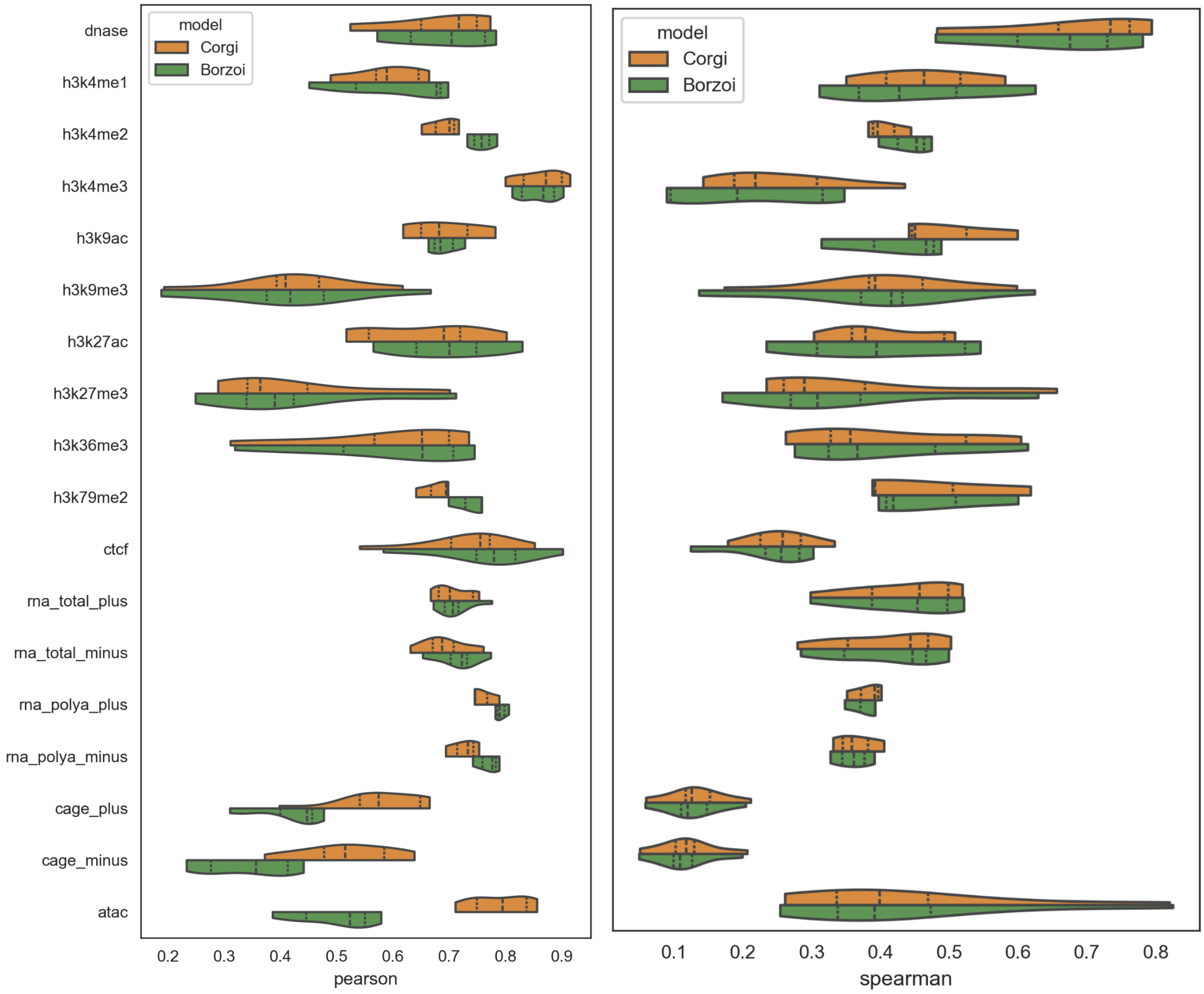

Figure S6. Detailed performance comparison of Corgi and Borzoi

Boxplots showing model performance across 30 matched tracks in a cross-sequence setting. Pearson's *r* and Spearman's ρ values are visualized, reflecting correlation between model predictions and ground truth data across genomic bins. Corgi and Borzoi show comparable predictive performance across tracks. Dashed lines show quartiles and the median.

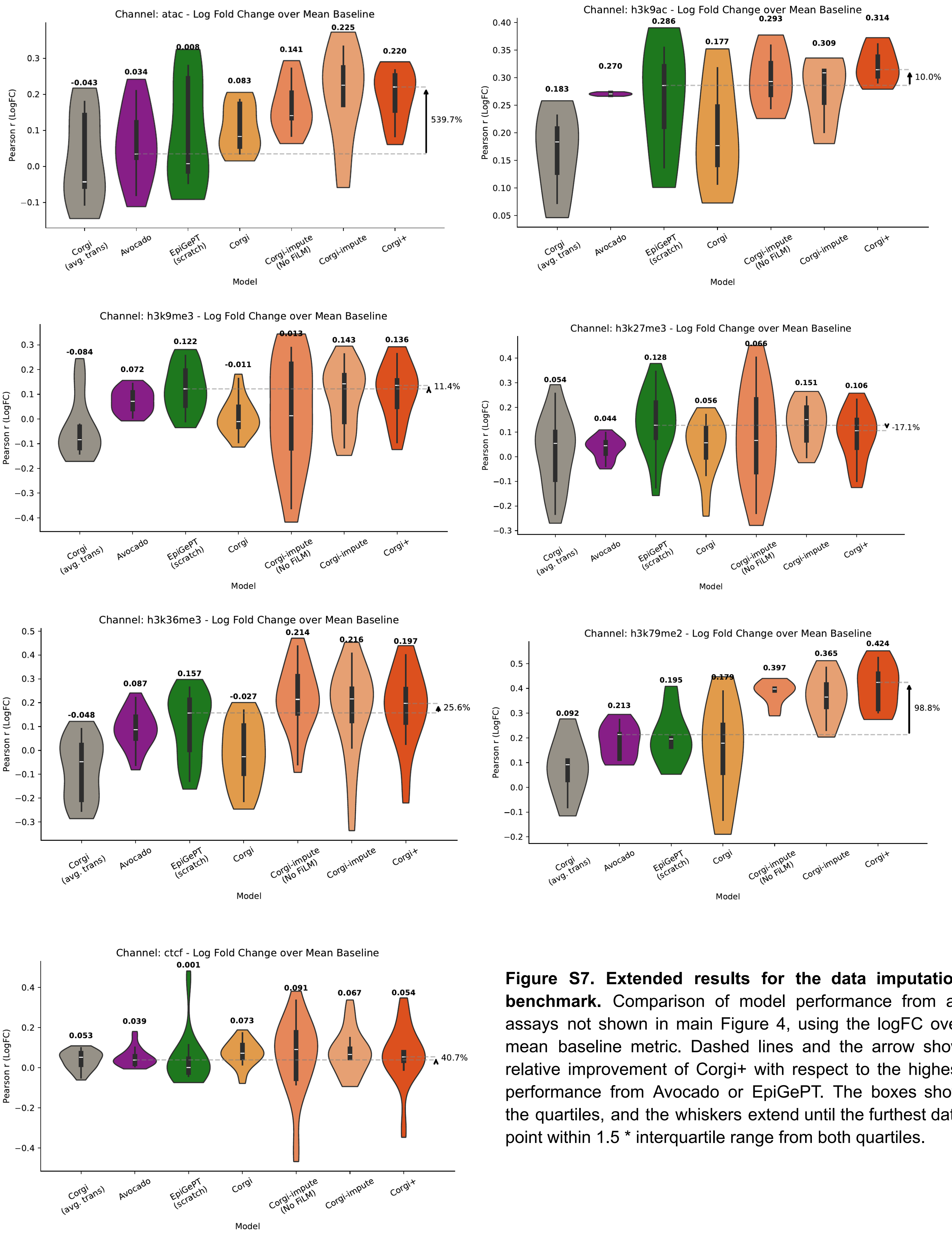

Figure S7. Extended results for the data imputation benchmark. Comparison of model performance from all assays not shown in main Figure 4, using the logFC over mean baseline metric. Dashed lines and the arrow show relative improvement of Corgi+ with respect to the highest performance from Avocado or EpiGePT. The boxes show the quartiles, and the whiskers extend until the furthest data point within 1.5 * interquartile range from both quartiles.

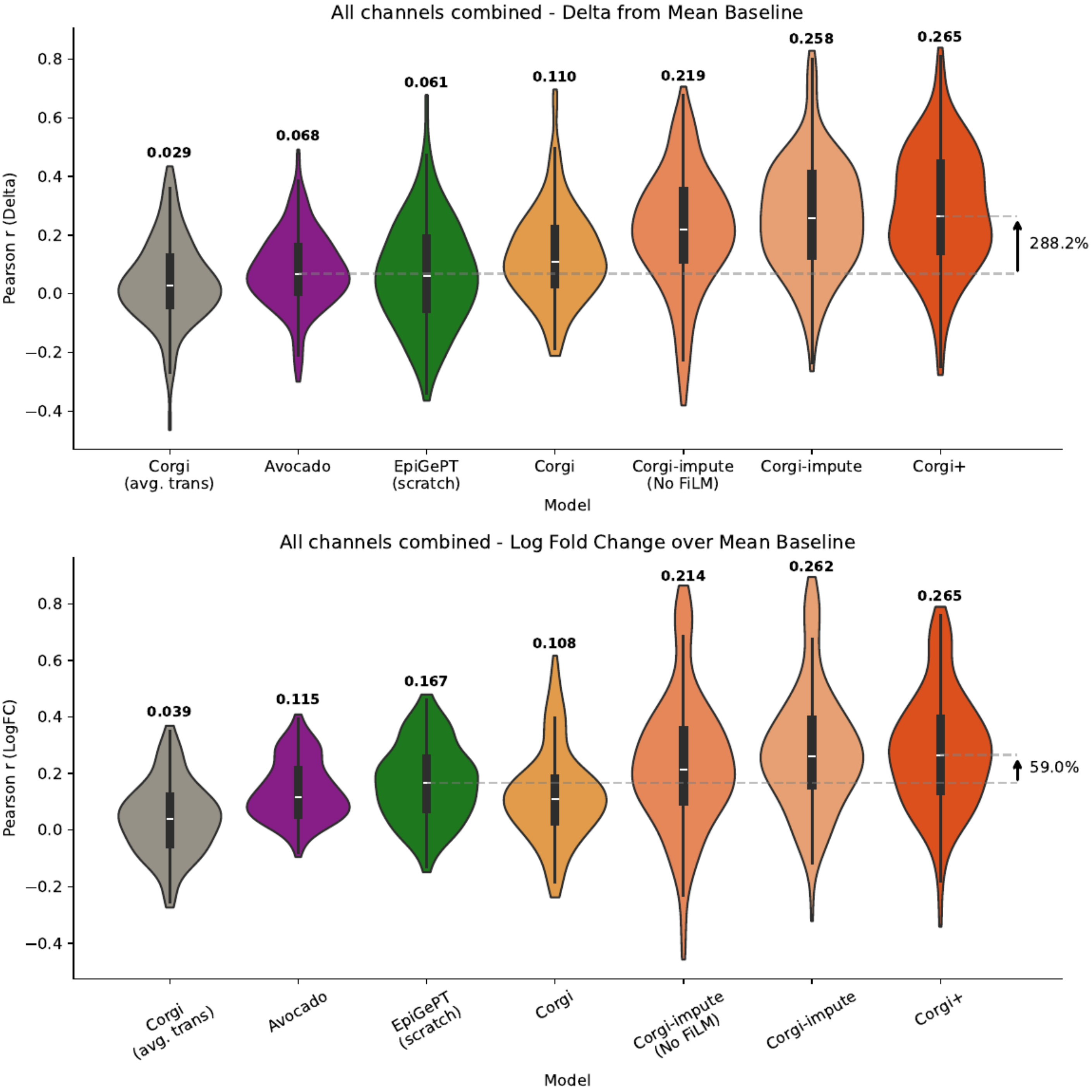

Figure S8. Comparison of data imputation performance when all assays are pooled.
Shows distributions of pearson *r* (delta) (top) and pearson *r* (logFC) (bottom) when all assays from FIgure 4c and Figure S7 are pooled together. Dashed lines and the arrow show relative improvement of Corgi+ with respect to the highest performance from Avocado or EpiGePT. The boxes show the quartiles, and the whiskers extend until the furthest data point within 1.5 * interquartile range from both quartiles.

a

GSEA using gene expression

iPSC

Term	Overlap	Genes	Adjusted P-value
Myc Targets V1	31/200	YWHAE, EIF4A1, HDAC2, SRSF1...	6.9e-37
Spliceosome	19/150	DDX5, SRSF1, HNRNPU, LSM4...	1.2e-19
Transit Amplifying (TA) Cell Intestinal Crypt Mouse	11/128	H2AZ1, RPS4X, PKM, KHSRP...	7.2e-09

Cardiac myocyte from RUES2

Term	Overlap	Genes	Adjusted P-value
Myc Targets V1	18/200	YWHAE, EIF4A1, CNBP, HNRNPU...	2.2e-16
Spliceosome	16/150	DDX5, RBM8A, HNRNPU, LSM4...	4.2e-15
ATRIAL SEPTAL DEFECT	4/11	CITED2, TBX20, GATA4, NKX2-5	1.0e-05

Liver (#276)

Term	Overlap	Genes	Adjusted P-value
Myc Targets V1	15/200	YWHAE, EIF4A1, CNBP, HNRNPU...	2.9e-12
Spliceosome	9/150	DDX5, HNRNPK, DDX39B, SNRNP70...	1.1e-05
Xenobiotic Metabolism	9/200	MTHFD1, ARG1, ID2, HNF4A...	1.5e-05

Liver (#277)

Term	Overlap	Genes	Adjusted P-value
Myc Targets V1	19/200	YWHAE, EIF4A1, XRCC6, CNBP...	1.1e-17
Hypoxia	11/200	GRHPR, ZFP36, PRDX5, UGP2...	1.0e-07
TNF-alpha Signaling via NF-kB	9/200	ZFP36, CEBPB, GADD45B, CEBPD...	7.4e-06

Neural progenitor from H9

Term	Overlap	Genes	Adjusted P-value
Myc Targets V1	31/200	YWHAE, EIF4A1, HDAC2, SRSF1...	6.9e-37
Spliceosome	22/150	DDX5, RBM8A, SF3B6, SRSF1...	1.6e-24
E2F Targets	15/200	H2AZ1, HELLS, XRCC6, SRSF1...	7.4e-13

Testis

Term	Overlap	Genes	Adjusted P-value
Myc Targets V1	20/200	YWHAE, EIF4A1, XRCC6, CNBP...	2.8e-19
Spliceosome	13/150	SF3B2, DDX5, HNRNPU, HNRNPK...	6.0e-11
RNA transport	9/186	EIF4A2, EIF4A1, DDX39B, PABPC4...	2.1e-05

b

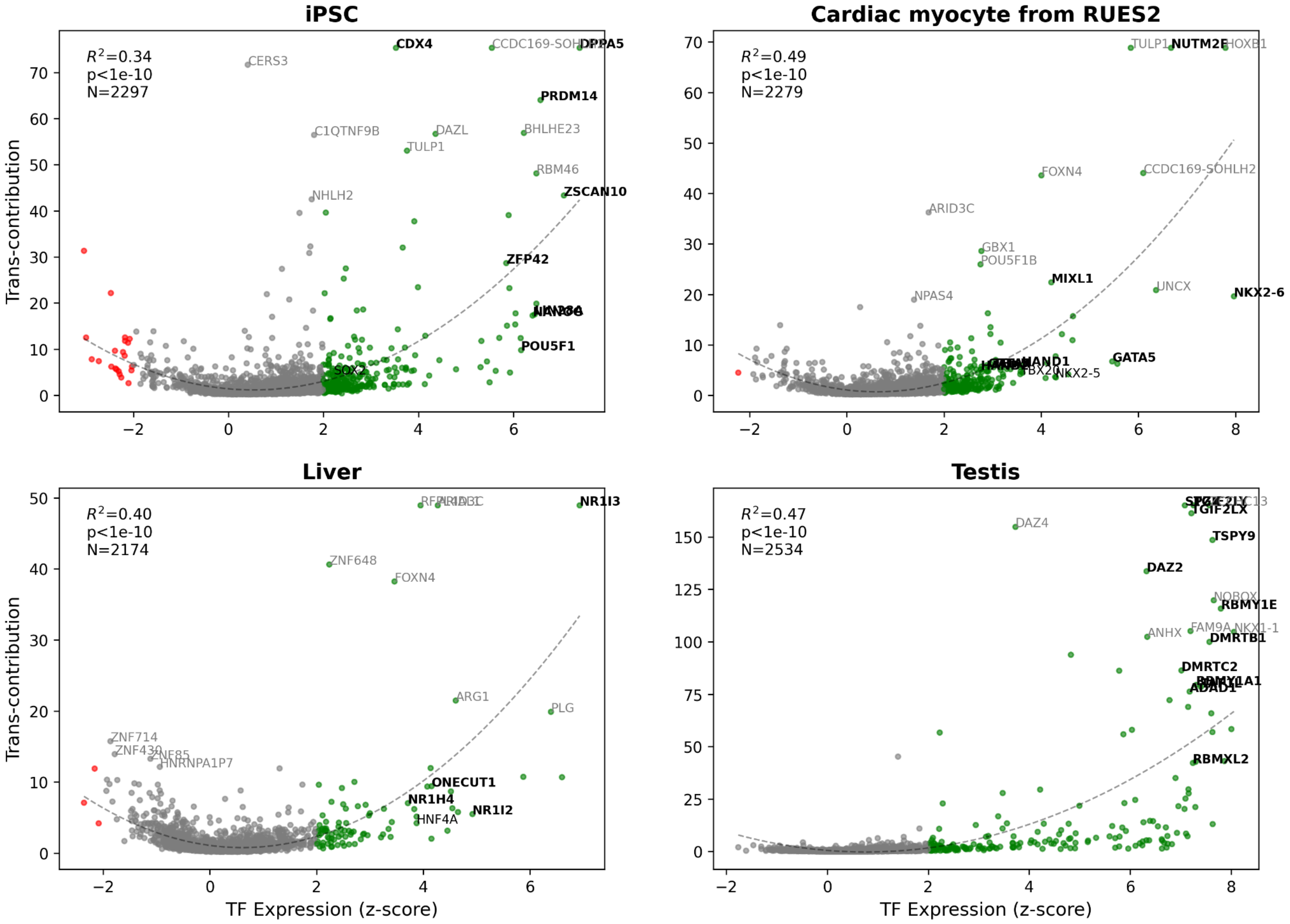

Figure S9. Sanity checks confirm accuracy of the *trans*-regulator causality analysis
(a) Gene set enrichment analysis results using gene expression values instead of *trans*-contributions. Contrary to Main Figure 5, using gene expression does not lead to the identification of key cell type-specific regulators or pathways. (b) Analysis from Figure 5d, after replacing *trans*-contribution with “*trans*-contribution divided by input”. Important regulators still have high scores, and significant correlations between the new readout and expression z-scores persist. Statistical analysis methods were the same as in main Figure 5.

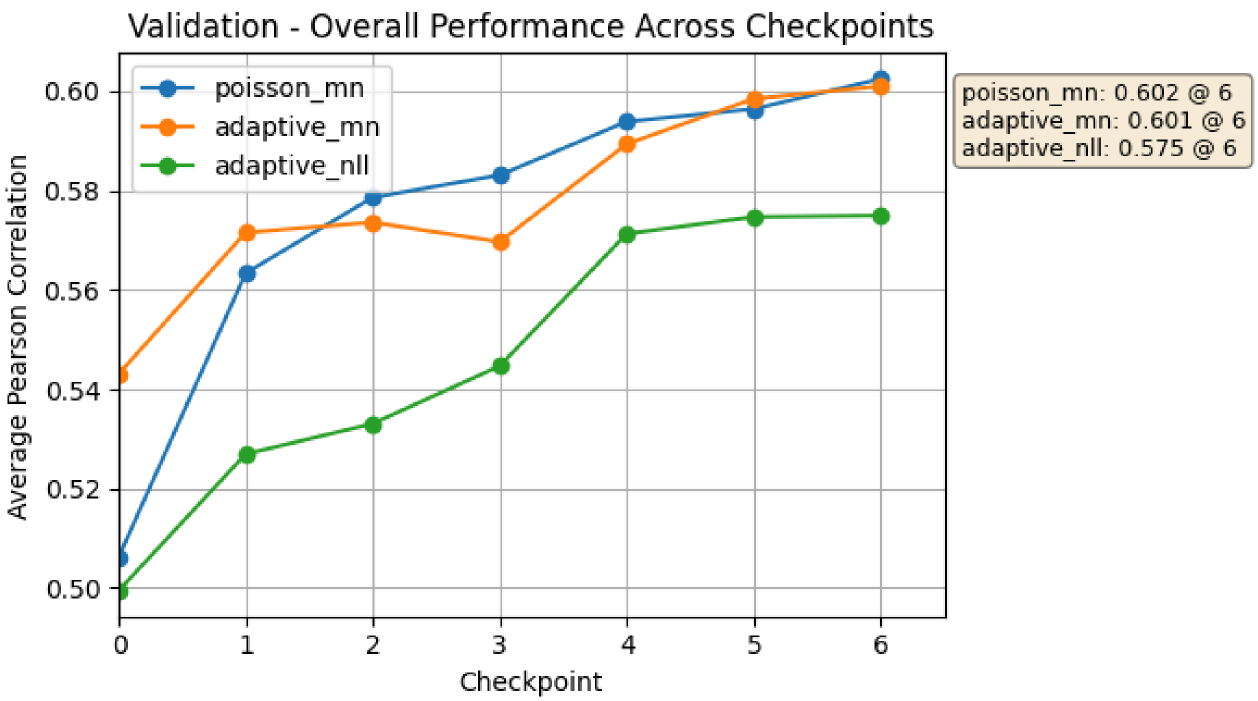

Figure S10. Validation performance across different model checkpoints

This plot shows the mean Pearson's *r* of all channels (y-axis) with respect to model training checkpoints. The colors represent different loss functions. Orange line is the adaptive poisson multinomial loss function used in Corgi, blue line is the poisson multinomial loss (similar to Borzoi) but with fixed scaling parameters for all output channels (e.g. losses for DNase and RNA are multiplied by 5, while CAGE is multiplied by 100). The fixed values were set empirically. The green line is a poisson negative log likelihood loss function that also has adaptive weights for output channels, but it does not include the decomposition of the loss into shape and total coverage terms. The scaled multinomial loss and the adaptive multinomial loss showed similar performance after the same training epochs. Eventually the latter was selected due to its principled approach as opposed to fixing arbitrary weights empirically. Without using any kind of scaling, performance of certain tracks were diminished (e.g. CAGE-seq).

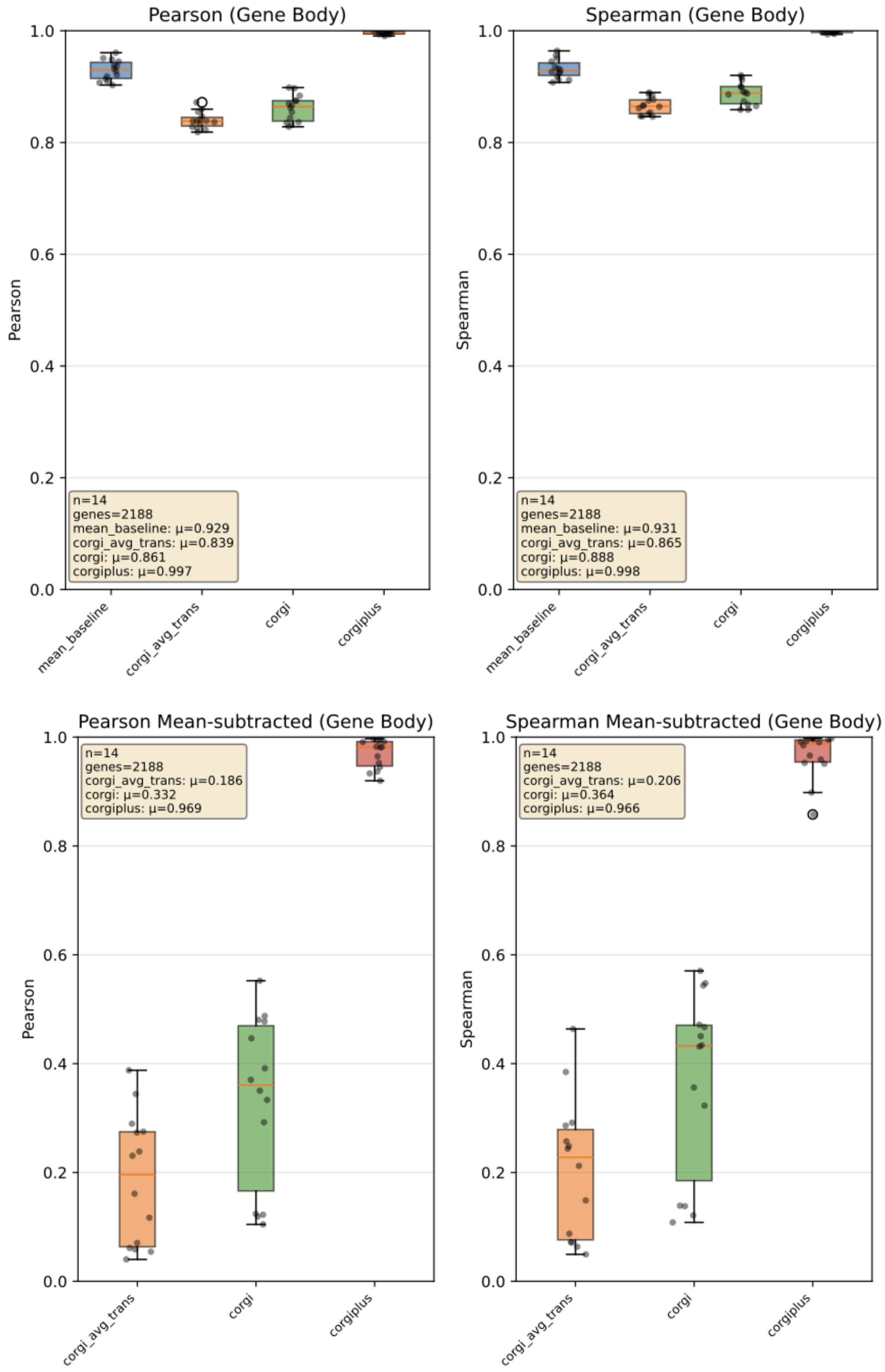

Figure S11. Gene expression predictions in the imputation dataset

The first row of subfigures show gene-level pearson and spearman correlations in the imputation dataset. Using the baseline gene expression levels yields a high correlation. However, the baseline cannot capture cell type-specific differences in gene expression. The bottom row shows mean-subtracted correlations, showing that Corgi with correct *trans*-regulator levels is outperforming *Corgi (avg. trans).* Corgi+ has perfect accuracy, which acts as a positive control here, since it has RNA-seq data in its input. The boxes show the quartiles, and the whiskers extend until the furthest data point within 1.5 * interquartile range from both quartiles.

Supplementary Table Legends

Supplementary Table 1. The Corgi dataset is shown, along with source consortium, assigned cluster, training/validation/test split, and numbers of tracks for different functional genomics experiments.

Supplementary Table 2. List of gene names defined as *trans*-regulatory factors.

Supplementary Table 3. List of genomic regions in the Corgi dataset. Fold3 was reserved for testing, and fold 4 was reserved for validation. The rest of the folds were used for training the Corgi model.

Supplementary Table 4. Matched tracks between Corgi and Borzoi. Each row shows one track from the Borzoi dataset that is matched to one sample in the Corgi dataset. Channel_id represents the index of the channel within the Corgi dataset, while borzoi_track_id represents the track index within the Borzoi dataset.

Supplementary Table 5. List of ENCODE experiments used in this work, along with which samples and replicates were used, ENCODE audit information, and download links.

Supplementary Table 6. List of FANTOM experiments used in this work. The encode_id column represents the sample index numbers for samples which were matched with ENCODE samples.
