# Supplementary material for: Context-aware sequence-to-function model of human gene regulation
Source: Nat Commun. 2026 Jul 14;17:6200. doi: 10.1038/s41467-026-75527-2 (PMC13370016; doi:10.1038/s41467-026-75527-2)
Supplement: Supplementary file 2 — Description of Additional Supplementary Files [file 41467_2026_75527_MOESM2_ESM.pdf]

Description of Additional Supplementary Files

Supplementary Data 1. The Corgi dataset is shown, along with source consortium, assigned cluster, training/validation/test split, and numbers of tracks for different functional genomics experiments.

Supplementary Data 2. List of gene names defined as *trans*-regulatory factors.

Supplementary Data 3. List of genomic regions in the Corgi dataset. Fold3 was reserved for testing, and fold 4 was reserved for validation. The rest of the folds were used for training the Corgi model.

Supplementary Data 4. Matched tracks between Corgi and Borzoi. Each row shows one track from the Borzoi dataset that is matched to one sample in the Corgi dataset. Channel_id represents the index of the channel within the Corgi dataset, while borzoi_track_id represents the track index within the Borzoi dataset.

Supplementary Data 5. List of ENCODE experiments used in this work, along with which samples and replicates were used, ENCODE audit information, and download links.

Supplementary Data 6. List of FANTOM experiments used in this work. The encode_id column represents the sample index numbers for samples which were matched with ENCODE samples.
