# Supplementary material for: Context-aware sequence-to-function model of human gene regulation
Source: Nat Commun. 2026 Jul 14;17:6200. doi: 10.1038/s41467-026-75527-2 (PMC13370016; doi:10.1038/s41467-026-75527-2)
Supplement: Supplementary file 10 — Transparent Peer Review file [file 41467_2026_75527_MOESM10_ESM.pdf]

nature portfolio

Peer Review File

Context-aware sequence-to-function model of human gene regulation

Corresponding Author: Professor Martin Vingron

Parts of this Peer Review File have been redacted as indicated to remove third-party material.

This file contains all reviewer reports in order by version, followed by all author rebuttals in order by version.

Version 0:

Reviewer comments:

Reviewer #1

(Remarks to the Author)
Ekin Deniz Aksu et al. introduced Corgi (Context-aware Regulatory Genomics Inference), a novel context-aware sequence-to-function model designed to predict human regulatory genomic features, including gene expression, chromatin accessibility, and various epigenetic marks. Remarkably, the model achieves high predictive accuracy even in previously unseen cell types. Corgi integrates multiple architectural components: convolutional layers to extract local cis-acting sequence features, a multilayer perceptron (MLP) to model trans-acting regulatory information, and FiLM (feature-wise linear modulation) layers to affine-transform and fuse these two sources of information. A Transformer module is further incorporated to capture long-range regulatory interactions. Through systematic benchmarking against EpiGePT and Borzoi under three evaluation strategies, the authors demonstrate the strong performance and robustness of Corgi. Below, I provide several suggestions that may help further improve the clarity and overall quality of the manuscript.
1. In the main text, the authors highlight that they address a key challenge in context-aware sequence-to-function modeling —namely, the difficulty of integrating DNA sequences with context vectors due to their differing dimensionalities. However, existing methods such as EpiGePT, EPCOT, and EnformerCelltyping have already proposed effective strategies for fusing sequence information with contextual embeddings. Thus, given the current state of the field, this issue may no longer represent a major bottleneck. I suggest that the authors reconsider how this contribution is framed, or more clearly articulate the specific limitations of prior integration approaches that Corgi uniquely overcomes.
2. It is unclear what the variable n specifically refers to in Figure 2. Providing a clearer explanation would improve the readability and interpretability of the manuscript.
3. Several methods have been developed to predict cell type-specific epigenomic landscapes. In addition to EpiGePT, more recent approaches such as EPCOT and EnformerCelltyping are also representative in this domain. I recommend including these methods as additional baselines in the benchmarking experiments to more comprehensively demonstrate the advantages of Corgi. Moreover, the manuscript appears to understate the performance of EpiGePT and Borzoi. According to the original EpiGePT publication, its cross-cell type, cross-sequence, and joint cross-generalization performances are reported as 0.787, 0.504, and 0.515, respectively. As one of the leading sequence-only models, Borzoi should also exhibit cross-sequence performance close to its reported values. Although differences in data processing pipelines may cause some performance degradation, such substantial declines seem unlikely. Therefore, I suggest that the authors clarify the evaluation setup, data preprocessing steps, or experimental conditions to avoid potential misinterpretation of existing methods' performance.
4. The presentation of results in the section "Corgi can impute epigenomic tracks in a cross–cell type setting" is not sufficiently clear. The previous section has already demonstrated Corgi's ability to generalize across cell types, so the manuscript should more explicitly articulate the conceptual and methodological distinction between the two sections. I suggest reorganizing this part to clarify its standalone contribution or how it differs from the preceding analyses. In addition, including brief concluding paragraphs at the end of each major section would improve the overall coherence and readability of the manuscript, while further highlighting Corgi's innovations and advantages.
5. In the section "Corgi understands cell type-specific regulation by learning biologically meaningful trans-regulators," the authors state that "genomic sequencing data is highly correlated across different contexts, and often simple baselines can yield good prediction accuracies." If biological signals are indeed highly correlated across contexts and simple baseline models can already perform well, it remains unclear why such a complex architecture is necessary to model context-dependent sequence-function relationships. The authors should clarify the necessity of this model complexity and the

biological or predictive benefits it provides. Moreover, the way the results are presented in this section does not clearly convey the authors' main findings, which hampers the interpretability of this analysis. I suggest more clearly articulating the significance of these findings and how they support the conclusion that Corgi effectively learns cell type–specific regulatory mechanisms.
6. In the section "Corgi can identify upregulated trans-regulators in a zero-shot manner in held-out tissues," the authors claim that Corgi can identify upregulated trans-regulators in tissues that were never seen during training. However, the manuscript does not clearly explain the mechanism by which the model achieves zero-shot identification—for example, how Corgi infers regulatory factors from previously unseen context vectors, what signals or internal representations enable this capability, and how this process relates to the model's supervision strategy. I suggest that the authors more explicitly describe Corgi's inference pipeline under zero-shot settings and articulate the theoretical or architectural basis for this capability. Providing such clarification would improve the clarity and credibility of this section and strengthen the overall persuasiveness of the manuscript.
7. In the discussion section, the authors note that Corgi is capable of inferring missing experimental assays using RNA-seq data. However, the results section provides only limited evidence supporting this key capability. Given that such cross-modal inference represents one of Corgi's core innovations, I recommend including more systematic and comprehensive experiments and analyses in the results section to highlight Corgi's ability to use RNA-seq data to impute epigenomic features. Doing so would more fully demonstrate the model's unique contributions and practical potential.

(Remarks on code availability)

Reviewer #2

(Remarks to the Author)
Summary:

Aksu et al. develop a deep learning model called Corgi, which is capable of predicting transcriptomic and epigenomic coverage tracks in unseen cell types and tissues. The model is largely based on the Borzoi/Enformer architecture, but has an additional MLP network that receives trans-regulatory expression values as an additional input vector, which allows the authors to predict what coverage tracks should look like in new contexts by passing an appropriate trans-regulatory expression vector to the model. The authors evaluate their model at the tasks of predicting gene expression and epigenomic activity in three settings: (1) held-out genomic sequences, (2) held-out tissues or cell types, and (3) held-out sequences and cell types. They demonstrate competitive performance to state-of-the-art methods while introducing the new capability of predicting coverage in unseen cell types.

Significance, novelty and utility:

An interesting study that introduces a new approach for modeling sequence-to-function tasks without the multi-tasking paradigm. As noted by the authors, their approach is conceptually similar to methods like scooby, but with important differences (inherently interpretable input space consisting of trans-regulatory expression values as opposed to a low-dimensional embedding, and applied to bulk- and cell type data).

I have a number of concerns and comments detailed below. The largest concern is already discussed by the authors: the apparent inability of the model to causally predict differences in coverage tracks under slight perturbation of the trans-regulatory context. This is something that the scooby paper actually demonstrated somewhat successfully (Fig. 3 of https://www.nature.com/articles/s41592-025-02854-5).

Comments and suggested improvements:

Major:

1. I am not fully convinced the Corgi model truly uses the trans-regulator input vector in a biologically meaningful and causal way when making its predictions. As the authors describe in the discussion, they fail to observe expected predicted changes in coverage tracks when increasing or decreasing the input expression value of a relevant trans-regulator. Ideally, I'd like to see an analysis similar in concept to the scooby paper (Fig. 3), where its clear that the model's predictions (and cis-regulatory contribution scores of a given TF motif) increase/decreases as a function of the trans-regulator concentration. Otherwise, it is hard to believe that these features are used causally by the model, as opposed to Corgi learning an arbitrary mapping from these vectors (as dictionary identifiers of sorts). At the very least, I'd like to see a variant effect prediction analysis on fine-mapped eQTLs, where Corgi is successfully able to predict beta coefficients of held-out tissues (e.g., liver).

2. In the analysis presented in Figure 4b, I am hesitant to believe that the improvement over the mean baseline is statistically significant. Can the authors show this? Also, is there a reason why Corgi's performance improvement over Avocado in Figure 4a (or over Baseline in 4b) only looks significant for RAMPAGE? On a similar note, how well can Corgi predict gene expression or chromatin accessibility of held-out sequences for very cell-type specific loci (as opposed to cell type-agnostic, house-keeping genes, where the mean baseline is sufficient)?

Minor:

3. In Figure 3c, it would be prudent to compare the two models' on bin-level Pearson R metrics after (un-)transforming their predictions to the same scale. Additionally, Corgi is at 64 bp resolution while Borzoi is at 32 bp, and I would suggest average-pooling Borzoi's predictions to 64 bp before comparing, for fairness.

4. It would be good to compare the mean-subtracted performance metrics in Figure 5a and 5b to Borzoi's (or other SOTA models') performance.

(Remarks on code availability)
The code is available on github and documented.

Reviewer #3

(Remarks to the Author)
Summary:
The authors present a deep learning genomic sequence-to-function model, "Corgi," that includes context-specific expression levels of trans regulators as an auxiliary input with the goal of generalizing to unseen cell types. Strong performance is reported on the most difficult task of predicting expression of held-out genes in held-out cell types. Comparisons to the state-of-the-art model, Borzoi, are mixed.

Assessment: Overall, the Corgi strategy is a promising direction and a good application of feature-wise linear modulation (FiLM). However, it is not clear how performance improvements are achieved, which is important for evaluating how much trans regulator features contribute to predicting gene expression. To address this, the manuscript would benefit from additional baselines (e.g., mean gene expression in the training set) and evaluations. A few inconsistencies and likely influential filtering decisions are also noted.

General Comments:
Transcriptomics data were "harmonized" across assays by quantile normalization, log transformation and batch correction. As shown in the example in supplementary figure S3 (c)-(d), this results in high correlation between CAGE and RNA-seq from the same tissue. Since these assays measure different aspects of transcription, more justification is needed to support the assumption that the two assays "should" have a higher correlation than is observed in the raw data, and that harmonizing the two would be superior to alternative approaches (e.g. including both CAGE-seq and RNA-seq expression levels as distinct elements of the trans-regulator expression vector).

The authors go to great lengths to avoid leakage between the training and test sets, which is admirable. But these procedures greatly reduce the size of the test set (~4-fold), and likely biases it as well. We have several suggestions related to this:
1. Corgi is trained on the same genomic sequences and train-validation-test split as Borzoi, with the caveat that all windows that include a trans-regulator gene are excluded (to prevent data leakage from the context vector). While a sound approach, this reduces the number of 524,288-bp windows from 6,888 to only 1,437. Please acknowledge this clearly in the main text and discuss why this is a reasonable trade-off.
2. When bin-level correlations are computed, it appears that only 637 windows were used (Benchmarking section of Methods). Please clarify how the 637 were selected and justify this decision in the manuscript.
3. Excluding trans regulator genes (and their neighbors) from the test set may have eliminated many of the genes that exhibit the most context-specific patterns of gene expression. We recommend evaluating the variability in expression across cell types of the excluded versus retained genes to assess if the filter biased the test set towards (or away) from broadly expressed genes.

It is appealing to conclude that Corgi's trans-regulatory context vector is being used to enhance the performance of the model. However, the results do not explicitly show this. To clarify the value added from the trans regulator inputs, we recommend performing additional comparisons in the "cross-cell type" evaluation. First, the correlation between observed gene expression in each test cell type should be compared to two simple baselines: 1) the average observed expression among all training cell types, and 2) Corgi's predictions given a context vector composed of the average trans-regulator expression level among all training cell types. These comparisons will clarify if Corgi is doing more than predicting the typical expression level of each gene. In addition, it is recommended that Corgi-like models be trained using only sequence inputs and using only trans regulator inputs. Then these models should be compared to the Corgi model (trained using both inputs) across the various evaluation benchmarks in the manuscript. This would help to quantify, all other things being equal, the contributions of sequence and trans regulator expression.

The novelty of Corgi should be tempered slightly in the text in recognition of existing methods for various components of Corgi, such as Avocado for imputation, Borzoi (and related models) for multi-task sequence-to-activity predictions, and EpiGePT (and related models) for epigenetic track predictions based on sequence plus trans regulator expression. The authors cite and benchmark against these methods, which is great. Our recommendation is simply to talk about these existing strategies in the introduction and carefully delineate what is novel in Corgi (e.g., combining aspects of these different methods, using FiLM).

Specific Comments:
Preventing leakage: Evaluating a model's generalization capability in held-out cell types is difficult because of the highly correlated patterns of gene expression across cell types. The authors devise a clustering-based strategy for preventing data

leakage of this type by applying agglomerative clustering to 580 samples according to their gene expression levels, then removing clusters from the training set until a desired number of validation and testing clusters was reached. This seems like a good approach. But (1) insufficient detail is provided on the exact clustering methods used, as well as how clusters were selected for inclusion into validation or test sets, and (2) performance on the “easy-test” set (constructed by randomly sampling clusters from the training set) is not reported. Describing the clustering method and including results for the “easy-test” set would be very helpful for contextualizing the presented performance results.

Harmonization: In the main text, it is implied, but not explicitly stated, that the harmonization procedure was applied only to trans regulator expression levels. This should be clarified.

Figure 2d: Corgi’s performance on “cross-both” matches or exceeds its performance on “cross-sequence” gene expression predictions, with both tasks reporting extraordinarily high Pearson’s correlations (>0.9) that exceed the performance of “cross-cell” predictions. It is surprising that “cross-both” would outperform “cross-cell”, since both tasks involve generalizing to a held-out cell type with “cross-both” presenting the additional challenge of predicting on genes not seen during training. Is there an error here?

Figure 3: EpiGePT’s performance is consistently shown to be dramatically inferior (panels (a)-(b)) and its coverage track predictions appear very poor qualitatively (panel (e)). Given the stated difficulty in getting EpiGePT’s code to run, this suggests that perhaps parameter optimization did not converge. This analysis needs to be revisited/repeated.

Figure 3: Corgi’s “Cross-sequence” performance appears comparable to Borzoi’s across many assays, with notable exceptions of CAGE and ATAC (panel (c)). An explanation of this discrepancy would be useful. Could it be due to differences in the models’ target resolution (64 bp vs. 32 bp)?

It is our policy to sign reviews: Alexander Floren & Katie Pollard

(Remarks on code availability)
Model weights and code are available. There is no installation info, tutorial, etc. So, it would be challenging for some one else to use the code. The configuration file points to specific data files in the repo, so would need to be updated if a user wanted to train their own model. The submission script is specific to SLURM. These are probably OK, but should be explained (e.g., in an added tutorial).

Reviewer #4

(Remarks to the Author)
I co-reviewed this manuscript with one of the reviewers who provided the listed reports. This is part of the Nature Communications initiative to facilitate training in peer review and to provide appropriate recognition for Early Career Researchers who co-review manuscripts.

(Remarks on code availability)

Version 1:

Reviewer comments:

Reviewer #1

(Remarks to the Author)
The revised manuscript has substantially improved in overall quality, and the authors’ careful efforts in addressing the reviewers’ comments are appreciated. Despite these improvements, the current benchmarking remains relatively limited, as the study only compares Corgi with EpiGePT and Borzoi. I recommend expanding the comparison to include additional relevant methods in order to strengthen the credibility of the evaluation.
Specific suggestions are as follows:
1. Include comparisons with EPCOT and EnformerCelltyping
Although Corgi uses RNA-seq expression data while EPCOT and EnformerCelltyping rely on different modalities of cell-state information, such as chromatin accessibility, including these methods in the comparison would still provide valuable context. Evaluating models across different conditioning modalities could help assess performance from complementary perspectives and provide a more informative background for understanding the potential advantages of Corgi.
2. Evaluate these baseline models under the cross–cell-type setting
In their response, the authors note that EnformerCelltyping requires local chromatin accessibility information and therefore cannot be applied to evaluation settings where sequence information is completely held out (e.g., cross-sequence or cross-both). This limitation is understandable. However, both EnformerCelltyping and EPCOT can still be evaluated under the cross–cell-type setting, which is also the evaluation scenario adopted in the current study. Therefore, including these methods as baselines in this setting should be technically feasible.
Overall, incorporating these additional comparisons would strengthen the benchmarking framework and provide a clearer reference for understanding the relative advantages of different types of cell-state information (e.g., RNA expression versus chromatin accessibility) in sequence-to-function prediction tasks.

This review has taken into consideration the comments raised by Reviewer #2, and the points discussed in my report were intended to address both my own evaluation and the issues highlighted by Reviewer #2.

(Remarks on code availability)

Reviewer #3

(Remarks to the Author)
The authors conducted analyses to address many of our comments. Responses for R3.4, R3.6, R3.9, and 3.10 are sufficient. However, in other cases the specific reviewer question was not directly answered or the answer was only shared in the response document and was not used to improve the clarify of the manuscript. Corgi is an exciting new modeling framework, but additional results and text details are needed in order to clarify it performance, especially on gene expression prediction tasks. We therefore make the following suggestions for further revision, organized using the numbering system from the authors' response document.

R3.1. We acknowledge the reasons given for CAGE + RNA data "harmonization" but reiterate that this justification should be included in the main text.

R3.2. Rather than (or in addition to) the number of windows, we ask the authors to state the amount of unique (i.e. non-overlapping) sequence (in bp), as well as the number of genes present in the validation and test sets after removal of trans-regulator gene bodies and re-tiling of the remaining sequences. This is important because windows were redefined, and they overlap, making it impossible to infer these two key numbers from the window count. These two statistics should be reported and compared against the equivalent statistics for Borzoi's validation and test sets. The Methods paragraph edited in response to the initial comment is a reasonable place to put this information.

R3.3. The new baselines outlined in Figure 4a are very interesting, and they are important for evaluating imputation performance (per other reviewer comments). However, our original comment suggested something different. We ask about a baseline using mean gene expression plus sequence and no other inputs as a comparator for gene expression prediction tasks—not for imputation of chromatin marks. It is recommended that this evaluation is added, because it will enable the authors to illustrate the benefit of including trans regulator expression.

Additionally, we recommend rigorously defining "mean baseline"; the description "mean training baseline signal from all Corgi tracks" is not sufficiently detailed to tell what was actually done (are different assays averaged or all cell types per assay type or something else, and is this done per bin for a given window or is it a genome-wide average?). Please clarify what tracks go into the mean. An example might be helpful, such as "for example, to predict H3K27ac for a given window in a specific cell type, we use the mean value of observed H3K27ac tracks per bin in this window across all cell types in our catalog".

R3.5. The authors clarified their clustering methods for selecting cell types to hold out. This is clear now.

Regarding the easy-test set, the authors reported (in the response document) gene-level Pearson correlation distributions for three expression prediction tasks. They performed a statistical test (not sure which one, please add this detail) comparing the cross-sequence and cross-both Pearson correlation distributions over easy-test examples, and they argue that the lack of significant difference between these two means that similarity to training data is not driving performance. These results are useful but do not directly address the original recommendation. Perhaps we were unclear; we did not specify which metrics we wanted to see. To clarify, our suggestion was to compare gene-level Pearson and Spearman correlation distributions for the easy-test set versus the test set and the versus the training set on the same tasks: cross-sequence, cross-cell type, and cross-both.

Further, the Pearson correlations reported for the "easy-test" set are somewhat lower than expected based on other results in the manuscript. Is it possible that the figure used the "mean-subtracted" expression values rather than the raw values? It would be good to clarify this.

R3.7. The results from Figure 2e-f should be contextualized against Borzoi's performance as in the new supplementary Figure 5, but using Pearson's correlation (for consistency with other figures and publications). Corgi's "mean-subtracted" cross-sequence performance lags substantially behind that of Borzoi, although Borzoi of course lacks the ability to generalize to new cell types. This an important trade-off and should not be relegated to the supplement.

The authors note that they switched their method of calculating gene-level expression from aggregating over exons to aggregating over entire gene bodies. Why was this done? This change makes it harder to compare against existing methods (like Borzoi), and could also influence the reported performance. We recommend showing results for both aggregation approaches or providing a strong justification for the switch.

It would also be helpful to display the underlying data points instead of just showing densities in Fig. 2e-f.

Finally, regarding transformation of the RNA-seq data, it is important to specify what log base and what pseudocount were used. The example scatterplot (Fig. 2h) shows a large gap between data points with measured expression of 0 and the next

lowest value, with measured expression of ~4. In our experience, the choice of pseudocount can affect the resulting Pearson correlation in situations like this.

R3.8. Given that the changes made to the EpiGePT pipeline “did not visibly change its predictions”, could there be remaining bugs?

It is our policy to sign reviews: Alexander Floren & Katie Pollard

(Remarks on code availability)
The code was modified to have an option to run via SLURM or on a local GPU. This is a welcome addition.

Reviewer #4

(Remarks to the Author)
I co-reviewed this manuscript with one of the reviewers who provided the listed reports. This is part of the Nature Communications initiative to facilitate training in peer review and to provide appropriate recognition for Early Career Researchers who co-review manuscripts.

(Remarks on code availability)

Version 2:

Reviewer comments:

Reviewer #3

(Remarks to the Author)
The authors conducted analyses and added text to address our comments. The revision is much improved with these details added. We have two minor but important suggestions:

R3.5 from prior number system: To contextualize the new results for cross-sequence and cross-both (easy-test) performance, it is important to report alongside these the pooled all tracks cross-both (test) performance.

General: With respect to imputation, it is appropriate to say that Corgi/Corgi+ is cutting-edge. But with respect to gene expression prediction, performance is not notably better than sequence-only models. Hence, we recommend toning down phrases such as “state-of-the-art performance” and “experimental-level accuracy” when referencing gene expression prediction.

General: We agree that predicting gene expression with RNA as a model input is not useful from the perspective of the predictions themselves. But such a model might be useful for learning gene regulatory mechanisms, in this case cell type specific gene expression. The authors may want to speculate about this in the discussion or results.

It is our policy to sign reviews: Alexander Floren & Katie Pollard

(Remarks on code availability)
No new comments on the code.

Reviewer #4

(Remarks to the Author)
I co-reviewed this manuscript with one of the reviewers who provided the listed reports. This is part of the Nature Communications initiative to facilitate training in peer review and to provide appropriate recognition for Early Career Researchers who co-review manuscripts.

(Remarks on code availability)

Open Access This Peer Review File is licensed under a Creative Commons Attribution 4.0 International License, which permits use, sharing, adaptation, distribution and reproduction in any medium or format, as long as you give appropriate credit to the original author(s) and the source, provide a link to the Creative Commons license, and indicate if changes were made.
In cases where reviewers are anonymous, credit should be given to 'Anonymous Referee' and the source.
The images or other third party material in this Peer Review File are included in the article's Creative Commons license, unless indicated otherwise in a credit line to the material. If material is not included in the article's Creative Commons license and your intended use is not permitted by statutory regulation or exceeds the permitted use, you will need to obtain permission directly from the copyright holder.
To view a copy of this license, visit https://creativecommons.org/licenses/by/4.0/

Response to Reviewers

Context-aware sequence-to-function model of human gene regulation
Ekin Deniz Aksu and Martin Vingron

We thank the reviewers for their evaluation of our manuscript and their valuable feedback. We identified three major areas of concern in the initial reviews: i. Data imputation performance (R1.4, R1.7, R2.2), ii. Causality with respect to trans-regulator input (R1.6, R2.1), and iii. Additional benchmarking baselines and clarifications (R1.3, R2.3, R3.3, R3.8, R3.9). As a response to the feedback, we have substantially revised the manuscript and made significant improvements.

Key changes are as follows:

1. **We developed a new model, Corgi+, with improved imputation performance**

The original Corgi model was designed with cross-both generalization as the main goal, therefore it was not optimized for the data imputation task. Critically, Corgi cannot use information from the local RNA-seq context, as it needs to generalize to a completely new DNA sequence -where RNA-seq data might not even exist. Our new model, Corgi+, uses the local RNA-seq signal as additional context, and has several other improvements for increased imputation performance. We show that when using RNA-seq to impute epigenetic tracks, Corgi+ has 50-500% increased Pearson (delta) correlation compared to Corgi, and that it outperforms both Avocado and EpiGePT with a mean increase of 95.4% (Figure 4). We believe that this result shows that Corgi+ is the state-of-the-art in epigenomic data imputation using RNA-seq data.

2. **In-depth analysis on causal usage of *trans*-regulatory features**

We spent considerable effort on this analysis, resulting in 3 new main figures to the manuscript. First, we show that Corgi+ uncovers key regulators and uses them for its predictions in held-out tissues. Further, we show that contribution scores of *trans*-regulatory factors are correlated with gene expression z-scores (Figure 5). Second, we performed in silico mutagenesis in both cis and trans, and showed that effects of cis and trans knockouts are correlated across genomic bins (Figure 6). Finally, we show that Corgi can predict effects of GTEx eQTLs in held-out tissues, which, to our best knowledge, is reported for the first time (Figure 7).

3. **Additional baselines and clarifications**

We clarified the text and the Methods section further, according to the reviewers' points. Specifically, we clarified Corgi's unique contributions in the Introduction, expanded details on the genomic region selection process, reran the EpiGePT predictions and clarified the benchmarking setups. We also added additional informative baselines to the data imputation section.

Also we would like to note that during the course of the peer-review, our preprint was cited by an important review article on sequence-to-function models (doi.org/10.48550/arXiv.2602.01230).

Below, we reproduce the reviewers' comments verbatim and provide a point-by-point response. To facilitate cross-referencing, reviewer comments are numbered using R1 (Reviewer 1), R2, R3 and R4 as numbering notation.

Our responses are marked in blue, and any text changes to the initial submission are underlined.

Reviewer #1 (R1)

Ekin Deniz Aksu et al. introduced Corgi (Context-aware Regulatory Genomics Inference), a novel context-aware sequence-to-function model designed to predict human regulatory genomic features, including gene expression, chromatin accessibility, and various epigenetic marks. Remarkably, the model achieves high predictive accuracy even in previously unseen cell types. Corgi integrates multiple architectural components: convolutional layers to extract local cis-acting sequence features, a multilayer perceptron (MLP) to model trans-acting regulatory information, and FiLM (feature-wise linear modulation) layers to affine-transform and fuse these two sources of information. A Transformer module is further incorporated to capture long-range regulatory interactions. Through systematic benchmarking against EpiGePT and Borzoi under three evaluation strategies, the authors demonstrate the strong performance and robustness of Corgi. Below, I provide several suggestions that may help further improve the clarity and overall quality of the manuscript.

We thank the reviewer for their thorough assessment of our work.

R1.1. *In the main text, the authors highlight that they address a key challenge in context-aware sequence-to-function modeling—namely, the difficulty of integrating DNA sequences with context vectors due to their differing dimensionalities. However, existing methods such as EpiGePT, EPCOT, and EnformerCelltyping have already proposed effective strategies for fusing sequence information with contextual embeddings. Thus, given the current state of the field, this issue may no longer represent a major bottleneck. I suggest that the authors reconsider how this contribution is framed, or more clearly articulate the specific limitations of prior integration approaches that Corgi uniquely overcomes.*

The reviewer is correct in pointing out that existing tools such as EpiGePT, EPCOT and EnformerCelltyping can already integrate sequence and context information. EnformerCelltyping uses feature concatenation, which results in a much more complicated neural network architecture. EpiGePT also uses feature concatenation, and here another limitation lies in the computation of features which depends on known transcription factor binding motifs. Our FiLM overcomes this limitation. Generally, for methods that use DNA accessibility, integration is less of a challenge: since DNA sequence and accessibility share one axis, DNA accessibility signal can simply be concatenated to the one-hot-encoded DNA sequence as a fifth channel.

We agree with the reviewer that it would be better to mention these methods and the unique contribution of using FiLM in this context. Thus, we expanded the following section of the introduction:

> "We address a central challenge in context-aware sequence-to-function models: it is not straightforward to integrate DNA sequence with the context vector due to the difference in their dimensions. The context vector is one-dimensional, while the DNA sequence has two dimensions: length and features. Existing methods have used feature concatenation (ChromDragoNN[1], EpiGePT[2], EnformerCelltyping[3]) and cell type-specific decoders (scooby[4]) to integrate context vectors with DNA sequence information. Some methods use DNA accessibility information for their context, which simplifies integration as accessibility can be appended to the sequence information directly as a new channel (EPCOT[5]). We leverage the feature-wise linear modulation[6] (FiLM) technique to combine the two sources of information. FiLM was previously employed in various tasks such as image generation[7] and speech recognition[8], and has proven technically and

conceptually appropriate for this task. Importantly, using FiLM overcomes limitations of EpiGePT, as it allows all trans-regulators to influence learned *cis* features, without any dependence on known transcription factor binding motifs."

R1.2. *It is unclear what the variable n specifically refers to in Figure 2. Providing a clearer explanation would improve the readability and interpretability of the manuscript.*

The variable *n* in Figure 2a,b,c,e,f (new numbering) refers to the number of samples (genomic experiments from cell types) from each genomic assay type. However in Figure 2h (new numbering), *n* refers to the number of genes. We clarified this by editing the figure caption:

Figure 2. Corgi accurately predicts genomic coverage of multiple tracks in held-out cell types and sequences
(a), (b) and (c) [...] Sample size (N) refers to the number of biological samples. [...] (h) [...] Sample size (N) refers to the number of genes.

R1.3. *Several methods have been developed to predict cell type-specific epigenomic landscapes. In addition to EpiGePT, more recent approaches such as EPCOT and EnformerCelltyping are also representative in this domain. I recommend including these methods as additional baselines in the benchmarking experiments to more comprehensively demonstrate the advantages of Corgi. Moreover, the manuscript appears to understate the performance of EpiGePT and Borzoi. According to the original EpiGePT publication, its cross-cell type, cross-sequence, and joint cross-generalization performances are reported as 0.787, 0.504, and 0.515, respectively. As one of the leading sequence-only models, Borzoi should also exhibit cross-sequence performance close to its reported values. Although differences in data processing pipelines may cause some performance degradation, such substantial declines seem unlikely. Therefore, I suggest that the authors clarify the evaluation setup, data preprocessing steps, or experimental conditions to avoid potential misinterpretation of existing methods' performance.*

We would like to point out that both EPCOT and EnformerCelltyping use DNA accessibility data (DNase-seq or ATAC-seq) as their cell-state information, while Corgi uses gene expression data such as RNA-seq. As we explained above (R1.1), integrating gene expression and DNA sequence data is more challenging, due to the *trans* nature of the interactions. Additionally, whole genome DNA accessibility information is considerably more informative than *trans*-regulator expression values, and likely would give any model a huge advantage. And while all samples in our dataset have available gene expression data, not all of them have DNA accessibility information. Comparing Corgi versus EPCOT or EnformerCelltyping is therefore not directly possible and an attempt would present considerable challenges outside the scope of the current work. However, we do think that systematically comparing the power of DNA accessibility versus gene expression data in predicting epigenetic assays would be a valuable contribution to the field, and therefore started a new project to follow-up on this idea. We thank the reviewer for raising this point.

Furthermore, we would like to note that EnformerCelltyping cannot generalize cross-both, since it needs local chromatin accessibility information which would be missing in a completely held-out sequence. It could only be used with small-scale changes to a reference sequence, like SNPs, and under the assumption that the local ATAC-seq track will be relatively undisturbed upon the mutation.

Regarding the reported EpiGePT performances, the numbers that the reviewer cites (0.787, 0.504, and 0.515) refer to the DNase-seq track, while we observed a mean DNase Spearman correlation of 0.777 across the 5 cell types we tested using EpiGePT's webserver. So our tests are consistent with the reported performance. Additionally, we believe that the original EpiGePT publication might have inflated performance due to data splitting problems, where the authors have split both genomic regions and cell types **randomly**, which inflates performance due to data leakage. This is supported by the fact that cross-both performance was higher than cross-region in the published manuscript (0.515 vs 0.504).

We thank the reviewer for noting that differences in data processing may cause performance degradation. While we had mentioned this in our manuscript (initial submission, p.8: "Given that discrepancies in Pearson's r for some tracks may reflect minor differences in output signal scaling (squashed scale) between Corgi and Borzoi, we consider Spearman's ρ to be a more robust metric."), we understand that the Pearson correlation figure is confusing to interpret due to data processing differences (also raised by R2.3 and R3.9). Therefore, we removed Pearson correlation plots from Figure 3 and report only rank correlation which is robust to scale differences. The updated figure (below) shows that EpiGePT is competitive only for DNase-seq tracks, while Corgi outperforms it in other tracks (see also R3.8 below).

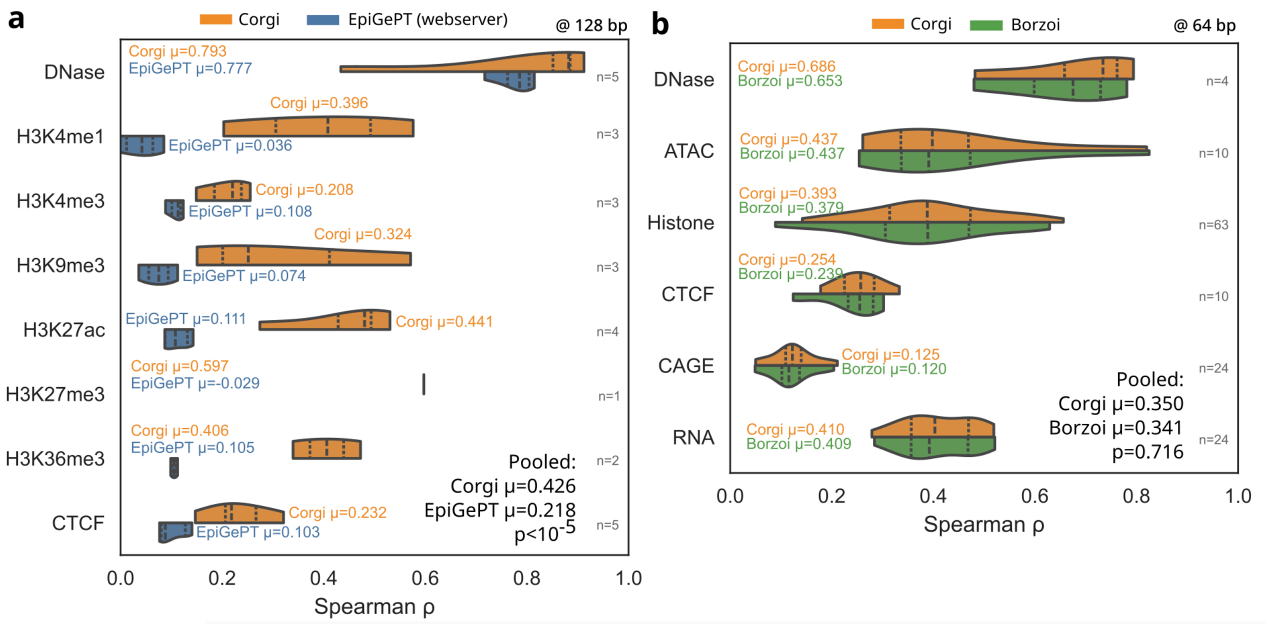

Figure 3a,b

R1.4. The presentation of results in the section "Corgi can impute epigenomic tracks in a cross–cell type setting" is not sufficiently clear. The previous section has already demonstrated Corgi's ability to generalize across cell types, so the manuscript should more explicitly articulate the conceptual and methodological distinction between the two sections. I suggest reorganizing this part to clarify its standalone contribution or how it differs from the preceding analyses. In addition, including brief concluding paragraphs at the end of each major section would improve the overall coherence and readability of the manuscript, while further highlighting Corgi's innovations and advantages.

We thank the reviewer for their concrete suggestions to improve clarity of the text. Based on this comment and other issues raised by the reviewers, we have removed this section from the manuscript and replaced it with a new section, titled **"Cross-cell type RNA-to-epigenomic data**

imputation with Corgi+", which we do not reproduce here in full due to its length. There we present imputation results using our new Corgi+ model and compare its performance to multiple baselines, always remembering the helpful presentation tips. Based on the reviewer's feedback, we made sure to convey the distinction of the imputation task from preceding benchmarks, and highlighted Corgi's innovations and advantages.

R1.5. In the section "Corgi understands cell type-specific regulation by learning biologically meaningful trans-regulators," the authors state that "genomic sequencing data is highly correlated across different contexts, and often simple baselines can yield good prediction accuracies." If biological signals are indeed highly correlated across contexts and simple baseline models can already perform well, it remains unclear why such a complex architecture is necessary to model context-dependent sequence-function relationships. The authors should clarify the necessity of this model complexity and the biological or predictive benefits it provides. Moreover, the way the results are presented in this section does not clearly convey the authors' main findings, which hampers the interpretability of this analysis. I suggest more clearly articulating the significance of these findings and how they support the conclusion that Corgi effectively learns cell type–specific regulatory mechanisms.

We addressed this concern in our new section on data imputation. Corgi+ outperforms the mean baseline in raw Pearson correlation, though the differences are minor because Pearson correlation on raw coverage values is an unsuitable metric. Using logFC over baseline as the metric, we see that the baseline (Corgi with avg. *trans*-regulator input) has very poor performance. We also see that Corgi+ has a good predictive performance and significantly outperforms Avocado and EpiGePT (Figure 4). So, we believe that indeed a complex architecture optimized for the imputation task is necessary to model context-dependent relationships.

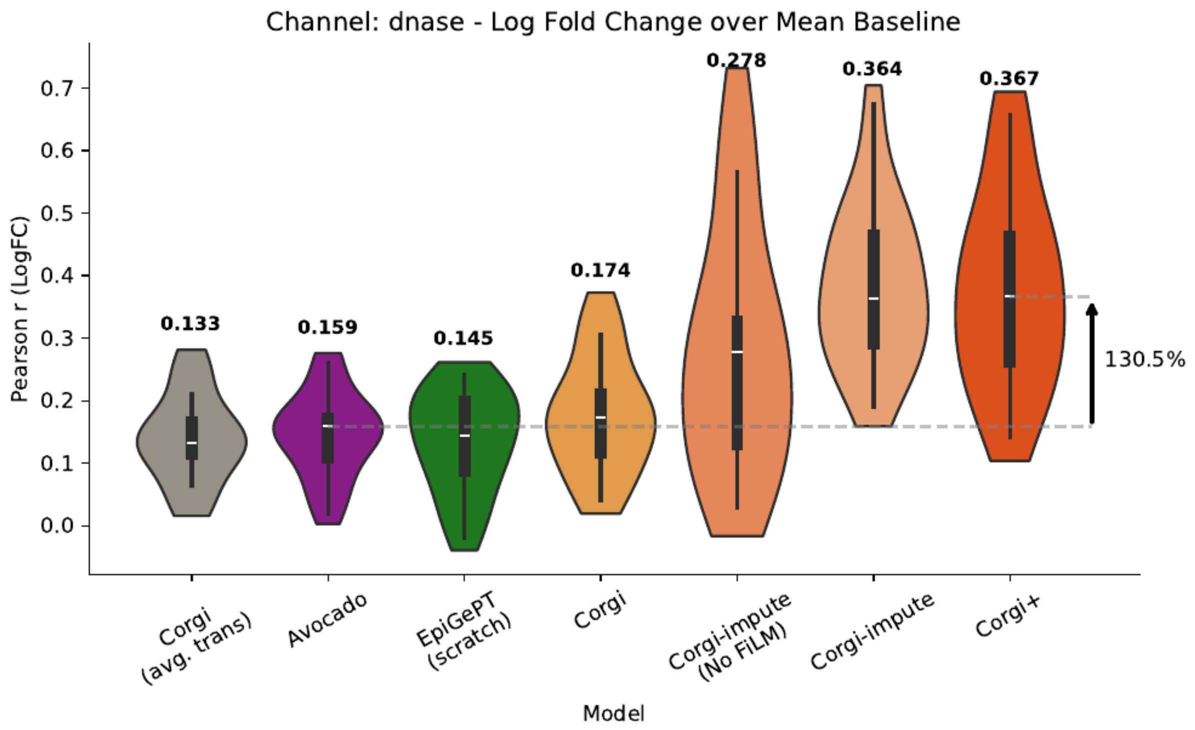

Excerpt from Figure 4c

R1.6. In the section "Corgi can identify upregulated trans-regulators in a zero-shot manner in held-out tissues," the authors claim that Corgi can identify upregulated trans-regulators in tissues that were never

seen during training. However, the manuscript does not clearly explain the mechanism by which the model achieves zero-shot identification—for example, how Corgi infers regulatory factors from previously unseen context vectors, what signals or internal representations enable this capability, and how this process relates to the model's supervision strategy. I suggest that the authors more explicitly describe Corgi's inference pipeline under zero-shot settings and articulate the theoretical or architectural basis for this capability. Providing such clarification would improve the clarity and credibility of this section and strengthen the overall persuasiveness of the manuscript.

As noted above, we have significantly expanded the analysis on causality with respect to the *trans*-regulator inputs, while making sure to explicitly describe the zero-shot inference and contribution score calculation pipeline. Importantly, we show that key cell type specific regulators are enriched within top-contributing *trans*-regulators in held-out tissues (Fig 5b, see below), and that using the incorrect *trans*-regulator expression vector decreases performance in predicting eQTL effects in held-out cell types (Figure 7d, see below).

b

iPSC

Term	Overlap	Genes	Adjusted P-value
Embryonic Stem Cell Undefined Human	8/48	FOXD3, SALL4, ZFP42, DNMT3B...	1.6e-08
Germ Cell Ovary Human	4/10	DAZL, DDX4, SALL4, POU5F1	6.7e-06
Germ Cell Embryo Human	4/10	DAZL, DDX4, NANOG, POU5F1	6.7e-06

Cardiac myocyte from RUES2

Term	Overlap	Genes	Adjusted P-value
AORTIC VALVE DISEASE	3/6	TBX20, GATA5, TBX5	1.1e-04
Cardiomyocyte Embryo Mouse	4/16	HAND2, HAND1, TBX20, GATA4	2.3e-04
Cardiosphere-derived Cell Heart Human	3/6	GATA4, NKX2-5, TBX5	2.7e-04

Liver (#276)

Term	Overlap	Genes	Adjusted P-value
Maturity onset diabetes of the young	3/26	HNF4A, ONECUT1, HNF1A	1.8e-02
Bile Acid Metabolism	4/112	AR, NR1I2, NR1H4, GNMT	3.5e-02
Xenobiotic Metabolism	5/200	ARG1, HNF4A, PLG, ESR1...	3.5e-02

Liver (#277)

Term	Overlap	Genes	Adjusted P-value
Maturity onset diabetes of the young	3/26	HNF4A, ONECUT1, FOXA3	2.4e-02
Herpes simplex virus 1 infection	9/498	ZNF320, ZNF331, ZNF83, ZNF479...	3.6e-02
Hepatocyte Liver Human	3/31	CEBPA, ARG1, HNF4A	4.7e-02

Neural progenitor from H9

Term	Overlap	Genes	Adjusted P-value
Embryonic Stem Cell Undefined Human	6/48	SOX2, SALL4, ZFP42, PRDM14...	2.9e-05
Germ Cell Liver Mouse	3/5	SOX2, PRDM14, POU5F1	1.1e-04
Neural Stem Cell Undefined Human	5/40	SOX2, OTX2, SOX11, HOXB1...	1.1e-04

Testis

Term	Overlap	Genes	Adjusted P-value
Spermatogonial Cell Testis Human	3/31	DAZL, DMRT1, BRDT	1.0e-02
Embryonic Mesodermal Cell Embryonic Mesoderm Mouse	2/6	HOXB1, TBXT	1.0e-02
Primordial Germ Cell Embryo Mouse	2/11	RBMXL2, PRDM14	1.1e-02

Figure 5b

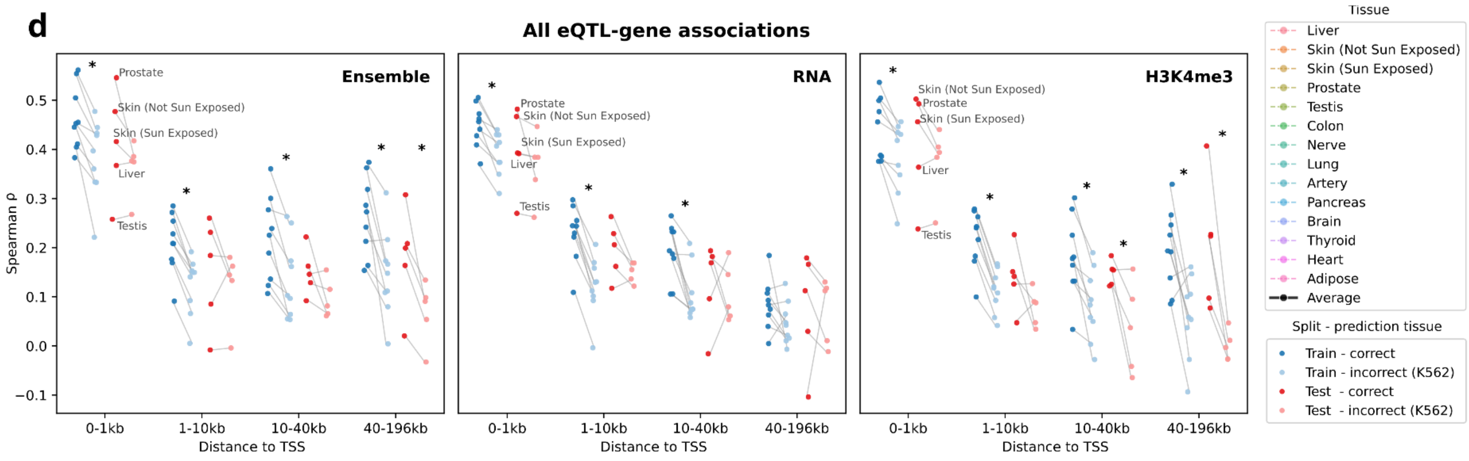

Figure 7d

We have added the following sections to the main manuscript, with 3 new main figures:

"Corgi+ uncovers key cell type-specific trans-regulators in a zero shot manner",
"Analysing cis and trans TF contributions with in silico knockouts", and
"Corgi predicts eQTL variant effects in held-out cell types".

We believe that these additions significantly improved interpretability of the model and strengthened the persuasiveness of the manuscript. We hope that they answer the reviewer's concerns regarding this point.

R1.7. *In the discussion section, the authors note that Corgi is capable of inferring missing experimental assays using RNA-seq data. However, the results section provides only limited evidence supporting this key capability. Given that such cross-modal inference represents one of Corgi's core innovations, I recommend including more systematic and comprehensive experiments and analyses in the results section to highlight Corgi's ability to use RNA-seq data to impute epigenomic features. Doing so would more fully demonstrate the model's unique contributions and practical potential.*

With systematic benchmarking, we have shown that Corgi+ represents a significant step in prediction of epigenomic features using RNA-seq data. We refer the reviewer to our new section **"Cross-cell type RNA-to-epigenomic data imputation with Corgi+"** , explained in R1.4 and R1.5. We wholeheartedly thank the reviewer for their suggestions on expanding this section, as we believe that Corgi+ represents a strong achievement in this task, having potential to be widely beneficial to a multitude of researchers with RNA-seq data and who want to impute DNase and ChIP-seq tracks.

Reviewer #2 (R2)

Summary:

Aksu et al. develop a deep learning model called Corgi, which is capable of predicting transcriptomic and epigenomic coverage tracks in unseen cell types and tissues. The model is largely based on the Borzoi/Enformer architecture, but has an additional MLP network that receives trans-regulatory expression values as an additional input vector, which allows the authors to predict what coverage tracks should look like in new contexts by passing an appropriate trans-regulatory expression vector to the model. The authors evaluate their model at the tasks of predicting gene expression and epigenomic activity in three settings: (1) held-out genomic sequences, (2) held-out tissues or cell types, and (3) held-out sequences and cell types. They demonstrate competitive performance to state-of-the-art methods while introducing the new capability of predicting coverage in unseen cell types.

Significance, novelty and utility:

An interesting study that introduces a new approach for modeling sequence-to-function tasks without the multi-tasking paradigm. As noted by the authors, their approach is conceptually similar to methods like scooby, but with important differences (inherently interpretable input space consisting of trans-regulatory expression values as opposed to a low-dimensional embedding, and applied to bulk- and cell type data).

I have a number of concerns and comments detailed below. The largest concern is already discussed by the authors: the apparent inability of the model to causally predict differences in coverage tracks under slight

perturbation of the trans-regulatory context. This is something that the scooby paper actually demonstrated somewhat successfully (Fig. 3 of https://www.nature.com/articles/s41592-025-02854-5).

The reviewer's comments regarding causality and imputation performance are very important. Therefore, we put a focus on these two areas in the revised work which led to substantial imputation performance improvements and unlocked Corgi's previously untapped capability to predict eQTLs in held-out cell types. We believe that these have significantly strengthened our manuscript and we are grateful to the reviewer for their evaluation and constructive feedback.

Comments and suggested improvements:

Major:

***R2.1**. I am not fully convinced the Corgi model truly uses the trans-regulator input vector in a biologically meaningful and causal way when making its predictions. As the authors describe in the discussion, they fail to observe expected predicted changes in coverage tracks when increasing or decreasing the input expression value of a relevant trans-regulator. Ideally, I'd like to see an analysis similar in concept to the scooby paper (Fig. 3), where its clear that the model's predictions (and cis-regulatory contribution scores of a given TF motif) increase/decreases as a function of the trans-regulator concentration. Otherwise, it is hard to believe that these features are used causally by the model, as opposed to Corgi learning an arbitrary mapping from these vectors (as dictionary identifiers of sorts). At the very least, I'd like to see a variant effect prediction analysis on fine-mapped eQTLs, where Corgi is successfully able to predict beta coefficients of held-out tissues (e.g., liver).*

In order to show that Corgi uses the *trans*-regulator expression in a biologically meaningful way, as explained above, we have performed an in-depth analysis of causality with respect to *trans*-regulators. We have added three new sections and three new main figures to the manuscript on this point. Specifically, we show that i. Corgi+ uncovers key regulators and uses them for its predictions in held-out tissues (Figure 5), ii. contribution scores of *trans*-regulatory factors are correlated with gene expression z-scores (Figure 5), and iii. *cis* and *trans* in silico mutagenesis effects are correlated across genomic bins (Figure 6). We especially find the gene set enrichment results highly significant, since we show that the model can pick up very important cell type-specific regulators and pathways consistently across all held-out test contexts (Figure 5b).

Even though Corgi models are not very sensitive to *trans*-regulator perturbations (as exemplified by the need to use a signal amplifier in the knockout effect visualizations in FIg. 6b), they often have a biologically meaningful direction of change. This is shown by the modest but positive correlation between cis and trans KO effects across genomic bins. Additionally, we would like to point out that using the trans-regulator expression as a dictionary identifier would only be possible in cell types within the training set, and not in held-out cell types.

Finally, we have shown that Corgi can predict beta coefficients of GTEx eQTLs in held-out tissues, which, to our best knowledge, is reported for the first time (Figure 7, also see below). Furthermore, our analysis of comparing performances of using the correct vs incorrect *trans*-regulator vector shows that Corgi's variant effect prediction capability is dependent on using the correct *trans*-regulator vector, which we think clearly shows causality (Figure 7d). We thank the reviewer again for this suggestion.

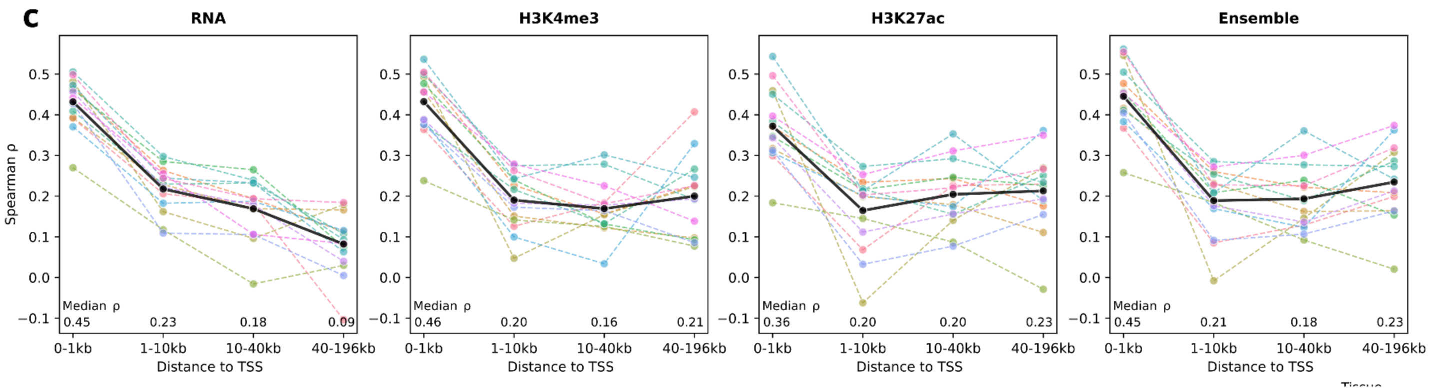

Figure 7c

***R2.2.** In the analysis presented in Figure 4b, I am hesitant to believe that the improvement over the mean baseline is statistically significant. Can the authors show this? Also, is there a reason why Corgi's performance improvement over Avocado in Figure 4a (or over Baseline in 4b) only looks significant for RAMPAGE? On a similar note, how well can Corgi predict gene expression or chromatin accessibility of held-out sequences for very cell-type specific loci (as opposed to cell type-agnostic, house-keeping genes, where the mean baseline is sufficient)?*

We agree with the reviewer that the improvements over the mean baseline in our initial manuscript were miniscule, and the reviewer was right to be hesitant. As explained above, we have made significant improvements in this area with our new model Corgi+, which is optimized for the data imputation task and is state-of-the-art when used to impute epigenomic tracks from RNA-seq data (Figure 4). Furthermore, we believe that using the more appropriate Pearson (logFC over baseline) metric answers the reviewer's last point regarding cell type-specific vs housekeeping loci, since housekeeping loci would have a logFC around zero, and thus the correlation coefficient calculation naturally focuses at more cell type-specific loci.

On the point regarding performance differences in the RAMPAGE track, we want to elaborate here, since we do not measure RAMPAGE performance any more in the new imputation benchmarks shown in Figure 4. The reason is that since all models have access to full RNA-seq information, predicting CAGE and RAMPAGE becomes a less interesting task. In practice we observed RAMPAGE Pearson (delta) correlations of 0.58 and 0.48 in CAGE.

The fact that Avocado struggles with CAGE/RAMPAGE predictions was observed before. Below, we reproduce Figure S4 from the EPCOT paper, which shows Avocado's poor performance in CAGE predictions.[5]

[editorial note: third party material redacted]

Minor:

R2.3. *In Figure 3c, it would be prudent to compare the two models' on bin-level Pearson R metrics after (un-)transforming their predictions to the same scale. Additionally, Corgi is at 64 bp resolution while Borzoi is at 32 bp, and I would suggest average-pooling Borzoi's predictions to 64 bp before comparing, for fairness.*

We agree with the reviewer that having different scales confounds interpretation of the Pearson r metric. As explained in R1.3, while we had mentioned this in our manuscript (initial submission, p.8: "Given that discrepancies in Pearson's r for some tracks may reflect minor differences in output signal scaling (squashed scale) between Corgi and Borzoi, we consider Spearman's ρ to be a more robust metric."), we understand that the Pearson correlation figure is confusing to interpret due to data processing differences. Therefore, we removed Pearson correlation plots from Figure 3 and report only rank correlation which is robust to scale differences.

Regarding resolutions, Figure 3 already shows correlations at the correct resolution. We refer the reviewer to the following section of the Methods in the initial submission (p.21):

> "Since Borzoi predicts at a 32 bp resolution, we applied average pooling to match the 64 bp resolution used by Corgi."

In order to avoid any possible confusion on the part of the reader, we have now labeled the used resolutions on top of the violin plots in Figure 3a and 3b (see above, R1.3).

R2.4. *It would be good to compare the mean-subtracted performance metrics in Figure 5a and 5b to Borzoi's (or other SOTA models') performance.*

The comparison with Borzoi uses only a subset of experiments from the cross-sequence benchmark, due to the need to match specific Borzoi tracks with Corgi samples (explained in

Methods, section “Benchmarking”, also see Supplementary Table 4). Therefore, this analysis cannot be put on the same plot as Figure 5a and 5b, so we included this analysis in the new Figure S5, as seen below.

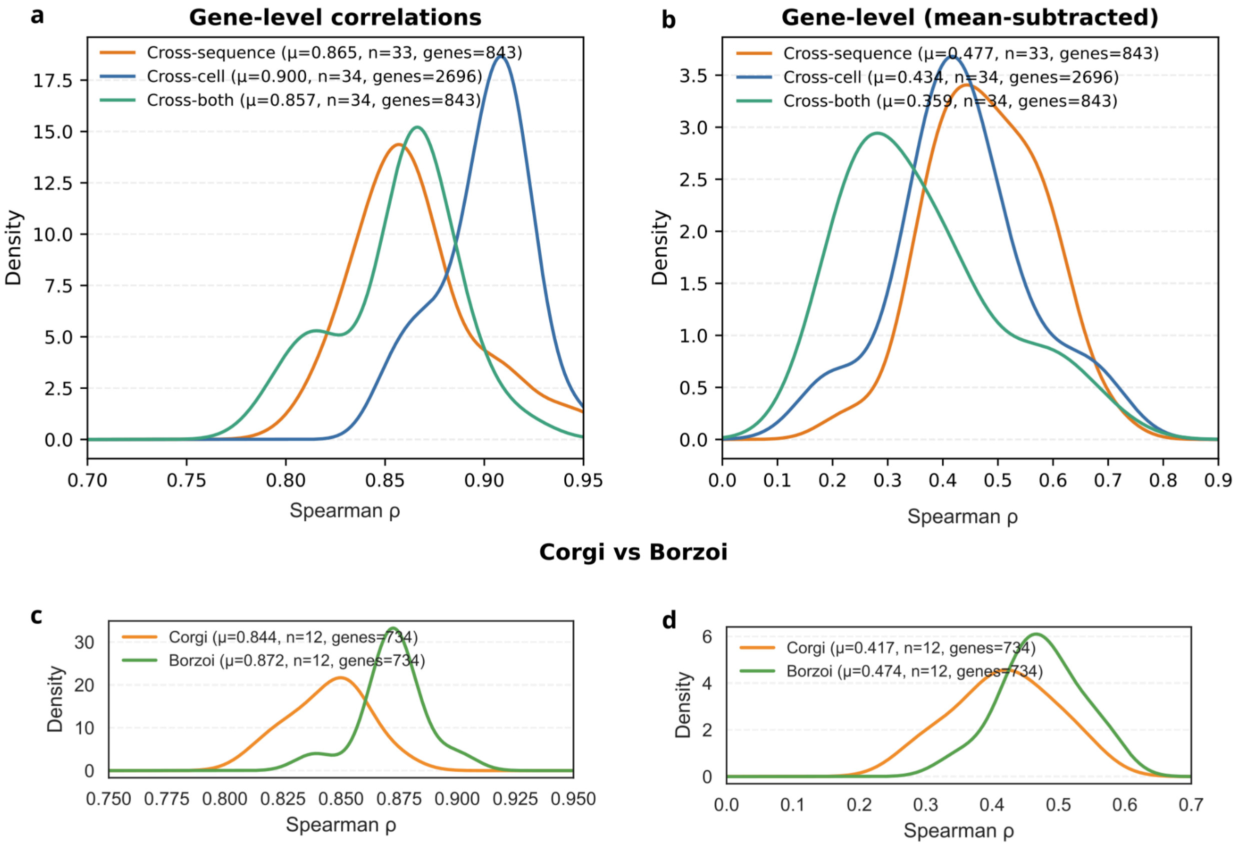

Figure S5. Gene-level spearman correlations

(a) shows distribution of gene-level Spearman rho coefficients between Corgi predictions and ground truth in the three benchmarks, and (b) shows Spearman rho coefficients between mean-subtracted Corgi predictions and mean-subtracted ground truth. (c) shows the distributions of gene-level Spearman rho coefficients for Corgi and Borzoi. This is a cross-sequence benchmark. (d) same data as (c) with mean-subtraction. Borzoi has a measurable edge over Corgi in this setting.

Reviewer #2 (Remarks on code availability):

The code is available on github and documented.

Reviewer #3 (R3)

Summary:
The authors present a deep learning genomic sequence-to-function model, “Corgi,” that includes context-specific expression levels of trans regulators as an auxiliary input with the goal of generalizing to unseen cell types. Strong performance is reported on the most difficult task of predicting expression of held-out genes in held-out cell types. Comparisons to the state-of-the-art model, Borzoi, are mixed.

Assessment: Overall, the Corgi strategy is a promising direction and a good application of feature-wise linear modulation (FiLM). However, it is not clear how performance improvements are achieved, which is important for evaluating how much trans regulator features contribute to predicting gene expression. To

address this, the manuscript would benefit from additional baselines (e.g., mean gene expression in the training set) and evaluations. A few inconsistencies and likely influential filtering decisions are also noted.

We thank the reviewer for their time and their assessment of our work. We provide point-by-point responses to their concerns below.

General Comments:

R3.1. *Transcriptomics data were "harmonized" across assays by quantile normalization, log transformation and batch correction. As shown in the example in supplementary figure S3 (c)-(d), this results in high correlation between CAGE and RNA-seq from the same tissue. Since these assays measure different aspects of transcription, more justification is needed to support the assumption that the two assays "should" have a higher correlation than is observed in the raw data, and that harmonizing the two would be superior to alternative approaches (e.g. including both CAGE-seq and RNA-seq expression levels as distinct elements of the trans-regulator expression vector).*

CAGE (Cap Analysis of Gene Expression) measures mRNA expression by sequencing the first dozens of base pairs after the 5' cap [9], while RNA-seq uses cDNA conversion (after potential enrichment for certain transcripts such as polyA-tail including ones). Essentially, for protein coding transcripts, they both measure the concentration of mRNA in the cell. Many studies were performed showing that CAGE and RNA-seq experiments performed on the same sample are highly correlated. We would like to show below as examples, Figure 3A from Kawaji et al. [10] (left) and Figure 4A from Gacita et al. (right) [11], both showing high correlations between gene expression values calculated from CAGE and RNA-seq (0.88, 0.66 and 0.78).

[editorial note: third party material redacted]

Regarding the last point, the reviewer makes the argument that it is unclear whether harmonization would be superior to using both RNA-seq and CAGE data in the input. We agree that this is not clear, and therefore we did not make any such claim in the manuscript. However, this alternative approach would come with a potentially significant drawback in limiting the size of our training data, since only a small portion of our data has both RNA-seq and CAGE data available. Additionally, it would make Corgi considerably more impractical to use for other researchers, due to the need to have both RNA-seq and CAGE information as input. Therefore, we opted for harmonization here as a practical solution, rather than using inputs with clear batch effects, as shown in Figure S3 of our manuscript.

R3.2. *The authors go to great lengths to avoid leakage between the training and test sets, which is admirable. But these procedures greatly reduce the size of the test set (~4-fold), and likely biases it as well. We have several suggestions related to this:*
1. Corgi is trained on the same genomic sequences and train-validation-test split as Borzoi, with the caveat that all windows that include a trans-regulator gene are excluded (to prevent data leakage from the context vector). While a sound approach, this reduces the number of 524,288-bp windows from 6,888 to only 1,437. Please acknowledge this clearly in the main text and discuss why this is a reasonable trade-off.
2. When bin-level correlations are computed, it appears that only 637 windows were used (Benchmarking section of Methods). Please clarify how the 637 were selected and justify this decision in the manuscript.
3. Excluding trans regulator genes (and their neighbors) from the test set may have eliminated many of the genes that exhibit the most context-specific patterns of gene expression. We recommend evaluating the variability in expression across cell types of the excluded versus retained genes to assess if the filter biased the test set towards (or away) from broadly expressed genes.

We would like to thank the reviewer for their scrutiny on this point. Avoiding leakage is indeed one area that we paid a lot of attention to, and where we believe Corgi improves over previous approaches (as an example, EpiGePT used random splits).

Firstly, we would like to clarify that our procedure does not greatly reduce the size of the test set. We believe that the reviewer used the Borzoi genomic sequences file (at https://github.com/calico/borzoi/blob/main/data/sequences_human.bed.gz) and counted the number of sequences there in ‘fold3’, which comes to exactly 6,888. However, these sequences are not 524,288 bp, but only 196,608 bp long. So these sequences were not directly used in Borzoi training, but they were modified. Also, these regions are tiled across the genome, and they overlap.

In order to clarify our exact procedure and avoid any misunderstandings, we expanded the relevant section of the Methods:

> The genomic sequences were split according to Borzoi’s folds[12], with fold 3 reserved for testing and fold 4 reserved for validation similar to Borzoi. Specifically, we merged overlapping sequences in the Borzoi folds bed file using bedtools merge. We made sure that sequences from different folds never overlap, which is indeed the case. Then, we subtracted trans-regulator gene coordinates from the merged regions from folds 3 and 4 using bedtools subtract. For each merged region, we tile 524,288 bp sequences with a stride of 174,764 bp. This ensures that each base pair is represented in approximately three sequences, while also shifting the bin boundaries. At the ends of merged regions, there is unused sequence left over. If this sequence is larger than 50 kb, we

add a new sequence that ends exactly at the end of the merged region. Excluding gene bodies of *trans*-regulatory factor genes from the validation and test folds is important, as their TPM values are used in the input. This procedure resulted in 11,496 sequences of length 524,288 bp for training, 1462 sequences for validation and 1437 for testing, in a tiled fashion across most of the human reference genome (Supplementary Table 3). In total, we train on 2.2 Gbp of DNA sequence and test on 292 Mbp.

On the second point regarding 637 windows, as mentioned in the text, this number simply arises from tiling of the test fold regions with a stride of 393 kb.

Here we have made the following clarifications:

~~For binwise correlations in the cross-sequence and cross-both settings, 637 genomic regions of 524 kb were used by tiling the test fold with a stride of 393 kb. For each of these input regions, Corgi predicts genomic tracks with a 64 bp resolution, resulting in prediction values for 8192 genomic bins, which is cropped to the central 6144 bins.~~

For binwise correlations in the cross-sequence and cross-both settings, the merged test fold regions were tiled using a stride of 393 kb. This procedure generates 637 genomic regions of 524 kb for testing. For each of these input regions, Corgi predicts genomic tracks with a 64 bp resolution, resulting in prediction values for 6144 genomic bins, corresponding to the central 393 kb of the input region.

Regarding the third point, we hope that the above clarification ameliorates some of the reviewer's concerns (e.g. neighbors of *trans*-regulator genes were not removed). Nevertheless, to address this concern thoroughly, on the suggestion of the reviewer, we compared the variance and 'variance divided by mean' of gene expression values of *trans*-regulator genes versus the rest, across total RNA-seq samples of our dataset. We did not find a significant difference between the variation in *trans*-regulators and other genes.

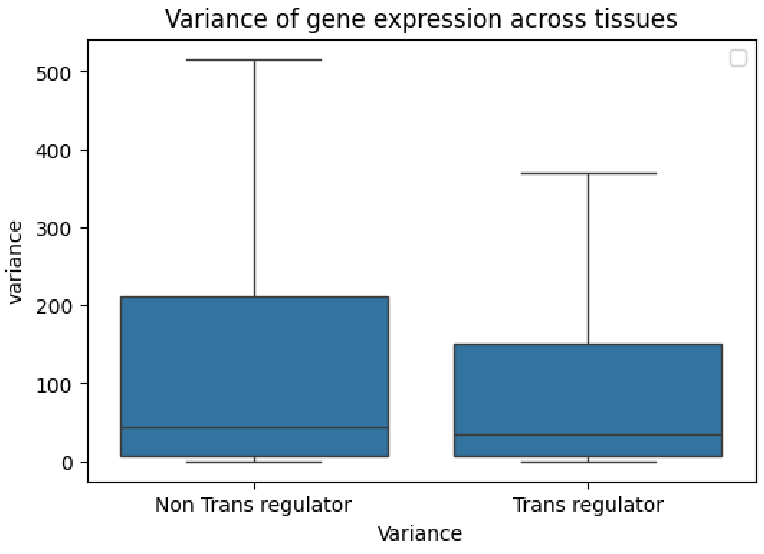

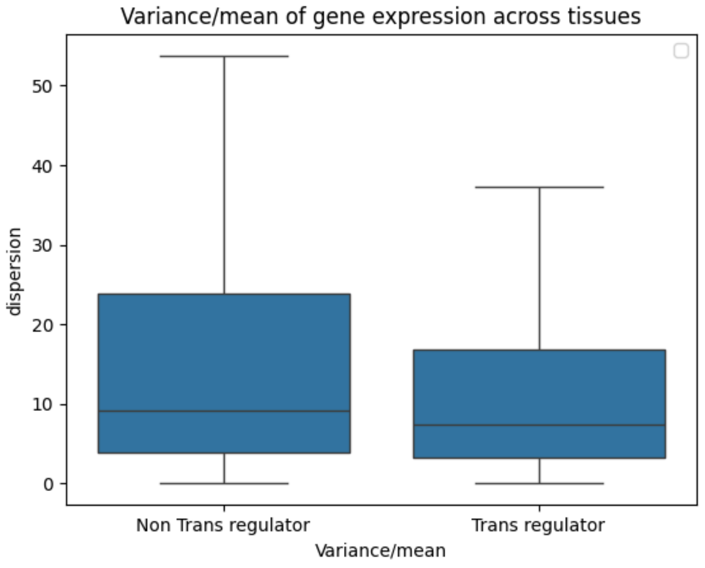

R3.3. It is appealing to conclude that Corgi's trans-regulatory context vector is being used to enhance the performance of the model. However, the results do not explicitly show this. To clarify the value added from the trans regulator inputs, we recommend performing additional comparisons in the "cross-cell type"

evaluation. First, the correlation between observed gene expression in each test cell type should be compared to two simple baselines: 1) the average observed expression among all training cell types, and 2) Corgi's predictions given a context vector composed of the average trans-regulator expression level among all training cell types. These comparisons will clarify if Corgi is doing more than predicting the typical expression level of each gene. In addition, it is recommended that Corgi-like models be trained using only sequence inputs and using only trans regulator inputs. Then these models should be compared to the Corgi model (trained using both inputs) across the various evaluation benchmarks in the manuscript. This would help to quantify, all other things being equal, the contributions of sequence and trans regulator expression.

We thank the reviewer for their excellent suggestions on additional baselines. As the reviewer will see, we have significantly overhauled and expanded the cross-cell type data imputation section, and we included many of these baseline suggestions in this section.

Specifically, we use Corgi (avg. trans), as "given a context vector composed of the average trans-regulator expression level among all training cell types". Additionally, we trained new models called Corgi-impute and Corgi-impute (No FiLM), in order to quantify the contribution of the *trans*-regulator expression vector. Ablating the sequence module is not trivial, since this is a core part of sequence-to-function models. Therefore we could not add this baseline to our testing.

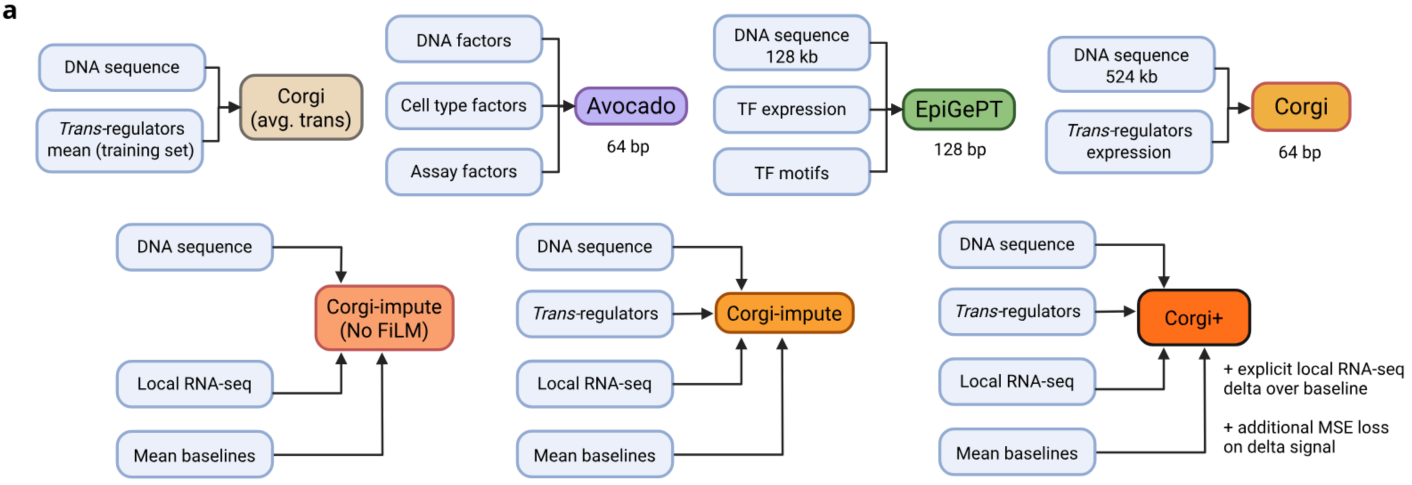

Figure 4a

We refer the reviewer to the new section in our revised manuscript, titled "**Cross-cell type RNA-to-epigenomic data imputation with Corgi+**". We reproduce a relevant paragraph here for convenience:

> "In order to test the effects of these optimizations on data imputation performance, and to compare sequence-based models to a tensor-based model, we trained multiple models on a subset of the Corgi training data (Figure 4a). In addition to information used by Corgi, Corgi-impute also uses the local RNA-seq coverage track at 64 bp resolution, as well as the mean training baseline signal from all Corgi tracks. In order to test the effect of conditioning on *trans*-regulator expression, we trained Corgi-impute (No FiLM), and we included Corgi (avg. trans) as a baseline, which uses the average training *trans*-regulator expression regardless of the chosen cell type. Finally, we designed a new model that we call Corgi+, with additional imputation-optimized input and objective functions. We compared the imputation performance of the Corgi family to Avocado and EpiGePT (scratch),

which we trained from scratch and validated on the same training and validation data as Corgi-impute and Corgi+."

R3.4. The novelty of Corgi should be tempered slightly in the text in recognition of existing methods for various components of Corgi, such as Avocado for imputation, Borzoi (and related models) for multi-task sequence-to-activity predictions, and EpiGePT (and related models) for epigenetic track predictions based on sequence plus trans regulator expression. The authors cite and benchmark against these methods, which is great. Our recommendation is simply to talk about these existing strategies in the introduction and carefully delineate what is novel in Corgi (e.g., combining aspects of these different methods, using FiLM).

We have rephrased the relevant paragraph of the introduction accordingly:

> "We address a central challenge in context-aware sequence-to-function models: it is not straightforward to integrate DNA sequence with the context vector due to the difference in their dimensions. The context vector is one-dimensional, while the DNA sequence has two dimensions: length and features. Existing methods have used feature concatenation (ChromDragoNN[1], EpiGePT[2], EnformerCelltyping[3]) and cell type-specific decoders (scooby[4]) to integrate context vectors with DNA sequence information. Some methods use DNA accessibility information for their context, which simplifies integration as accessibility can be appended to the sequence information directly as a new channel (EPCOT[5]). We leverage the feature-wise linear modulation[6] (FiLM) technique to combine the two sources of information. FiLM was previously employed in various tasks such as image generation[7] and speech recognition[8], and has proven technically and conceptually appropriate for this task. Importantly, using FiLM overcomes limitations of EpiGePT, as it allows all *trans*-regulators to influence learned *cis* features, without any dependence on known transcription factor binding motifs."

Specific Comments:
***R3.5.** Preventing leakage: Evaluating a model's generalization capability in held-out cell types is difficult because of the highly correlated patterns of gene expression across cell types. The authors devise a clustering-based strategy for preventing data leakage of this type by applying agglomerative clustering to 580 samples according to their gene expression levels, then removing clusters from the training set until a desired number of validation and testing clusters was reached. This seems like a good approach. But (1) insufficient detail is provided on the exact clustering methods used, as well as how clusters were selected for inclusion into validation or test sets, and (2) performance on the "easy-test" set (constructed by randomly sampling clusters from the training set) is not reported. Describing the clustering method and including results for the "easy-test" set would be very helpful for contextualizing the presented performance results.*

We have expanded the Methods section, describing the clustering and selection procedures in more detail:

> **Training-test split**
> In order to prevent any data leakage across training and test sets, we carefully split our samples into subsets that are as dissimilar as possible. First, we organized the 580 samples into 40 clusters through agglomerative clustering according to their trans-regulator expression levels. Expression levels were scaled using sklearn.preprocessing.StandardScaler.fit_transform, and clustering was

> performed with sklearn.cluster.AgglomerativeClustering using default settings. Using manual curation, we left out entire clusters out of the training set so that a suitable number of validation and test samples were reached, also making sure that all assays are available in multiple samples in the test set (Figure S2). This curation was performed prior to any benchmarking and was not repeated. Second, for all selected validation and test samples, similar cell types or tissues still left in the training set were determined by a keyword match and matching samples were taken out of the training set. For example, since liver is in the test set, leaving a hepatocyte sample in the training set could constitute data leakage, which we avoided. Lastly, a random sample was selected from all training clusters into an "easy-test" set, for a testing scenario where the exact samples are unseen by the model, but similar cell types and tissues were seen during training. In practice, we did not observe significant performance differences between the cross-sequence and cross-both easy-test settings (mean *r* 0.408 vs 0.378, p=0.068), and did not perform additional analyses on this set.

R3.6. Harmonization: In the main text, it is implied, but not explicitly stated, that the harmonization procedure was applied only to trans regulator expression levels. This should be clarified.

We emphasised that the harmonization was applied only to *trans*-regulator expression levels by rephrasing the sentence from our manuscript (p.4):

> "In order to use the diverse training set most efficiently, we first harmonized those input *trans*-regulator expression levels that were obtained by different transcriptomic assays by quantile normalization, log transformation and batch correction."

R3.7. Figure 2d: Corgi's performance on "cross-both" matches or exceeds its performance on "cross-sequence" gene expression predictions, with both tasks reporting extraordinarily high Pearson's correlations (>0.9) that exceed the performance of "cross-cell" predictions. It is surprising that "cross-both" would outperform "cross-cell", since both tasks involve generalizing to a held-out cell type with "cross-both" presenting the additional challenge of predicting on genes not seen during training. Is there an error here?

We thank the reviewer for raising this issue. We have double checked the analysis in this section. Since cross-both and cross-cell type settings measure performance on different subsets of genes, they cannot be directly compared. However, it is true that one would not expect to see a higher accuracy in the cross-both setting. We noticed that this discrepancy stems from different numbers of zero-expression genes in the cross-cell type and cross-both settings. Thus, we have excluded these genes from the analysis, which results in a slight reduction in the correlations. Additionally, different from the initial submission, we use coverage signal from entire gene bodies including introns, instead of summing only exonic coverage. This results in a slight performance increase, possibly due to most of the samples being prepared with total RNA capture. In the end, gene-level correlations in the initial submission (before, left) and the revised manuscript (after, right) can be seen below, which shows that cross-both performance does not outperform the other benchmarks any more.

Before

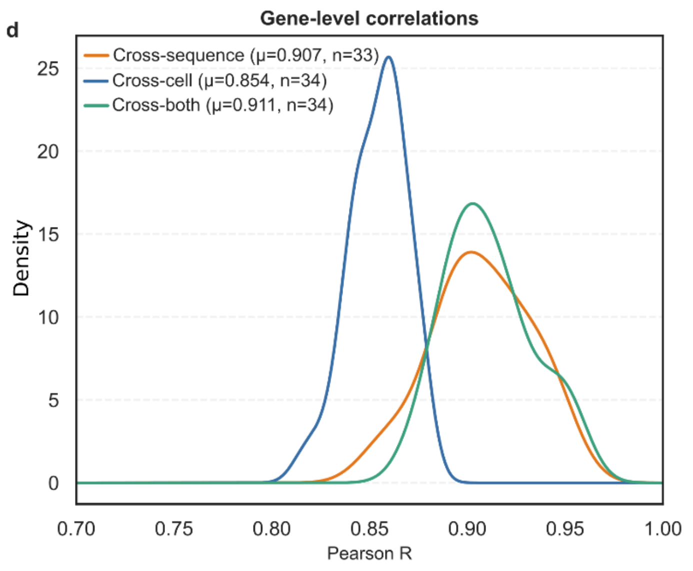

After

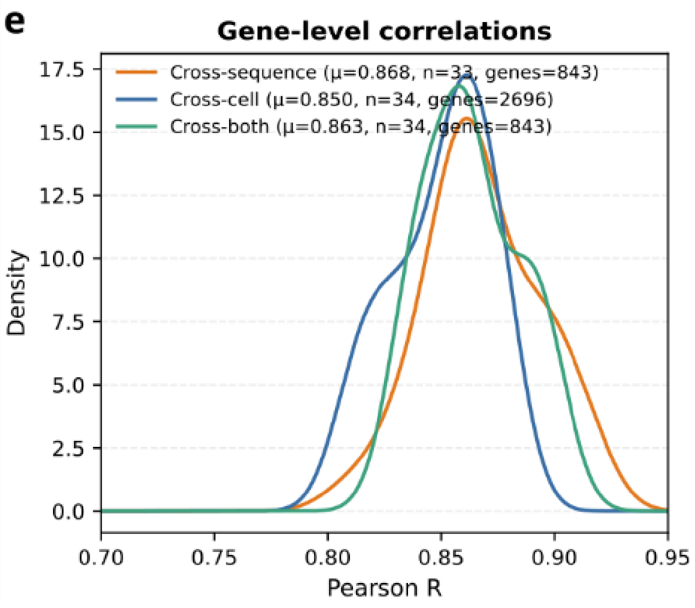

Accordingly, we have made the following changes to the text (*Methods*, Benchmarking):

> For gene-level benchmarking, we measured the sum of predicted vs observed coverage overlapping with gene coordinates in RNA-seq tracks, and log-transformed the values. Genes with zero expression across all samples were excluded, in order to avoid an inflation in reported accuracy values.

R3.8. *Figure 3: EpiGePT's performance is consistently shown to be dramatically inferior (panels (a)-(b)) and its coverage track predictions appear very poor qualitatively (panel (e)). Given the stated difficulty in getting EpiGePT's code to run, this suggests that perhaps parameter optimization did not converge. This analysis needs to be revisited/repeated.*

As mentioned in *Methods* from our initial submission (p.20), we did not train EpiGePT in this benchmark, but used the webserver that uses a checkpoint trained on ENCODE data.

> "We were unable to run the provided code to train our own version of EpiGePT, and thus we used the online prediction tool at https://health.tsinghua.edu.cn/epigept/. The published model was trained on 104 cell types on hg38, including samples corresponding to tissues from our test set. Additionally, the genomic regions to be trained on were selected randomly (as opposed to leave-one-chromosome-out or homology-controlled folds), which should also give an advantage to EpiGePT. [...] All five cell types were included in EpiGePT's training set, giving EpiGePT a further advantage in this benchmark."

The EpiGePT model used by the webserver was trained on ENCODE data, similar to the bulk of the Corgi dataset. Inputs to the webserver are a bed file of regions from hg38, and a csv file of TF expression information, which is quantile normalized using EpiGePT's own reference distribution. Motivated by the comment from the reviewer, we have investigated this pipeline thoroughly. We have found a bug in our code that prepares the TF expression file, which resulted in incorrect expression values to be used. We fixed this error and recomputed all EpiGePT predictions using the correct expression values. DNA regions were unchanged.

EpiGePT predictions did not visibly change in this second run. An example result can be seen below for DNase-seq, H3K4me3 and H3K27ac (blue=ENCODE, red=Corgi, dark green=EpiGePT (correct expression), light green=EpiGePT (incorrect expression). EpiGePT can identify DNase-seq peaks at promoters, but overall has high noise in intergenic regions and poorly recapitulates the observed signal. This might stem from the MSE loss that EpiGePT uses, which is suboptimal to train sequence-to-function models since genomic and epigenomic data are heteroskedastic.

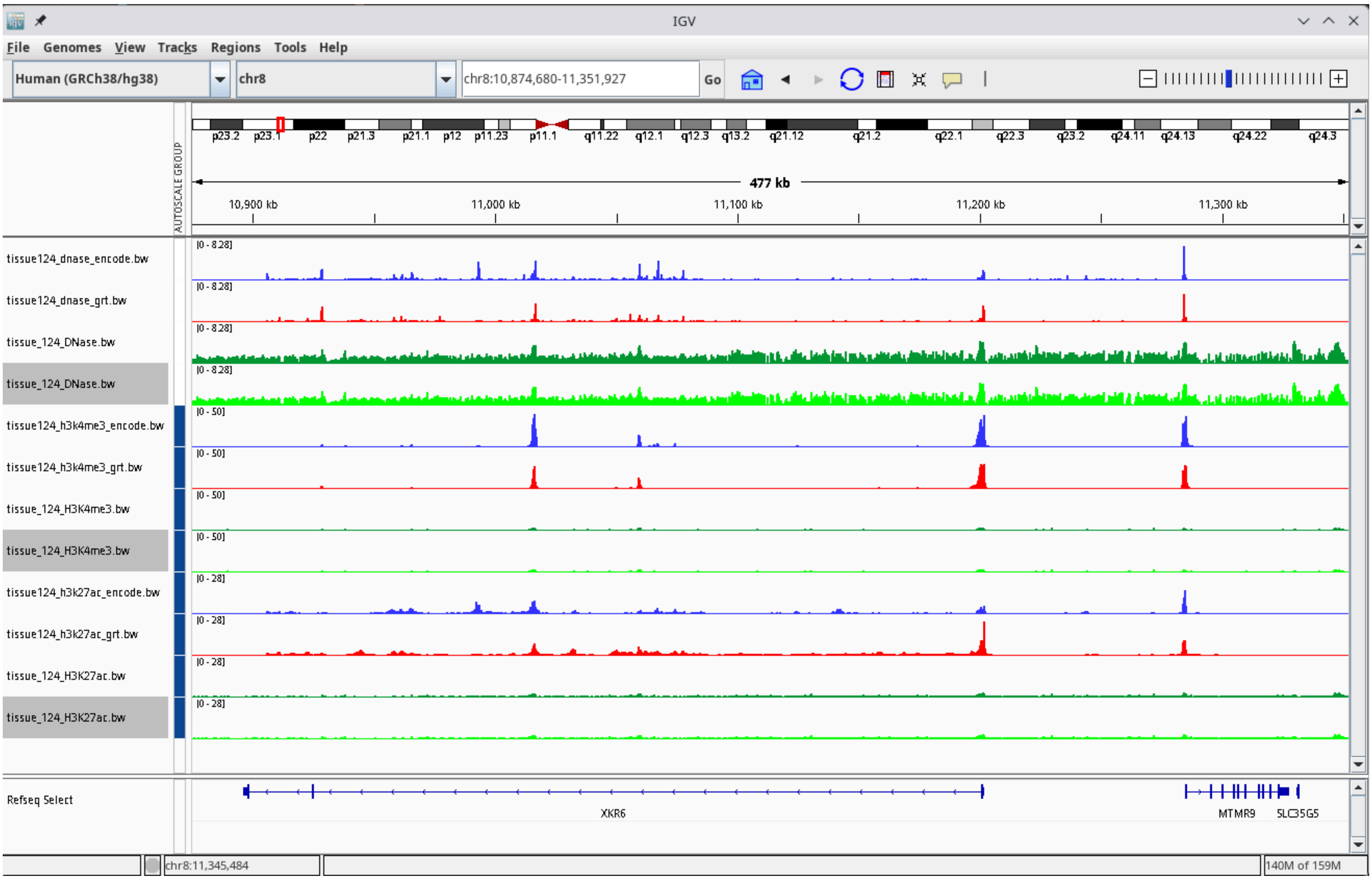

To study this further, we trained our own version of EpiGePT from scratch for the new data imputation section, on the exact same training and validation data, and made sure optimization converged with rigorous validation. As reported in R3.3, Corgi+ significantly outperforms EpiGePT (scratch) in this benchmark.

Considering all of the evidence that we report, we conclude that the Corgi family is superior to EpiGepT in many critical aspects: higher accuracy and signal to noise, better data imputation performance, higher resolution and context window and more stringent training/test split setup.

R3.9. Figure 3: Corgi's "Cross-sequence" performance appears comparable to Borzoi's across many assays, with notable exceptions of CAGE and ATAC (panel (c)). An explanation of this discrepancy would be useful. Could it be due to differences in the models' target resolution (64 bp vs. 32 bp)?

This discrepancy stems from different data processing (squashed scale) parameters between Corgi and Borzoi, as the rank-based Spearman correlations are identical. In order to avoid any confusion, we removed Pearson correlation plots from this figure and report only rank correlation which is robust to scale differences. Regarding resolutions, Figure 3 already showed correlations at the

correct resolution (128 bp for EpiGePT and 64 bp for Corgi). We have labeled the used resolutions on top of the violin plots in Figure 3a and 3b (see above, R1.3).

It is our policy to sign reviews: Alexander Floren & Katie Pollard

Reviewer #3 (Remarks on code availability):

R3.10*. Model weights and code are available. There is no installation info, tutorial, etc. So, it would be challenging for some one else to use the code. The configuration file points to specific data files in the repo, so would need to be updated if a user wanted to train their own model. The submission script is specific to SLURM. These are probably OK, but should be explained (e.g., in an added tutorial).*

We thank the reviewer for their evaluation of our code and their suggestions. We improved the installation information and the tutorial in our GitHub repository at https://github.com/ekinda/corgi. It is expected that users update the paths according to their own storage setup.

Furthermore, we have added a new functionality to the model trainer with a `training_mode` parameter. When this parameter is set to "slurm", distributed data processing (DDP) is used to scale model training to multiple GPUs. And when not, a single GPU is used.

Reviewer #4 (R4)

I co-reviewed this manuscript with one of the reviewers who provided the listed reports. This is part of the Nature Communications initiative to facilitate training in peer review and to provide appropriate recognition for Early Career Researchers who co-review manuscripts.

We thank the reviewer for their assessment of our manuscript and their contributions.

References

1. Nair, S., Kim, D. S., Perricone, J. & Kundaje, A. Integrating regulatory DNA sequence and gene expression to predict genome-wide chromatin accessibility across cellular contexts. *Bioinformatics* **35**, i108–i116 (2019).
2. Gao, Z., Liu, Q., Zeng, W., Jiang, R. & Wong, W. H. EpiGePT: a pretrained transformer-based language model for context-specific human epigenomics. *Genome Biol.* **25**, 310 (2024).
3. Murphy, A. E., Beardall, W., Rei, M., Phuycharoen, M. & Skene, N. G. Predicting cell type-specific epigenomic profiles accounting for distal genetic effects. *Nat. Commun.* **15**, 9951 (2024).
4. Hingerl, J. C. *et al.* scooby: Modeling multi-modal genomic profiles from DNA sequence at single-cell resolution. *bioRxivorg* 2024.09.19.613754 (2024) doi:10.1101/2024.09.19.613754.
5. Zhang, Z., Feng, F., Qiu, Y. & Liu, J. A generalizable framework to comprehensively predict epigenome, chromatin organization, and transcriptome. *Nucleic Acids Res.* **51**, 5931–5947

(2023).
6. Perez, E., Strub, F., Vries, H. D., Dumoulin, V. & Courville, A. C. FiLM: Visual reasoning with a general conditioning layer. *National Conference on Artificial Intelligence* 3942–3951 (2017).
7. Brock, A., Donahue, J. & Simonyan, K. Large scale GAN training for high fidelity natural image synthesis. *arXiv [cs.LG]* (2018).
8. Yang, D.-H. & Chang, J.-H. FiLM conditioning with enhanced feature to the transformer-based end-to-end noisy speech recognition. in *Interspeech 2022* 4098–4102 (ISCA, ISCA, 2022).
9. Shiraki, T. *et al.* Cap analysis gene expression for high-throughput analysis of transcriptional starting point and identification of promoter usage. *Proc. Natl. Acad. Sci. U. S. A.* **100**, 15776–15781 (2003).
10. Kawaji, H. *et al.* Comparison of CAGE and RNA-seq transcriptome profiling using clonally amplified and single-molecule next-generation sequencing. *Genome Res.* **24**, 708–717 (2014).
11. Gacita, A. M. *et al.* Altered enhancer and promoter usage leads to differential gene expression in the normal and failed human heart. *Circ. Heart Fail.* **13**, e006926 (2020).
12. Linder, J., Srivastava, D., Yuan, H., Agarwal, V. & Kelley, D. R. Predicting RNA-seq coverage from DNA sequence as a unifying model of gene regulation. *Nat. Genet.* 1–13 (2025).

Response to Reviewers

Context-aware sequence-to-function model of human gene regulation
Ekin Deniz Aksu and Martin Vingron

2nd Round of Peer Review

We thank all reviewers for their continued assessment of our work and we are grateful for their contributions in improving the quality of our manuscript. In the second round of revision, we incorporated the reviewer's remaining suggestions into our manuscript, providing additional clarity to the text and an additional figure in Fig. S11. We also fixed labeling in Fig. 4b and Fig. 7a, and we added three new references to the text.

Below, we reproduce the reviewers' comments verbatim and provide point-by-point responses.

Reviewer #1

The revised manuscript has substantially improved in overall quality, and the authors' careful efforts in addressing the reviewers' comments are appreciated. Despite these improvements, the current benchmarking remains relatively limited, as the study only compares Corgi with EpiGePT and Borzoi. I recommend expanding the comparison to include additional relevant methods in order to strengthen the credibility of the evaluation.

Specific suggestions are as follows:
1. Include comparisons with EPCOT and EnformerCelltyping
Although Corgi uses RNA-seq expression data while EPCOT and EnformerCelltyping rely on different modalities of cell-state information, such as chromatin accessibility, including these methods in the comparison would still provide valuable context. Evaluating models across different conditioning modalities could help assess performance from complementary perspectives and provide a more informative background for understanding the potential advantages of Corgi.

2. Evaluate these baseline models under the cross–cell-type setting
In their response, the authors note that EnformerCelltyping requires local chromatin accessibility information and therefore cannot be applied to evaluation settings where sequence information is completely held out (e.g., cross-sequence or cross-both). This limitation is understandable. However, both EnformerCelltyping and EPCOT can still be evaluated under the cross–cell-type setting, which is also the evaluation scenario adopted in the current study. Therefore, including these methods as baselines in this setting should be technically feasible.

Overall, incorporating these additional comparisons would strengthen the benchmarking framework and provide a clearer reference for understanding the relative advantages of different types of cell-state information (e.g., RNA expression versus chromatin accessibility) in sequence-to-function prediction tasks.

This review has taken into consideration the comments raised by Reviewer #2, and the points discussed in my report were intended to address both my own evaluation and the issues highlighted by Reviewer #2.

We thank the reviewer for their comments. Below we reproduce our response that was relayed to the editor, where we reasoned that comparisons to approaches with substantial differences in input modality (RNA-seq versus ATAC/DNase-seq) are beyond the scope of this study.

EnformerCelltyping and EPCOT use DNA accessibility information, while Corgi uses (a subset of) RNA expression data. DNA accessibility is a much richer source of information since measuring genome-wide accessibility levels is informative for both gene expression (through promoter accessibility) and also other cis-regulatory elements such as enhancers. Therefore they cannot be compared to Corgi head-to-head in a fair manner, and benchmarks would be inconclusive. On the contrary, it would contradict the efforts we put into interpreting the role of RNA expression at the request of the reviewers in the first round.

Further, the claim that we only compare Corgi against two models, EpiGePT and Borzoi, is not founded. In fact, in the revised manuscript we compare against 4 models: pretrained EpiGePT (published version), a version of EpiGePT we trained ourselves from scratch, Borzoi (published) and Avocado (trained from scratch). This is a very comprehensive benchmarking scheme, since i) EpiGePT is the direct competitor to Corgi, as it is the latest model that predicts epigenomic tracks using transcription factor expression, and ii) Avocado is the state-of-the-art in data imputation, and has a very different matrix factorization approach. Since Corgi significantly outperforms both models, we can say that Corgi represents a significant advance in the context-aware DNA sequence modeling and epigenomic data imputation fields.

The editor has informed us that the reviewer is satisfied with our response.

Reviewer #3

The authors conducted analyses to address many of our comments. Responses for R3.4, R3.6, R3.9, and 3.10 are sufficient. However, in other cases the specific reviewer question was not directly answered or the answer was only shared in the response document and was not used to improve the clarify of the manuscript. Corgi is an exciting new modeling framework, but additional results and text details are needed in order to clarify it performance, especially on gene expression prediction tasks. We therefore make the following suggestions for further revision, organized using the numbering system from the authors' response document.

We thank the reviewer for their continued assessment of our work. Below we took care to directly answer specific questions and to use the answers to improve the manuscript.

We would like to begin with an overall clarification. As the reviewer mentions, many of the reviewer's remaining points are concerning performance in gene expression prediction benchmarks. We want to underline that the models introduced by our paper have two main use cases they focus on: Corgi on cross-both generalization, and Corgi+ on data imputation. Since in both cases RNA expression is already part of the input to Corgi through *trans*-regulator expression values, predicting RNA expression is not the main focus.

R3.1. We acknowledge the reasons given for CAGE + RNA data "harmonization" but reiterate that this justification should be included in the main text.

We added the following sentence to the main text:

> "Harmonization reduced batch effects between different assay types, and improved correlation between assays with the most striking examples being CAGE-seq vs RNA-seq. **These assays both measure the concentration of mRNA in the cell and are shown to have strong correlation. (Kawaji et al. 2014; Gacita et al. 2020)**"

R3.2. Rather than (or in addition to) the number of windows, we ask the authors to state the amount of unique (i.e. non-overlapping) sequence (in bp), as well as the number of genes present in the validation and test sets after removal of trans-regulator gene bodies and re-tiling of the remaining sequences. This is important because windows were redefined, and they overlap, making it impossible to infer these two key numbers from the window count. These two statistics should be reported and compared against the equivalent statistics for Borzoi's validation and test sets. The Methods paragraph edited in response to the initial comment is a reasonable place to put this information.

The amount of unique, non-overlapping training and test sequences (in bp) are stated in the Methods section of the manuscript. To this sentence, we added the length of the validation set as well. Furthermore, we added the sequence in base pairs that is removed from the validation and test folds due to overlaps to trans-regulator genes.

> " [...] Then, we subtracted trans-regulator gene coordinates from the merged regions from folds 3 and 4 using bedtools subtract, **which subtracts 18 Mb of sequence from fold 3 and 20 Mb from fold 4.** [...] **In total, we train on 2.2 Gbp of DNA sequence, validate on 298 Mbp and test on 292 Mbp.**"

The number of genes present in the test sequences that we used to evaluate Corgi's gene expression prediction performance are stated in Figure 2e and 2f. These are 843 genes used in the cross-sequence and cross-both benchmarks, and 2696 genes in the cross-cell type benchmark. We added a further sentence to the Benchmarking subsection of Methods:

> "For gene-level benchmarking, we measured the sum of predicted vs observed coverage overlapping with gene coordinates in RNA-seq tracks, and log-transformed the values. Genes with zero expression across all samples were excluded, in order to avoid an inflation in reported accuracy values. **This results in 843 genes for the cross-sequence and cross-both benchmarks, and 2696 genes for the cross-cell type benchmark (Fig. 2e,f).**"

R3.3. The new baselines outlined in Figure 4a are very interesting, and they are important for evaluating imputation performance (per other reviewer comments). However, our original comment suggested something different. We ask about a baseline using mean gene expression plus sequence and no other inputs as a comparator for gene expression prediction tasks—not for imputation of chromatin marks. It is recommended that this evaluation is added, because it will enable the authors to illustrate the benefit of including trans regulator expression.

We performed this suggested analysis by comparing gene expression prediction performance of the mean baseline (average total RNA-seq track of the training samples), Corgi with the average

trans-regulator expression of the training samples as input, Corgi with correct trans-regulator expression input, and Corgi+. In order to show the effect of using gene bodies vs exons (as per 3.7 below), we also repeated this analysis using both gene bodies and exons to calculate gene expression (see response to 3.7). This analysis was performed on the same prediction tracks calculated for Figure 4.

As seen in the figure below, *Corgi* outperforms *Corgi (avg. trans)* in this benchmark as well, with 0.89 vs 8.865 (Spearman rho). Next comes the mean baseline with 0.93, and finally Corgi+ is at 1.0, since it also has the local RNA-seq track in its input. Consistent with our findings in epigenomic predictions in Figure 4, the mean baseline does very well in ranking genes according to raw expression, however, it is unable to predict cell type-specific differences (mean-subtracted correlations), which is our main focus in the cross-cell benchmark. In mean subtracted correlations, we see that the margin between *Corgi* and *Corgi (avg. trans)* is wider, with 0.36 vs 0.21 (Spearman rho).

We incorporated Response Figure 1 as Figure S11 in our manuscript.

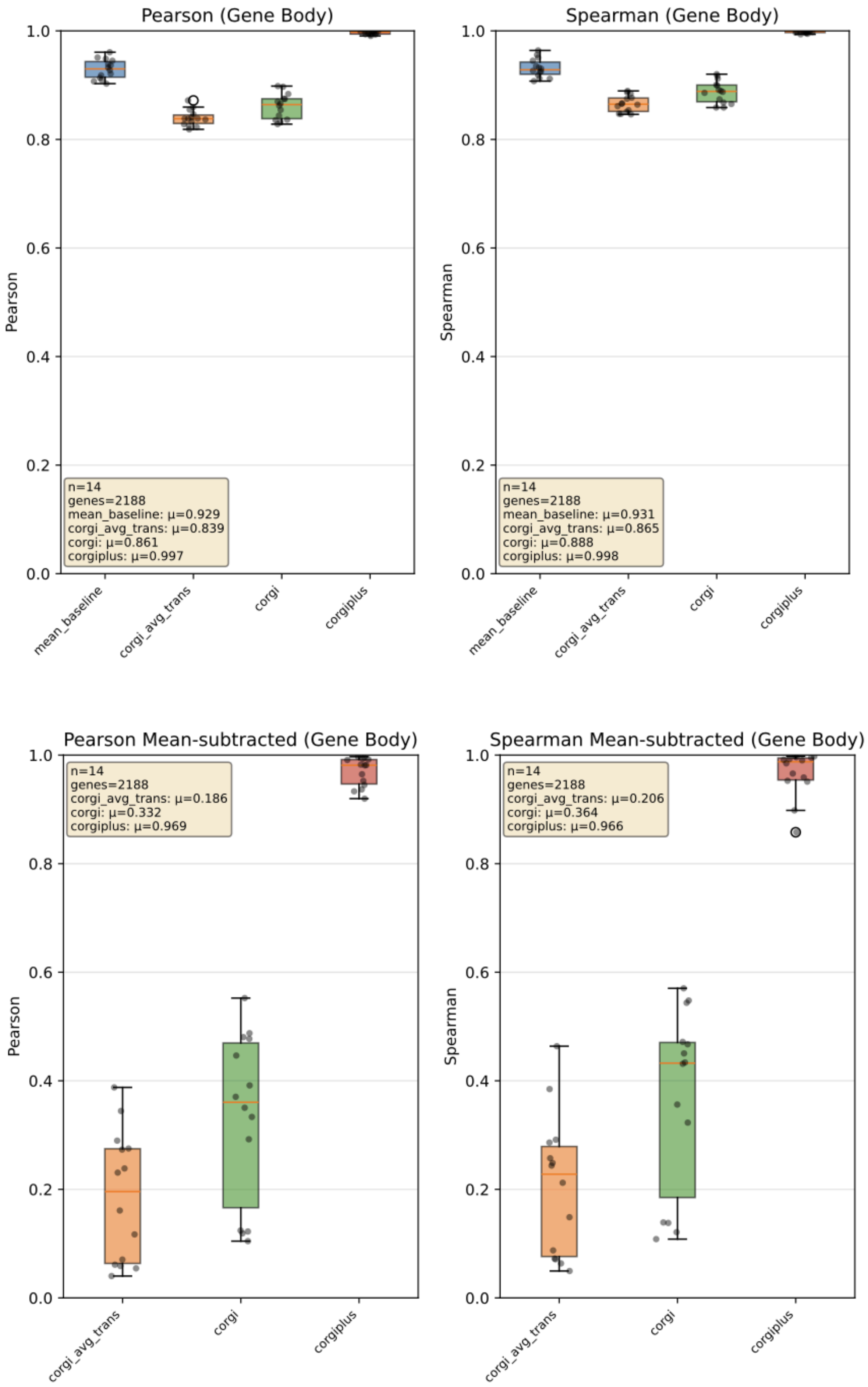

Response Figure 1

Additionally, we recommend rigorously defining "mean baseline"; the description "mean training baseline signal from all Corgi tracks" is not sufficiently detailed to tell what was actually done (are different assays averaged or all cell types per assay type or something else, and is this done per bin for a given window or is it a genome-wide average?). Please clarify what tracks go into the mean. An example might be helpful, such as "for example, to predict H3K27ac for a given window in a specific cell type, we use the mean value of observed H3K27ac tracks per bin in this window across all cell types in our catalog".

We thank the reviewer for this suggestion. We have incorporated their suggestion into the main text in the relevant section:

> "Further, we trained "*Corgi-impute*", which uses local RNA-seq coverage tracks at 64 bp resolution, as well as the mean training baseline signal from all Corgi tracks. The mean training baseline signal is assay-specific, and simply averages signal from a given assay across samples in the training set. For example, for the DNase channel in a given genomic window, it corresponds to the average of observed DNase signal in the same window across all cell types in the training set."

R3.5. The authors clarified their clustering methods for selecting cell types to hold out. This is clear now.

Regarding the easy-test set, the authors reported (in the response document) gene-level Pearson correlation distributions for three expression prediction tasks. They performed a statistical test (not sure which one, please add this detail) comparing the cross-sequence and cross-both Pearson correlation distributions over easy-test examples, and they argue that the lack of significant difference between these two means that similarity to training data is not driving performance. These results are useful but do not directly address the original recommendation. Perhaps we were unclear; we did not specify which metrics we wanted to see. To clarify, our suggestion was to compare gene-level Pearson and Spearman correlation distributions for the easy-test set versus the test set and the versus the training set on the same tasks: cross-sequence, cross-cell type, and cross-both.

Further, the Pearson correlations reported for the "easy-test" set are somewhat lower than expected based on other results in the manuscript. Is it possible that the figure used the "mean-subtracted" expression values rather than the raw values? It would be good to clarify this.

We would like to begin with clarifying our argument in the revised manuscript. We argued that since we do "not observe significant performance differences between the cross-sequence and cross-both easy-test settings", easy-test samples are -as expected- easy, and they do not constitute proper cellular context holdouts. So, contrary to the reviewer's summary above, we tried to argue that the similarity to training data results in high performance in the easy-test set. We find that this line of argument is indeed unclear in the revised manuscript and we are sorry for the confusion. We updated the relevant section accordingly:

> "In practice, we did not observe significant performance differences between the cross-sequence and cross-both easy-test settings (mean r 0.408 vs 0.378, p=0.068, pooled all tracks, t-test), suggesting that this hold-out set is almost as easy as using training cell types, and thus did not perform additional analyses on this set."

Regarding the exact numbers, in the first round of revision, the reviewer's request regarding the easy-test set performance did not specifically mention gene expression predictions. Therefore we pooled results across all prediction tracks and performed a t-test, which results in the reported numbers. This detail is now reflected in the updated manuscript.

Finally, as suggested by the reviewer, we performed a new and systematic analysis, extending the gene-level correlation analysis to include the easy-test set. We report both Pearson, Spearman and also both raw and mean-subtracted correlations, for the sake of completion.

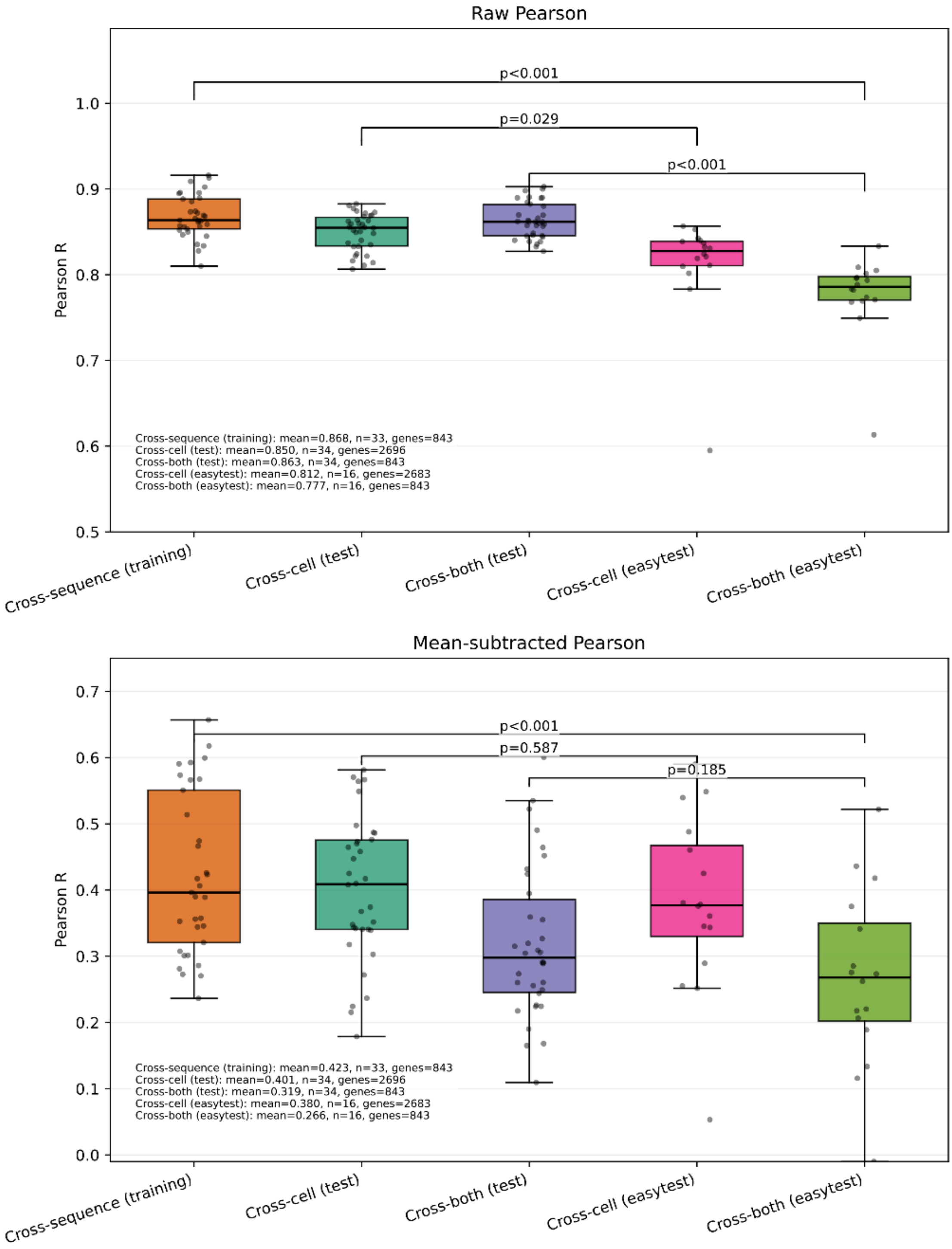

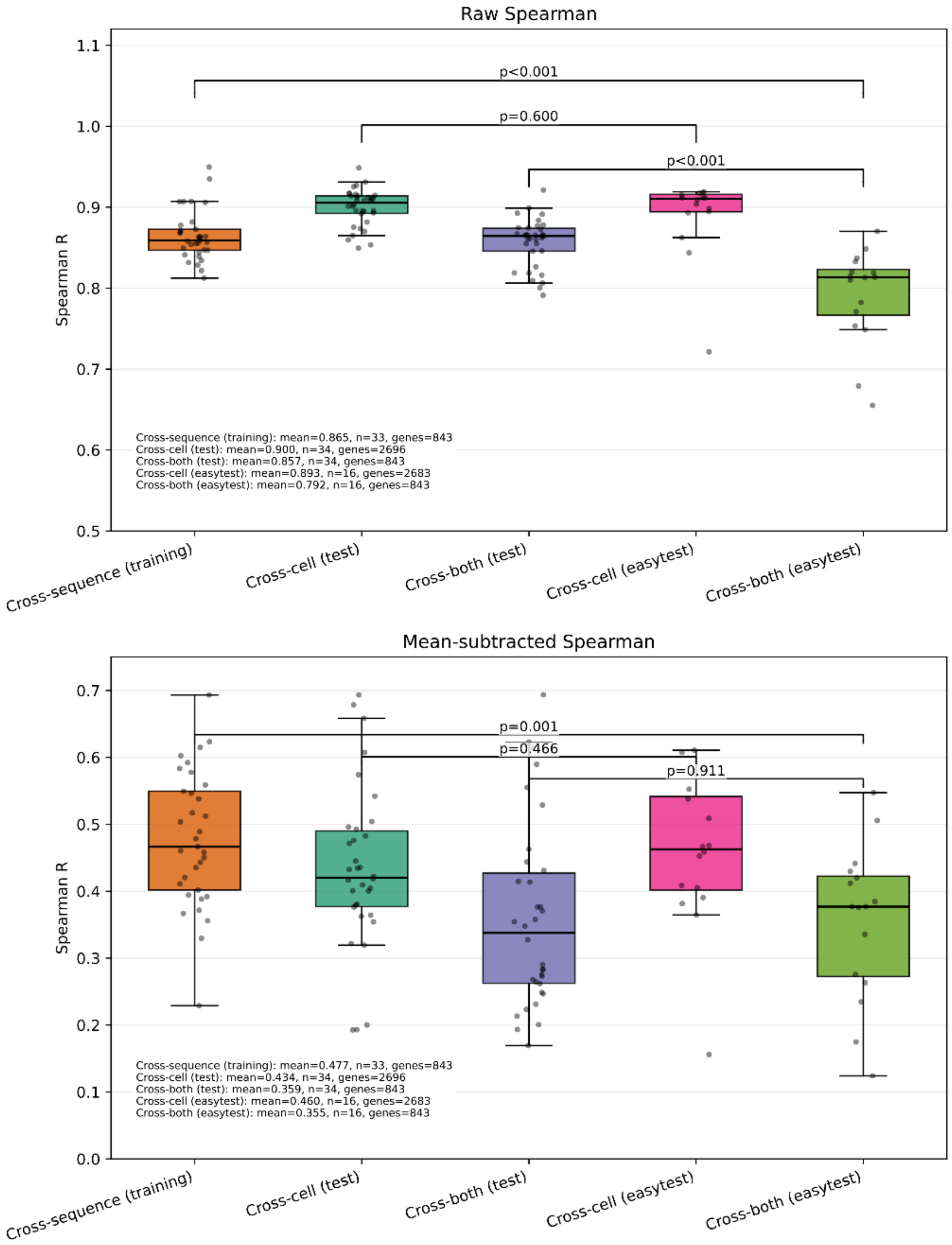

Response Figure 2

Here, there are three relevant comparisons: cross-sequence (training samples, test genes) vs cross-both (easy-test samples, test genes), cross-cell (test samples) vs cross-cell (easy-test samples), and cross-both (test samples) vs cross-both (easy-test samples), and we performed t-tests between these score distributions. In raw correlations, both Spearman and Pearson correlations are higher in cross-sequence compared to the cross-both easy-test setting. There is most commonly no statistical difference between cross-cell (test) and cross-cell (easy-test), and cross-both (test) has higher performance than cross-both (easy-test), except for the mean-subtracted setting. Though interpretation of these results is complicated by the fact that the number of samples with available ground truth RNA-seq data is lower in the easy-test set.

R3.7. The results from Figure 2e-f should be contextualized against Borzoi's performance as in the new supplementary Figure 5, but using Pearson's correlation (for consistency with other figures and publications). Corgi's "mean-subtracted" cross-sequence performance lags substantially behind that of Borzoi, although Borzoi of course lacks the ability to generalize to new cell types. This an important trade-off and should not be relegated to the supplement.

We would like to point out that this benchmark is done to see whether one suffers an accuracy disadvantage over Borzoi when using Corgi in the training contexts. This scenario is not a main use case of Corgi and is done as a sanity check. Since Corgi uses RNA-seq as one of its inputs, predicting RNA expression is an artificial testing scenario and there is no practical trade-off.

Second, using Pearson correlation in this setting is risky, since different publications use different ways to "squash" genomic tracks to reduce the dynamic range. For instance, Borzoi uses the following complex transformation: "we normalized each coverage track by exponentiating its bin values by 3/4. If bin values were still larger than 384 after exponentiation, we applied an additional square-root transform to the residual value." Avocado uses an arcsinh-transformation. Therefore, performance claims are not easily comparable between different papers. Using Pearson correlation is unfair to different models even after rescaling to raw values, since different models are trained with different "squashing" parameters. The fair way to compare is with rank-based correlation, which we performed here.

Finally, on the suggestion of the reviewer, we expanded the main text by including the mean-subtracted result to the relevant section as well.

> "In gene-level expression correlations Borzoi has a measurable edge over Corgi (0.87 vs 0.84, Figure S5c) as well as in mean-subtracted gene-level correlations (0.47 vs 0.42, Figure S5d)."

The authors note that they switched their method of calculating gene-level expression from aggregating over exons to aggregating over entire gene bodies. Why was this done? This change makes it harder to compare against existing methods (like Borzoi), and could also influence the reported performance. We recommend showing results for both aggregation approaches or providing a strong justification for the switch.

As described in our initial response, our motivation in switching to gene bodies was that most RNA-seq samples in our dataset use total RNA capture, rather than polyA-enriched RNA capture. This results in intronic reads from gene bodies, which contain meaningful signals. We would like to clarify that this does not reduce comparability to Borzoi in Figure S5, as we apply the same aggregation approach to both models.

Empirically, using gene bodies indeed increases performance slightly. In Response Figure 3 below, we see the same analysis from Response Figure 1, using exons instead of gene bodies for aggregation. This results in slight decreases overall (e.g. for Corgi, 0.89 to 0.84 in Spearman rho). The ranking of baselines or models does not change.

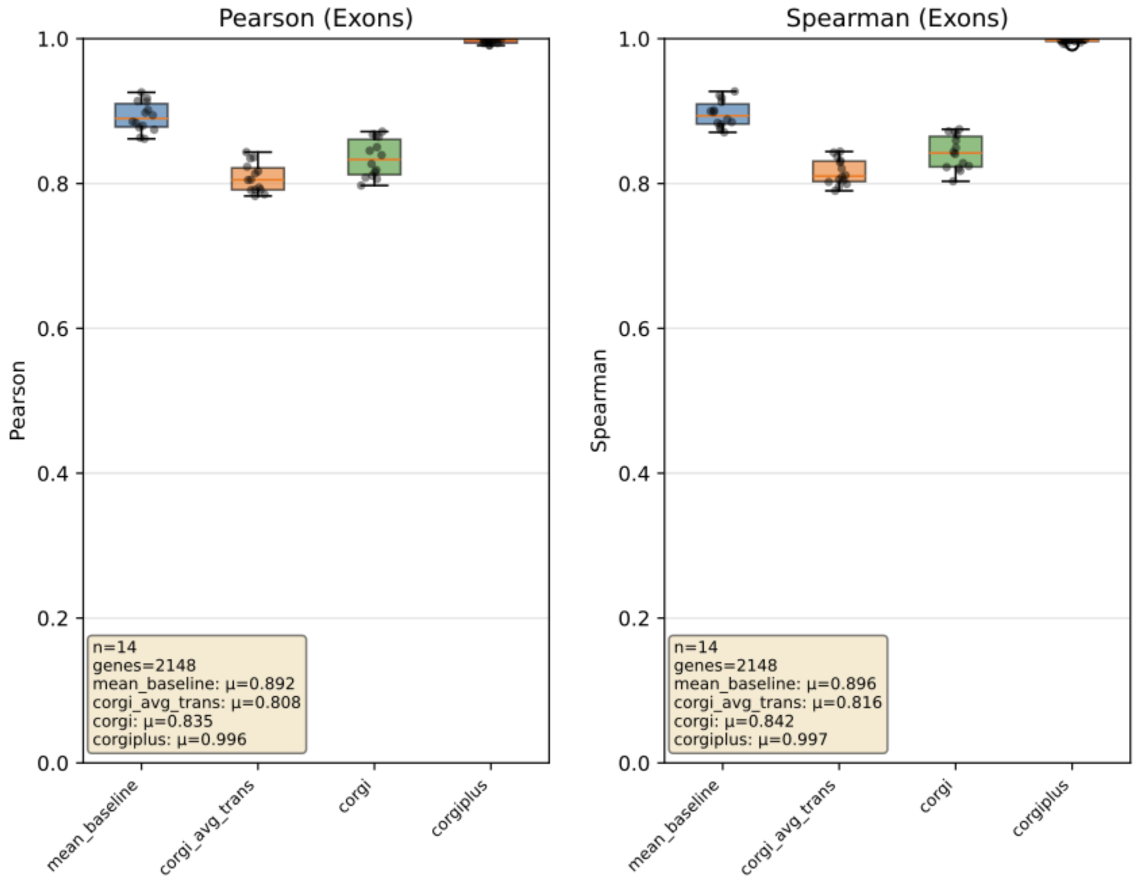

Response Figure 3

Finally, regarding transformation of the RNA-seq data, it is important to specify what log base and what pseudocount were used. The example scatterplot (Fig. 2h) shows a large gap between data points with measured expression of 0 and the next lowest value, with measured expression of ~4. In our experience, the choice of pseudocount can affect the resulting Pearson correlation in situations like this.

We have added this detail to the relevant section of the Methods:

> "For gene-level benchmarking, we measured the sum of predicted vs observed coverage overlapping with gene coordinates in RNA-seq tracks, and log-transformed the values **using numpy.log1p (base = e, pseudocount = 1)**."

It would also be helpful to display the underlying data points instead of just showing densities in Fig. 2e-f.

We have included the underlying data points in the density plots in Fig. 2e-f as the reviewer suggests. We updated the figure caption accordingly.

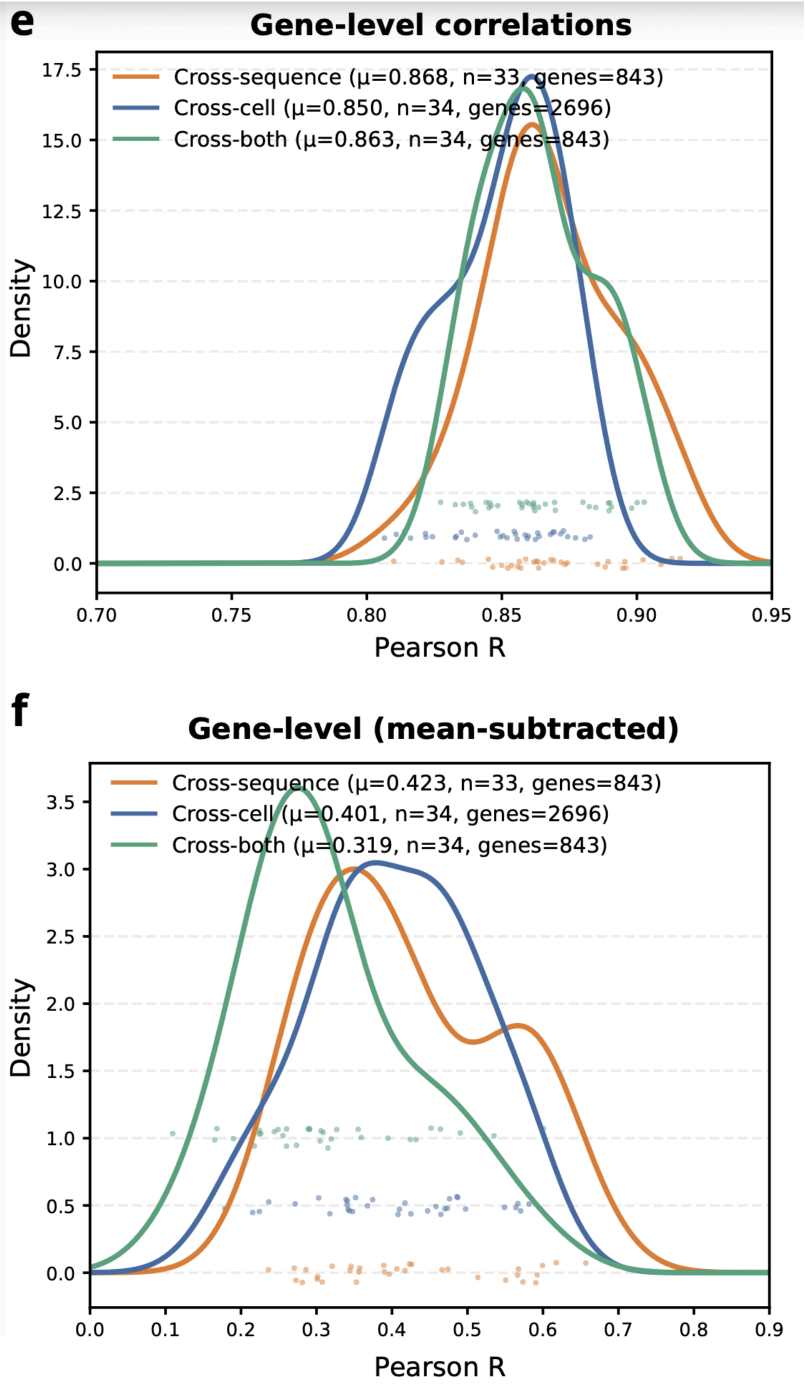

Figure 2e and 2f

R3.8. Given that the changes made to the EpiGePT pipeline "did not visibly change its predictions", could there be remaining bugs?

We double-checked the code and did not find any remaining bugs.

It is our policy to sign reviews: Alexander Floren & Katie Pollard

Reviewer #3 (Remarks on code availability):

The code was modified to have an option to run via SLURM or on a local GPU. This is a welcome addition.

Reviewer #4

I co-reviewed this manuscript with one of the reviewers who provided the listed reports. This is part of the Nature Communications initiative to facilitate training in peer review and to provide appropriate recognition for Early Career Researchers who co-review manuscripts.

We thank the reviewer for their contributions to the peer review process.

Response to Reviewers

Context-aware sequence-to-function model of human gene regulation

Ekin Deniz Aksu and Martin Vingron

3rd Round of Peer Review

We thank all four reviewers for a constructive peer review process.

Our responses to the remaining reviewer comments are below.

Reviewer #3

The authors conducted analyses and added text to address our comments. The revision is much improved with these details added. We have two minor but important suggestions:

R3.5 from prior number system: To contextualize the new results for cross-sequence and cross-both (easy-test) performance, it is important to report alongside these the pooled all tracks cross-both (test) performance.

We reported the pooled all tracks cross-both (test) performance in the relevant section.

"cross-sequence mean *r* = 0.408 vs cross-both easy-test 0.378, t-test p=0.068, pooled all tracks; **while cross-both test mean *r* = 0.343, t-test p=0.000005 vs cross-sequence**"

General: With respect to imputation, it is appropriate to say that Corgi/Corgi+ is cutting-edge. But with respect to gene expression prediction, performance is not notably better than sequence-only models. Hence, we recommend toning down phrases such as "state-of-the-art performance" and "experimental-level accuracy" when referencing gene expression prediction.

We have toned these phrases down in the text.

Before: [...] Corgi approaches experimental-level accuracy in gene expression predictions in held-out cell types, while achieving state-of-the-art performance in joint cross-sequence and cross-cell type epigenetic track prediction.

After: [...] **Corgi achieves state-of-the-art performance in joint cross-sequence and cross-cell type epigenetic track prediction.**

and

Before: Remarkably, Corgi's performance remains robust even on the toughest benchmarks which test the model on held-out sequences and held-out cell types, where Corgi sets a new state-of-the-art prediction accuracy.

After: Remarkably, Corgi's performance remains robust even on the toughest benchmarks which test the model on held-out sequences and held-out cell types, **where Corgi significantly outperforms EpiGePT.**

General: We agree that predicting gene expression with RNA as a model input is not useful from the perspective of the predictions themselves. But such a model might be useful for learning gene regulatory mechanisms, in this case cell type specific gene expression. The authors may want to speculate about this in the discussion or results.

It is our policy to sign reviews: Alexander Floren & Katie Pollard

Reviewer #3 (Remarks on code availability):

No new comments on the code.

Reviewer #4
I co-reviewed this manuscript with one of the reviewers who provided the listed reports. This is part of the Nature Communications initiative to facilitate training in peer review and to provide appropriate recognition for Early Career Researchers who co-review manuscripts.
